# Investigation of All Disease-Relevant Lysine Acetylation Sites in α-Synuclein Enabled by Non-canonical Amino Acid Mutagenesis

**DOI:** 10.1101/2025.01.21.634178

**Published:** 2025-08-22

**Authors:** Marie Shimogawa, Ming-Hao Li, Grace Shin Hye Park, Jennifer Ramirez, Hudson Lee, Paris R. Watson, Swati Sharma, Zongtao Lin, Chao Peng, Virginia M.-Y. Lee, Benjamin A. Garcia, David W. Christianson, Elizabeth Rhoades, David Eliezer, E. James Petersson

**Affiliations:** 1 Department of Chemistry, School of Arts and Sciences, University of Pennsylvania, 231 South 34th Street, Philadelphia, PA 19104, USA; 2 Department of Biochemistry, Weill Cornell Medicine, 1300 York Avenue, New York, NY, 10065, USA; 3 Graduate Group in Biochemistry, Biophysics, and Chemical Biology, Perelman School of Medicine, University of Pennsylvania, 206 Anatomy-Chemistry Building, 3620 Hamilton Walk, Philadelphia, PA 19104, USA; 4 Department of Biochemistry and Molecular Biophysics, Washington University in St Louis, 4523 Clayton Ave, St Louis, MO 63130, USA; 5 Department of Neurology, David Geffen School of Medicine, University of California - Los Angeles, 710 Westwood Plaza, Room C-224, Los Angeles, CA 90095, USA; 6 Department of Pathology and Laboratory Medicine, Center for Neurodegenerative Disease Research, University of Pennsylvania, 3600 Spruce Street, Philadelphia, PA 19104, USA; 7 Department of Biochemistry and Biophysics, Perelman School of Medicine, University of Pennsylvania, 421 Curie Boulevard, Philadelphia, PA 19104, USA

## Abstract

Aggregates of α-synuclein (αS) are hallmarks of synucleinopathies, including Parkinson’s Disease (PD) and Multiple System Atrophy (MSA). We have recently shown that αS lysine acetylation in the soluble monomer pool varies between healthy controls, PD, and MSA patients. To study the effects of lysine acetylation at all disease-relevant sites of αS, we first compared production of acetylated αS through either native chemical ligation or non-canonical amino acid (ncAA) mutagenesis. Since yields were comparable, ncAA mutagenesis was deemed superior for scanning many acetylation sites. We expressed and purified 12 disease-relevant variants and studied their binding to membranes as well as their aggregation propensities, and found that acetylation of lysine 12, 43, and 80 had particularly strong effects. To understand the implications for acetylation of monomeric αS found in healthy cells, we performed NMR experiments to study protein conformation and fluorescence correlation spectroscopy experiments to quantify lipid binding. We also investigated the effects of acetylation at lysine 12, 43, and 80 on fibril seeding in neurons. Collectively, our biochemical and cell biological investigations indicated that acetylation of K_80_ could inhibit aggregation without conferring negative effects on monomer function in healthy cells. Therefore, we studied the structures of fibrils with K_80_ acetylation through cryo-electron microscopy to uncover the structural basis for these effects. Finally, we identified inhibition of HDAC8 as a way of potentially increasing acetylation at K_80_ and other inhibitory sites for therapeutic benefit.

## Introduction

α-Synuclein (αS) is a 14 kDa protein that typically exists at presynaptic terminals in healthy neurons, where its primary function is believed to be in synaptic vesicle trafficking and regulating neurotransmission(Burré et al., 2010; Cabin et al., 2002). Aggregates of αS commonly characterize several neurodegenerative diseases such as Parkinson’s Disease (PD), Dementia with Lewy Bodies (DLB) and Multiple System Atrophy (MSA), which are referred to as synucleinopathies. Evidence indicates that distinct pathology is caused by αS fibrils formed in different disease environments, or αS “strains.” Aggregation seeding experiments showed that αS strains have distinct abilities to propagate pathology, where αS fibrils from MSA patients are much more potent in seeding aggregation than those from DLB(Peng et al., 2018). In addition to this, recent cryo-electron microscopy (cryo-EM) experiments showed that structures of αS fibrils vary between different pathological contexts in PD/DLB(Yang et al., 2022) and MSA(Schweighauser et al., 2020). Despite these findings, the mechanism underlying these differences remains to be understood. It has been suggested that post-translational modifications (PTMs) may contribute to these differences(Baskakov, 2021). Among the PTMs that have been studied on αS thus far are *N*-terminal acetylation, phosphorylation, O-GlcNAcylation, lysine acetylation, lysine ubiquitination, tyrosine nitration and glutamate arginylation(Baskakov, 2021; Chen et al., 2019; Pancoe et al., 2022; Schaffert and Carter, 2020).

We have recently published a comprehensive study of the relative levels of PTMs in the soluble αS monomer pool between MSA, PD, and DLB patients vs. healthy controls(Zhang et al., 2023). While many of the PTMs identified have been previously studied in chemical detail by our laboratory and others,(Moon et al., 2021; Pancoe et al., 2022) lysine acetylation stood out as a PTM that is very common and highly physiologically relevant in other proteins, but had received relatively little attention to date in the context of αS. Given that other αS PTMs have found great significance as biomarkers (e.g. pS_129_ – a hallmark of PD(Fujiwara et al., 2002)) and drug targets (e.g. kinase inhibitors(Pagan et al., 2019)), we wished to investigate these acetyl lysine (^Ac^K) sites more thoroughly.

Lysine acetylation is a reversible PTM that can be introduced at specific sites by lysine acetyltransferases (KATs) or non-enzymatically added by reaction with abundant cytosolic acetyl coenzyme A. Lysine deacetylation is catalyzed by lysine deacetylases (KDACs), which include Zn^2+^-dependent histone deacetylases (HDACs) and NAD^+^-dependent sirtuins(Ruijter et al., 2003; Sauve et al., 2006). In addition to our comprehensive PTM study in patient samples, there has been some previous evidence for the role of lysine acetylation in synucleinopathies. It has been suggested that activity imbalances between KATs and KDACs on histone or non-histone proteins are pathologically relevant to PD. In fact, activators of some sirtuins and inhibitors of specific KDACs/KATs have shown potential as therapeutics(Wang et al., 2020). Identified as a substrate of these enzymes, αS was found acetylated on Lys6 and Lys10 in mouse brain. Sirtuin-2 was found to deacetylate those sites and enhance the toxicity of αS(de Oliveira et al., 2017). It is notable that in this work, semi-synthetic, acetylated αS was used for the deacetylation assay, however for other experiments glutamine was used to mimic lysine acetylation, which is a common strategy of choice in the field of biochemistry or biophysics, due to easier access to the site-specifically, homogenously modified construct.

**Figure 1.**
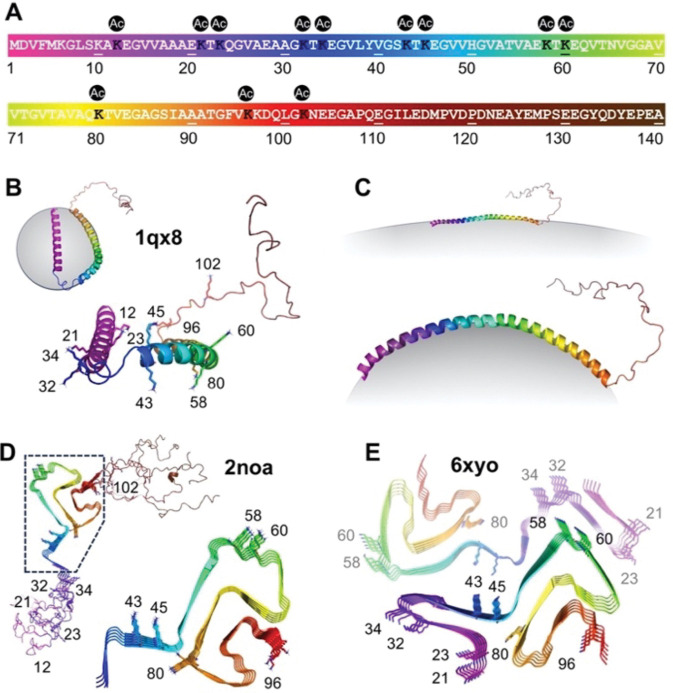
Neurodegeneration-relevant Lys acetylation sites in αS. (A) αS sequence with positions 12, 21, 23, 32, 43, 45, 58, 60, 80, 96 and 102 marked. (B) Solution NMR structure of micelle-bound αS (PDB: 1qx8). (C) Proposed structure of vesicle-bound αS. (D) Solid-state NMR structure of recombinant αS fibrils (PDB: 2noa). (E) Cryo-EM structure of MSA patient αS fibrils (PDB: 6xyo).

Recently, many more disease-relevant lysine acetylation sites have been identified in patient tissue. Eight ^Ac^K sites were identified by Goedert and Scheres in mass spectrometry (MS) studies accompanying a cryo-EM structure of αS fibrils from MSA patients (Lys21/23/32/45/58/60/80/96, Figure 1A)(Schweighauser et al., 2020). Ten ^Ac^K sites, many overlapping those found by Goedert and Scheres, were identified by MS in our previously noted studies of soluble αS from patients (Lys12/21/23/34/43/45/58/60/96/102, Figure 1A)(Zhang et al., 2023). In our accompanying mechanistic studies of the PTMs, authentic constructs of phosphorylated αS were produced through semi-synthesis because phosphorylation occurred at a few key sites with established semi-synthetic routes, but lysine acetylation was investigated only though glutamine mimics due to challenges in systematically investigating a large number of PTM sites where there was less literature to identify key targets.(Zhang et al., 2023) Thus, there have not been studies of the effect of authentic lysine acetylation in αS at the sites identified from patient tissue.

In this work, we set out to study lysine acetylation at all 12 disease-relevant sites of αS (Figure 1A). We began by comparing the efficiency of producing acetylated αS through either native chemical ligation (NCL) or non-canonical amino acid mutagenesis (ncAA mutagenesis). We found that ncAA mutagenesis provided comparable yields, and was therefore superior for scanning many acetylation sites due to the ease of generating new constructs. Once the 12 αS ^Ac^K variants were expressed and purified, we studied their binding to membranes as well as their aggregation propensities. We performed NMR, fluorescence correlation spectroscopy (FCS), and transmission electron microscopy (TEM) experiments on acetylated variants that showed perturbed membrane binding or aggregation. NMR and FCS experiments were enabled by our ncAA mutagenesis approach which made it facile to produce isotopically or fluorescently labeled αS. We went on to characterize the seeding ability of select ^Ac^K constructs in neurons, determine a cryo-EM structure of fibrils with a particular ^Ac^K site of interest, and to test HDAC selectivity in deacetylating these sites. The combination of the site-specific incorporation approach and a variety of biological characterization methods provides a systematic understanding of lysine acetylation, identifying a few key ^Ac^K sites as significant for further investigation and potential therapeutic intervention.

## Results and Discussion

### Comparison of ncAA Mutagenesis and NCL

**Figure 2.**
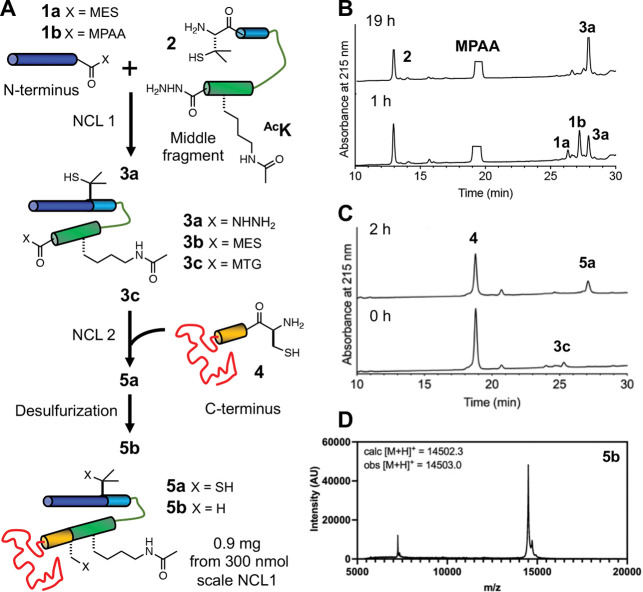
Semi-synthesis of αS-^Ac^K_80_. (A) Acetylation is introduced through peptide synthesis, and the peptide is combined with expressed peptide fragments using NCL. (B) Analytical HPLC trace for the first ligation. **1a**: αS_1–76_-MES, **1b**: αS_1–76_-MPAA, **2:** αS_77–84_-Pen_77_^Ac^K_80_-NHNH_2_, **3a:** αS_1–84_-Pen_77_^Ac^K_80_-NHNH_2_. (C) Analytical HPLC trace for the second ligation. **3b:** αS_1–84__Pen_77_^Ac^K_80_-MES, **3c:** αS_1–84__Pen_77_^Ac^K_80_-MTG, **4:** αS_85–140_-C_85_, **5a:** αS-Pen_77_C_85_^Ac^K_80_. (D) MALDI MS of HPLC-purified αS-^Ac^K_80_ (**5b**).

Protein semi-synthesis is a powerful approach to site-specifically incorporate modifications of interest into a protein sequence(Thompson and Muir, 2020) and it has been a method of choice for many αS PTM studies(Moon et al., 2021), including Lys acetylation(de Oliveira et al., 2017). To test this approach to synthesizing acetylated αS, we chose ^Ac^K_80_ as an example, and combined solid-phase peptide synthesis (SPPS),(Merrifield, 1963) by which ε-acetyllysine is incorporated, with the expression of protein fragments and a three-part NCL sequence using acyl hydrazides(Zheng et al., 2013) (Figure 2A, additional details in SI Scheme S1).

*N*-terminal thioester fragment αS_1–76_-MES (**1a**) and C-terminal fragment αS_85–140_-C_85_ (**4**) were each recombinantly expressed as a fusion with Mxe GyrA intein. The *N*-terminal thioester was generated by adding excess sodium 2-mercaptoethane sulfonate (MESNa) to cleave the intein by N,S-acyl shift.(Muir et al., 1998) (reported yield 24.1 mg/L(Pan et al., 2020a)). Endogenous methionyl aminopeptidase in *E. coli* processes the *N*-terminus of the 85–140 peptide to expose the *N*-terminal cysteine,(Xiao et al., 2010) which further reacts with aldehydes or ketones *in vivo* to form thiazolidine derivatives.(Liu et al., 2016) The thiazolidine derivatives were deprotected with methoxyamine to give a free *N*-terminal cysteine (4.40 mg/L, SI Figure S1B). The middle acyl hydrazide peptide αS_77–84_-Pen_77_^Ac^K_80_-NHNH_2_ (**2**, Pen: penicillamine(Haase et al., 2008)) was synthesized through SPPS (Yield: 12.4 mg, 12 μmol, 48%, SI Figure S1A).

αS_1–76_-MES (**1a**) and αS_77–84_-Pen_77_^Ac^K_80_-NHNH_2_ (**2**) were ligated overnight under routine NCL conditions (NCL1) in the presence of 4-mercaptophenylacetic acid (MPAA). (Yield: 1.46 mg, 172 nmol, 57%, Figure 2B). The purified product (**3a**) was activated by oxidation to form a MES thioester (**3b**) (Yield: 1.29 mg, 126 nmol, 73%, SI Figure S1C). The second ligation (NCL2) between αS_1–84_-Pen_77_^Ac^K_80_-MES (**3b**) and αS_85–140_-C_85_ (**4**) to form αS-Pen_77_C_85_^Ac^K_80_ (**5a**) as performed in the presence of methyl thioglycolate to allow for desulfurization without intermediate purification (Figure 2C).(Huang et al., 2016) The product, αS-^Ac^K_80_ (**5b**), was obtained in 43% yield (0.90 mg, 62 nmol, Figure 2D). Although we successfully completed this synthesis, it is notable that we encountered solubility issues of the intermediate fragments (**3b**, **5a**) and the product (**5b**), after lyophilization.

**Figure 3.**
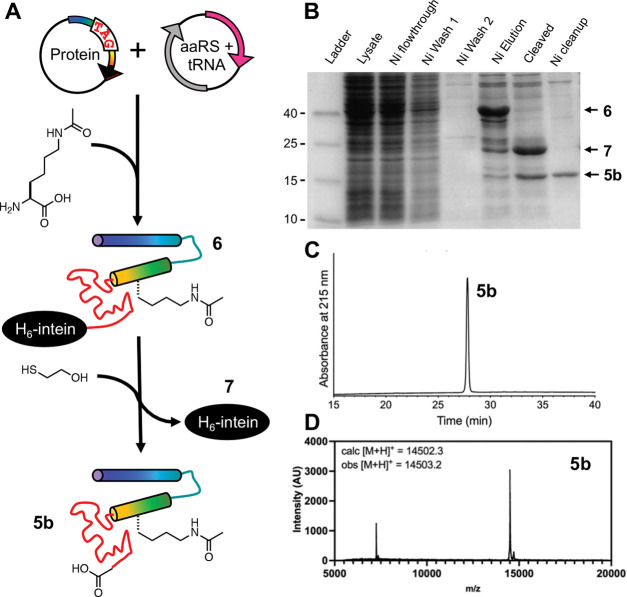
Expression of αS-^Ac^K_80_ through ncAA mutagenesis. (A) An orthogonal aminoacyl tRNA synthetase (aaRS)/tRNA pair site-specifically incorporates acetyllysine in recombinant αS. Intein tagging at the C-terminus allows for traceless purification of the full-length product. (B) SDS-PAGE gel (Coomassie stain) showing Ni-affinity purification of recombinant αS-^Ac^K_80_. Purified αS-^Ac^K_80_ (**5b**) characterized with (C) analytical HPLC and (D) MALDI MS.

Experiencing difficulties in sample handling and considering the inefficiency of applying NCL to scan 12 lysine acetylation sites distributed throughout the protein, we then sought to access site-specifically acetylated αS through ncAA mutagenesis (Figure 3A). We recombinantly expressed αS with lysine acetylation at site 80 in *E. coli* through amber codon suppression. We used a previously reported pair of aminoacyl tRNA synthetase (chAcK3RS with IPYE mutations) and cognate tRNA to incorporate *ε*-acetyllysine at a position dictated by an amber stop (TAG) codon(Bryson et al., 2017). In addition to 10 mM *ε*-acetyllysine, 50 mM nicotinamide, an inhibitor to endogenous deacetylases, was added to the media before inducing αS expression. The protein was expressed as an intein fusion as reported before for easy removal of truncated protein through affinity purification.(Batjargal et al., 2015) After traceless intein cleavage with 2-mercaptoethanol, the ^Ac^K-containing protein was purified by reverse phase high performance liquid chromatography (RP-HPLC) and exchanged into appropriate buffers for biophysical assays (Figure 3B).

We obtained 0.65 mg of pure αS-^Ac^K_80_ per L of bacterial culture (Figure 3C,3D), a yield comparable to that obtained by NCL, but because no lyophilization or handling of ligation intermediates is required, we did not encounter the solubility problems observed in the NCL process. Therefore, we deemed the ncAA mutagenesis approach at least comparable to NCL for producing a specific construct. Since we wished to study 12 sites distributed throughout the αS sequence, ncAA mutagenesis was also advantageous because we avoided having to generate constructs for several different ligation sites and could simply perform site-directed mutagenesis to insert TAG codons for each new αS-^Ac^K_n_ variant.

Bolstered by our success with ^Ac^K_80_, we generated TAG mutants at sites 12, 21, 23, 32, 34, 43, 45, 58, 60, 96, or 102. We expressed and purified these proteins, observing successful ncAA mutagenesis at each site (Figs. S2–S12), however, the yield varied significantly between different sites (0.11–1.5 mg). This is an interesting result in light of the large number of sites that were tested in the same protein and the fact that αS is an intrinsically disordered protein, so protein folding should not affect incorporation. Examination of the local RNA sequence context of the amber (TAG) codon did not explain the varied suppression efficiency, based either on previously identified sequence impacts (Pott et al., 2014) or by comparing the sites within αS (Figure S13). Given the pseudo-repeat nature of the αS sequence, many sites feature similar sequences, and a comparison of 21 and 58 is particularly striking with a 10-fold difference in expression levels despite near identity in the flanking sequences. While these observations are notable for users of ncAA technology, in the context of this study, our approach allowed us to acquire sufficient amounts of the 12 different authentically modified αS constructs for biophysical experiments.

Thus, in spite of low expression yields for some sites, ncAA mutagenesis was a preferred method for this work, due to the better efficiency in scanning 12 different modification sites and the ease of handling aggregation-prone protein fragments. The expression-based strategy also allows for low-cost access to isotopically labeled, PTM-modified αS constructs, as we have demonstrated previously.**(Pan et al.)**

### Effects on αS Helicity on Micelles and Aggregation

αS is known to bind to lipid surfaces and form helical structures, part of its physiological role in modulating neurotransmitter vesicle trafficking(Ramirez et al., 2023). More specifically, on micelles, an NMR structure showed that micelle-bound αS forms a broken helix, where two helical strands are connected with a loop region (Figure 1B, PDB: 1xq8)(Ulmer et al., 2005). A helical wheel model, created based on this structure, shows that lysine residues are aligned on the membrane surface, and that they are likely involved in enhancing binding by interactions with negatively charged lipid head groups(Meade et al., 2019).

**Figure 4.**
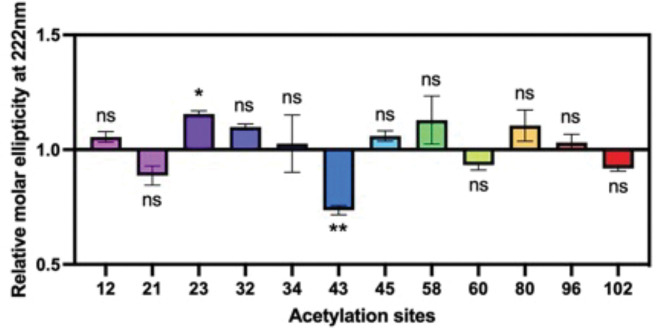
Effects of lysine acetylation on micelle-bound αS. Molar ellipticity at 222 nm was normalized to WT value to quantify helicity on SDS micelles. Mean with SD, R=3

With each acetylated αS variant, we first examined the effects of Lys acetylation on the secondary structure of αS in the presence of micelles by wavelength scan circular dichroism (CD) spectroscopy. Each acetylated αS was compared to unmodified wild type (WT) αS in phosphate-based buffer, pH 7.4, with a large excess of sodium dodecyl sulfate (SDS). We normalized the molar ellipticity at 222 nm of each acetylated construct to that of WT to compare the effect on helicity at each site. We found that significant reduction of helicity was caused only by acetylation at site 43 and that acetylation at other sites had only minor effects on helicity (Figure 4, Figure S14). This study implies that only this site could potentially perturb αS function in neurotransmitter trafficking.

We then investigated whether lysine acetylation at different sites has impacts on αS in pathological contexts. To do this, we first performed *in vitro* aggregation experiments and assessed site-specific effects. A plate-based approach was taken to efficiently perform the assay, and each aggregation reaction was seeded by mixing with αS WT pre-formed fibrils (PFFs) that constituted 10% of the total monomer concentration. The monomer samples were prepared by mixing αS WT with acetylated αS at either 10% or 25% of the total monomer concentration. These concentrations were chosen because our quantitative studies of other PTMs in patient samples indicated that most were present in this range, rather than stoichiometrically(Zhang et al., 2023; Zhao et al., 2022) (see additional discussion in Conclusions). Aggregation was carried out at 37 °C with shaking and kinetics and thermodynamics (final fibril amounts) were monitored.

**Figure 5.**
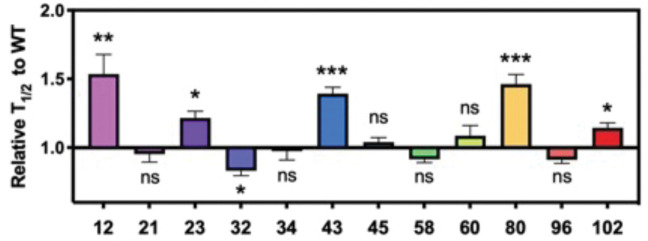
Effects of lysine acetylation on *in vitro* aggregation. Aggregation kinetics were monitored by fluorescence intensity change of ThT. Time it takes to reach 50% fibrilization (T_1/2_) for each condition was normalized to that of WT. Seeded aggregation was performed with αS monomers where acetylated αS was mixed with αS WT at 25%:75% ratio. SEM, R=6

To examine the effects on aggregation kinetics, we took advantage of the change in fluorescence of the amyloid binding dye, thioflavin T (ThT), during aggregation to monitor the process *in situ*. We found that the effects differ between different modification sites (Figure 5, Figure S15–16). For Lys acetylation at 12, 23, 43, 80 and 102 we observed differential slowing effects – the effects were particularly significant at sites 12, 43 and 80, and the effects at 12, 23 and 43 were dose dependent. While we observed acceleration of aggregation for site 32 both at 10% and 25%, the effects were similar between the different dosages.

To confirm that these effects were not the result of reduced monomer incorporation, we isolated fibrils at the endpoint of 10% or 25% aggregations and SDS-PAGE gels were run and stained with the Coomassie Brilliant Blue dye to quantify total monomer incorporation into the fibrils. We found that there were no consistent reductions in monomer incorporation, and in fact there were some moderate apparent enhancements of incorporation. However, these were generally not consistent between the 10% and 25% aggregation experiments, except in the case of ^Ac^K_34_ (Figure S17–18). Taking all of the aggregation kinetics and monomer incorporation data into account, we chose to investigate the kinetically perturbed sites 12, 43, and 80 further, since the cellular process will be unlikely to reach equilibrium and our previous study had shown that the Gln mimic mutation at position 34 did not alter aggregation in cells.(Zhang et al., 2023)

### Fibril Seeding in Neurons

**Figure 6.**
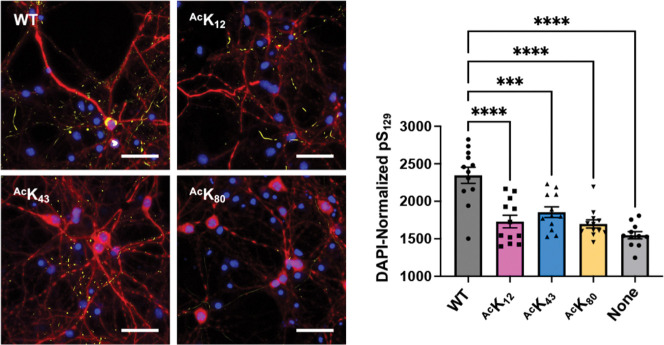
Effects on aggregation seeding in primary neuron cells. Left: representative images of neuron cultures treated with unmodified or 25% acetylated αS PFFs, stained with an anti-pS_129_ antibody (yellow), DAPI (blue), and an anti-MAP2 antbody (red). Scale bar = 50 μm. Right: quantification of DAPI-normalized anti-pS_129_ area of intracellular aggregates seeded by different αS PFFs. Mean with SE, R= 11–12. *** = 0.001 < p-value < 0.0001; **** = 0.00001 < p-value < 0.0001

To investigate the impact of Lys acetylation on aggregation in more physiologically relevant contexts – in cultured neurons – we followed the approach that we have done previously with arginylated αS.(Pan et al., 2022; Zhao et al., 2022) We prepared PFFs with the following compositions: αS WT or αS WT mixed with 25% acetylated αS, ^Ac^K_12_, ^Ac^K_43_ or ^Ac^K_80_. Mouse primary hippocampal neurons were grown for 8 days on a coated plate, to which 50 ng/μL PFFs were added, following established protocols.(Haney et al., 2016; Luk et al., 2009; Marotta et al., 2021) After 2 weeks, intracellular αS aggregates were quantified by staining with an antibody that recognizes phosphoserine 129 (pS_129_), a commonly used pathological marker (Figure 6, Figure S19). Compared to the WT PFFs, all the acetylated PFFs tested resulted in significantly reduced aggregation seeding: the pS_129_ signal (AU ± SEM (arbitrary units, standard error of the mean)) of PFF-seeded αS aggregates was 2347 ± 107.1 (WT), 1730 ± 83.67 (^Ac^K_12_), 1854 ± 70.79 (^Ac^K_43_), 1698 ± 54.41 (^Ac^K_80_) with respect to DAPI. Notably, acetylation at these sites also slowed seeded aggregation in the *in vitro* fibrilization experiment, but did not reduce aggregates quantified at the endpoint. This supports the idea that aggregation in a cellular context is unlikely to reach saturation.

### Structural Characterization of αS Monomers

Having demonstrated that acetylation at K_12_, K_43_ or K_80_ significantly reduced αS aggregation *in vitro* and in cells, we wished to gain information on the structural impact of acetylation at these sites. First, to give insights into the effects on monomer conformation, we acquired proton-nitrogen correlation spectra (^1^H,^15^N – HSQC) for ^Ac^K_12_, ^Ac^K_43,_ or ^Ac^K_80_, an experiment that is facile with ncAA mutagenesis, but challenging to perform via NCL due to the high cost of isotopically-labeled amino acids for SPPS. To access ^15^N-labeled αS, we expressed the acetylated αS and αS WT in M9 minimal media containing ^15^N-labeled ammonium chloride (SI Figures S20–22). This afforded comparable protein yields to expressions in LB media. It is notable, however, that sub-stoichiometric isotopic labeling was observed at some Lys sites, depending on batches of expression, which could be due to deacetylation in *E. coli* cells followed by incorporation at Lys codons. Overlaying the HSQC spectra for αS-WT and αS- ^Ac^K_12_, ^Ac^K_43_ or ^Ac^K_80_, peak shifts were observed only in signals from surrounding residues, suggesting that the structural change was local and there is no major impact of lysine acetylation on monomer structure (SI Figures S23–S24).

### Biophysical Characterization of Lipid Binding

We next wished to learn the effects of Lys acetylation at K_12_, K_43_ or K_80_ on the native function of αS by investigating its lipid binding mode. To quantify conformational changes of αS upon vesicle binding, we acquired ^1^H,^15^N – HSQC spectra for WT, ^Ac^K_12_, ^Ac^K_43_ or ^Ac^K_80_ in the presence of small, unilamellar vesicles (SUVs) that are composed of 60:25:15 1,2-dioleoyl-sn-glycero-3-phosphocholine/1,2-dioleoyl-sn-glycero-3-phosphatidylethanolamine/1,2-dioleoyl-sn-glycero-3-phospho-L-serine (DOPC/DOPE/DOPS). The NMR peak chemical shifts were similar for all constructs and consistent with spectra previously reported for WT αS(Eliezer et al., 2001). There was no notable chemical shift perturbation at the surrounding residues of each acetylation site (Figure S25).

**Figure 7.**
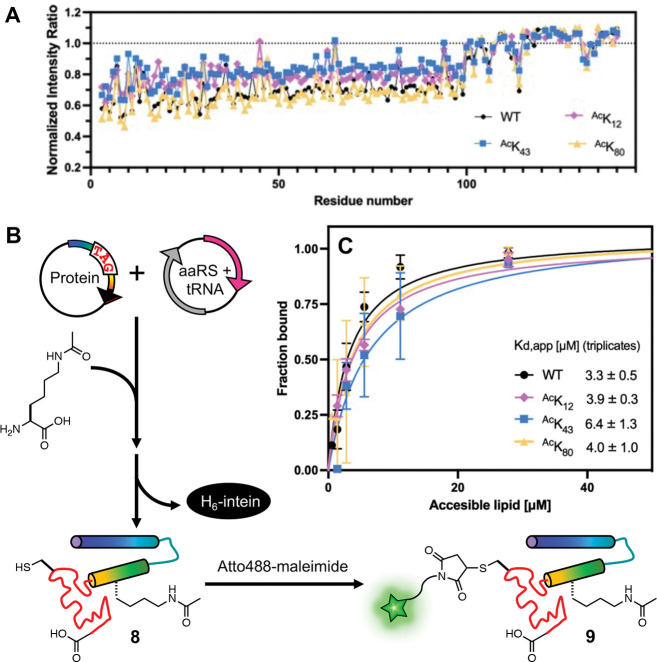
Effects of lysine acetylation on vesicle binding affinity. (A) NMR intensity ratio for each residue calculated from ^1^H-^15^N HSQC spectra collected with ^15^N-labeled αS variants in the presence or absence of SUVs, normalized by the average ratio for residues 101–140. (B) αS with a TAG codon at the acetylation site of interest and a Cys mutation at a labeling site (**8**) was co-expressed with an aaRS/tRNA plasmid for acetyllysine incorporation. After intein cleavage, labeling with an Atto488 dye was performed through Cys-maleimide chemistry to give an acetylated, labeled protein (**9**) for FCS. (C) Vesicle binding affinity determined by fluorescent correlation spectroscopy measurements. For each construct, measurements were performed on three separate days. Mean with SD, R=3

In the presence of vesicles, a reduction of intensity for residues 1–100 was observed for all the constructs, which is caused by binding of this portion of αS to the slowly tumbling lipid vesicles and is again consistent with previous observations(Bodner et al., 2009; Bussell and Eliezer, 2004). αS- ^Ac^K_80_ had a similar intensity change to WT (~40%, Figure 7A, Figure S26), whereas αS-^Ac^K_43_ had a smaller intensity change, suggesting weaker vesicle binding (~20%, Figure 7A, Figure S26). This is consistent with the acetylation effects observed with SDS micelles (Figure 4). αS-^Ac^K_12_ had an intermediate intensity reduction (~30%, Figure 7A, Figure S26), a more significant effect of K_12_ acetylation on vesicle binding than what was observed with SDS micelles (Figure 4). It is possible that this is due to the differences in curvature and headgroup between the micelles and the vesicles, which is known to result in different αS binding modes(Cholak et al., 2020; Georgieva et al., 2010; Middleton and Rhoades, 2010; Rhoades et al., 2006; Trexler and Rhoades, 2009). It is also possible that this is due to the increased sensitivity of NMR to subtle differences in binding.

While NMR is a very valuable technique for characterizing vesicle binding with a non-perturbing label, to measure affinity, we turned to FCS, a well-established method for rigorously determining vesicle apparent dissociation constants (K_d,app_) (Rhoades et al., 2006). To enable this experiment, we expressed the acetylated αS constructs at site 12, 43, or 80 or a non-acetylated construct (“WT”), bearing a Cys mutation at site 114 (**8**, αS–^Ac^K_12_C_114_, αS–^Ac^K_43_C_114_, αS–^Ac^K_80_C_114,_ and αS–C_114_) to allow for fluorescent labeling (Figure 7B, SI Figures S27–30). The fluorophore Atto488-maleimide was reacted with purified Cys mutants overnight at 4 °C or for a few hours at room temperature to yield labeled constructs (**9**, αS–^Ac^K_12_C^Atto488^_114_, αS–^Ac^K_43_C^Atto488^_114_, αS–^Ac^K_80_C ^Atto488^_114_, and αS–C ^Atto488^_114_), and the conversion was almost quantitative (SI Figures S27–30). We prepared synthetic lipid vesicles containing 50:50 1-palmitoyl-2-oleoyl-sn-glycero-3-phospho-L-serine/1-palmitoyl-2-oleoyl-glycero-3-phosphocholine (POPS/POPC). The diffusion times of free αS and of the vesicles were obtained first, and in assessing the αS-vesicle binding, we added the same quantity of αS to varied concentrations of vesicles and then determined the protein fractions bound by fitting a two-component autocorrelation function. The fraction bound values at each vesicle concentration were used to fit a binding curve for each αS construct. We found that acetylation at site 43 leads to two-fold weaker binding and acetylation at site 12 or 80 did not significantly affect binding (Figure 7C, SI Figures S31–32; K_d,app_^WT^ = 3.2 ± 0.5 μM, K_d,app_^AcK12^ = 3.9 ± 0.3 μM, K_d,app_^AcK43^ = 6.3 ± 1.3 μM, K_d,app_^AcK80^ = 4.0 ± 1.0 μM).

Slightly reduced binding due to acetylation at site 43 correlates with the reduced helicity we observed in the CD wavelength scan and the differences in NMR peak intensities in the presence of vesicles. The NMR experiments showed that ^Ac^K_43_ reduced vesicle binding more significantly than ^Ac^K_12_ (moderate) or ^Ac^K_80_ (little to none). The FCS experiments supported this, showing that ^Ac^K_43_ led to weaker binding than ^Ac^K_12_ or ^Ac^K_80_, which were similar to WT. Previous NMR experiments suggested that the *N*-terminal helix of αS (residues 6–25) drives association with lipid membranes and the 26–97 region modulates the affinity, depending on lipid composition(Fusco et al., 2014). The different effect between ^Ac^K_43_ and ^Ac^K_12_ or ^Ac^K_80_ suggests that K_43_ is more important in modulating the binding affinity.

Taken together, our results show that among all the disease-relevant acetylation sites, ^Ac^K_12_, ^Ac^K_43_, and ^Ac^K_80_ each inhibit aggregation, but that ^Ac^K_43_ also inhibits membrane binding (as does ^Ac^K_12_, to a lesser degree). Thus, in the case of ^Ac^K_43_, the potential benefits of reduced amyloidogenicity may be offset by compromising function in neurotransmitter release.

### Structural Characterization of αS Fibrils

**Figure 8.**
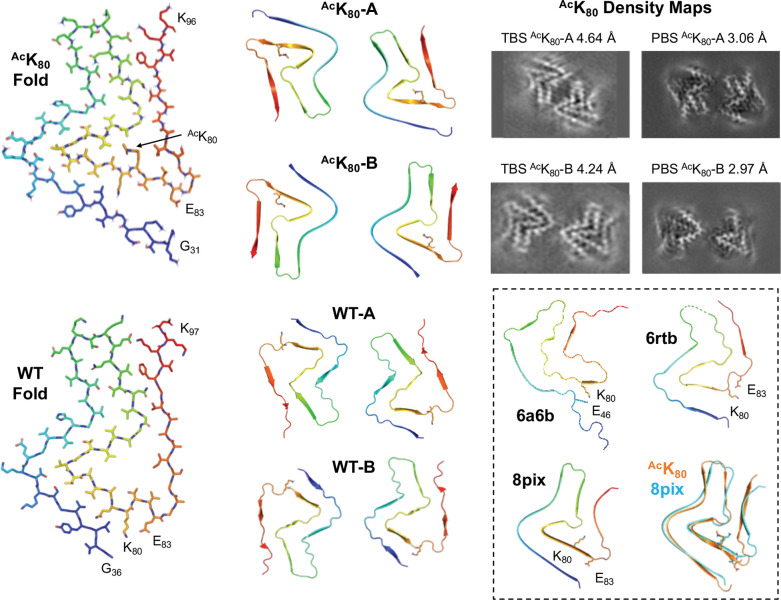
Structural impact of K_80_ acetylation on fibril morphology. ^Ac^K_80_ Fold and WT Fold show the fold of a single αS molecule in the fibrils, viewed down the fibril axis (from ^Ac^K_80_-A PBS and WT-A TBS structures). ^Ac^K_80_-A and ^Ac^K_80_-B show the two fibril polymorphs, with similar protein folds, but different strand-strand packing (from PBS structures). WT-A and WT-B show the two fibril polymorphs, with similar protein folds, but different strand-strand packing (from TBS structures). ^Ac^K_80_-A Density Maps show that the same fibril polymorphs were obtained for fibrils made in TBS and PBS. Inset: The interactions of K_80_ are shown in three previously αS fibril polymorphs designated by their PDB IDs.(Frey et al., 2024; Guerrero-Ferreira et al., 2018; Li et al., 2018b) The overlay shows the similarity of the ^Ac^K_80_ fold to the 8pix fold.

To get preliminary insights into fibril structure effects, we performed TEM imaging on fibrils formed from acetylated αS (αS-^Ac^K_12_, ^Ac^K_43_ or ^Ac^K_80_), mixed with αS WT, at 25% of the total monomer concentration (SI Figure S33). Interestingly, we observed mixed morphology for PFFs prepared with αS-^Ac^K_12_, with some very narrow fibrils. Both 25% ^Ac^K_12_ and 25% ^Ac^K_43_ PFFs have minimal helical twist, making them difficult to characterize by cryo-EM. On the other hand, for PFFs prepared with 25% αS-^Ac^K_80_, we observed a slightly more twisted fibril morphology, so we attempted to solve a structure by single particle cryo-EM methods. We were able to solve a structure to 2.88 Å resolution, and comparison to previously published αS WT fibril structures (see SI for experimental details, density images, and atomic models, SI Figure S34) shows that the backbone fold is similar to the two-stranded polymorph typified by PDB ID 6a6b (Figure 8, inset, 6a6b),(Li et al., 2018b) similar to PDB IDs 6cu7(Li et al., 2018a) and 6h6b(Guerrero-Ferreira et al., 2018), which all have the “Greek key” protein fold first reported in solid state NMR studies of single stranded fibrils under PDB ID 2n0a.(Tuttle et al., 2016) However, the density clearly shows that K_80_ is not acetylated in this structure, implying that the WT fibrils are forming this 6a6b-type fibril, while the ^Ac^K_80_ fibrils are forming a separate, minority population that we are not able to resolve.

In order to more clearly observe the structural impact of K_80_ acetylation, we prepared fibrils with 100% ^Ac^K_80_ αS in Tris-buffered saline (TBS) for cryo-EM studies. For these fibrils, we were able to solve structures of two different polymorphs, both composed of two strands (Figure 8, ^Ac^K_80_-A and ^Ac^K_80_-B, see SI for experimental details and density images, and atomic models, SI Figures S35, S37, S38). The protein fold is essentially the same in both polymorphs, but they differ in strand-strand packing. Since it is well-documented that differences in buffer composition and aggregation methods can lead to differences in fibril morphology, we also prepared ^Ac^K_80_ αS fibrils in phosphate-buffered saline (PBS), the same conditions used in our aggregation kinetics studies. Gratifyingly, the ^Ac^K_80_ αS fibrils prepared in PBS exhibited the same two polymorphs seen for TBS ^Ac^K_80_ αS fibrils, with identical protein folds and strand-strand packings (Figure 8, and SI Figure S39). We were also able to solve cryo-EM structures of WT αS fibrils generated under the same conditions in TBS. We observed two WT polymorphs (Figure 8, WT-A and WT-B, see SI for experimental details and density images, and atomic models, SI Figures S35, S36, S38) which exhibited similar folds and strand-strand packings to the ^Ac^K_80_ polymorphs, but with a notable change in morphology around K_80_. Acetylation of K_80_ disrupts a salt-bridge interaction with E_83_ that can be clearly seen in the WT-B polymorph (Figure 8, WT Fold) and neutralizes the sidechain charge, allowing it to pack in a hydrophobic pocket formed by Ala_69_ and Val_71_ (Figure 8, ^Ac^K_80_ Fold). This leads to a twist of the backbone in the T_75_-A_90_ segment, generating a modest change in the protein fold. Given that the ^Ac^K_80_ protein fold is fairly similar to the WT protein fold, it is not surprising that they exhibit similar strand-strand packings and that K_80_ acetylation has a moderate impact on aggregation rates. We can use these structures to consider ^Ac^K_80_ effects in the context of other structural studies of αS fibrils.

Our WT-A and WT-B structures resemble those first reported under PDB IDs 6rtb and 6rto (Figure 8, inset, 6rtb; see SI for additional overlays and structural comparisons, Figure S40).(Guerrero-Ferreira et al., 2019) These polymorphs have been observed by several investigators for WT αS fibrils formed at near-neutral pH, along with the commonly observed 6a6b/2n0a form (Figure 8, inset, 6a6b).(Li et al., 2018b) Our ^Ac^K_80_ αS fibril structures, ^Ac^K_80_-A and ^Ac^K_80_-B, resemble those recently reported for WT αS fibrils formed at pH ≤6.5 under PDB IDs 8pix and 8pic (Figure 8, inset, 8pix).(Frey et al., 2024) In the two fibril polymorphs commonly populated at pH 7, K_80_ makes key stabilizing salt bridge interactions. For the 6a6b polymorph, K_80_ makes a salt bridge with E_46_; for the 6rtb polymorph, it makes a salt bridge with E_83_. Disruption of these salt bridges by acetylation would destabilize either fold, favoring the ^Ac^K_80_ fold that we observe. The fact that a similar fold (8pix) has been seen at lower pHs can be rationalized by assuming that acidification leads to protonation of the E_46_ or E_83_ sidechains, weakening their interactions with K_80_ just as acetylation does to drive a change fibril polymorph. While pH 6.5 is significantly above the pK_a_ of a typical glutamate sidechain, it is possible that the pK_a_s are perturbed in the local environment of the fibril, and full deprotonation would not be required to destabilize interactions with K_80_. Thus, one can rationalize our observation of a polymorph like 8pix for our ^Ac^K_80_ at pH 7, when it had only been previously observed at lower pHs. It is further notable that the ^Ac^K_80_ fold is robust to changes in buffer (PBS vs. TBS).

Finally, it is worth comparing the effects that we observe for ^Ac^K_80_ acetylation to the structural and biophysical effects observed with other αS PTMs which have been structurally characterized, such as Y_39_ phosphorylation (pY_39_) or S_87_ phosphorylation (pS_87_) and *N*-Acetyl glucosamine glycosylation (gS_87_). For pY_39_ αS, a 4-fold increase in aggregation rate has been observed for αS 100% phosphorylation, with nuanced effects at lower phosphorylation percentages. We have shown that pY_39_ leads to only modest changes in monomer conformation, based on NMR and single molecule FRET studies.(Pan et al.; Pan et al., 2020b) In contrast, Zhao *et al*’s cryo-EM structure shows a dramatic rearrangement of the fibril polymorph.(Zhao et al., 2020) This indicates that the effect of Y_39_ phosphorylation is primarily at the fibril level, similar to our findings here for ^Ac^K_80_ acetylation. Both pS_87_ and gS_87_ modifications inhibit aggregation much more drastically than pY_39_. Two different structures of gS_87_ αS fibrils have been reported, both showing a significant deviation from reported WT αS polymorphs.(Balana et al., 2024; Hu et al., 2024) Despite their differences, the structures both provide a clear rationale for the effects of S_87_ glycosylation. Intriguingly, Hu *et al*. highlight the way in which gS_87_ changes the structure of the 80–89 region of αS to disrupt the E_46_-K_80_, destabilizing the 6a6b fold.(Hu et al., 2024) The pS_87_ structure is different from either gS_87_ structure, and although the residue cannot be observed in the structure, it again demonstrates that a PTM can dramatically alter fibril morphology. Thus, like ^Ac^K_80_ studied here, these PTMs seem to primarily exert their influence on αS aggregation through changes in fibril structure, which is sensible given the disordered nature of the αS monomer.

### Deacetylase Site Specificity

**Figure 9.**
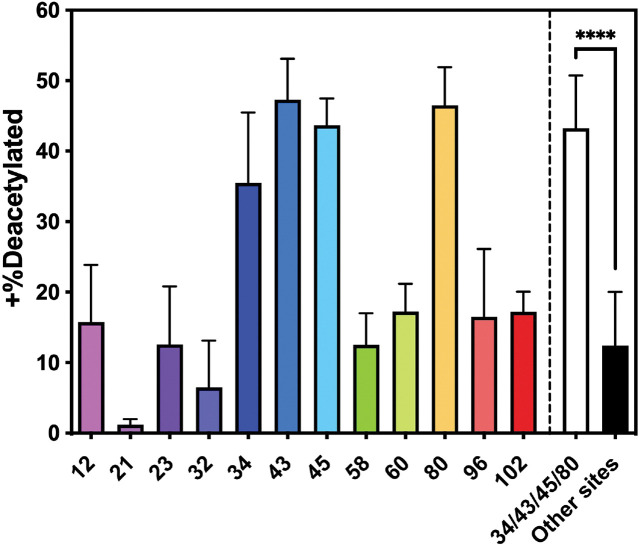
Site-specificity of HDAC activity. Samples of each of the acetylated αS variants were mixed with HDAC8 and after 24 h, acetylation levels were checked with a MALDI MS assay using ^15^N-labeled αS as a standard. Mean with SD, R=3

Since acetylation of K_12,_ K_43_ and K_80_ can reduce αS aggregation, we considered the potential of increasing acetylation at these sites by inhibiting a KDAC. Doing so would require that the KDAC had some specificity for these residues. Using previously published methods,(Decroos et al., 2014; Dowling et al., 2008; Osko et al., 2021) we expressed and purified recombinant human HDAC8, a Zn-dependent HDAC known to act on non-histone proteins, including cytosolic targets like tubulin in HeLa cells(Vanaja et al., 2018). We treated samples of each of the acetylated αS variants with HDAC8 and monitored deacetylation through a matric assisted laser desorption ionization (MALDI) MS assay using ^15^N-labeled αS as a standard. After 24 h, all of the constructs showed significant levels of deacetylation, but ^Ac^K_34_, ^Ac^K_43_, ^Ac^K_45_, and ^Ac^K_80_ retained an average of 44% acetylation, 3-fold greater levels than all other sites (Figure 9). These preliminary results indicate that inhibition of HDAC8 could increase acetylation levels of αS at specific lysine residues shown to retard aggregation *in vitro* and in PFF-seeded neurons.

## Conclusion

In this study, we incorporated ^Ac^K site-specifically at all 12 disease-relevant sites through ncAA mutagenesis and characterized the effects of this PTM on the physiological and pathological roles of αS using a variety of techniques. The aggregation assays showed that many of the Lys acetylations observed in patient samples have no effect, demonstrating that there is no non-specific effect on protein solubility or electrostatic interactions, at least for single Lys modifications. At sites 12, 43, and 80, Lys acetylation significantly slowed the formation of fibrils, both *in vitro* and in cells. Therefore, increasing acetylation at these sites through the use of KAT stimulators or KDAC inhibitors has potential therapeutic benefits. However, acetylation at Lys 12 or 43 perturbs membrane binding moderately, so increasing acetylation at these sites in αS could disrupt its native function in neurotransmitter vesicle trafficking. Thus, Lys 80 seems like the most promising site for targeted acetylation. Indeed, our previous cell-based studies using Gln mimics have shown that Lys 80 modification reduces aggregation.(Zhang et al., 2023) By determining cryo-EM structures of ^Ac^K_80_ fibrils, we have provided a structural explanation for its inhibitory effects. Collectively, our results imply that strategies that can specifically enhance acetylation at Lys 80, without affecting Lys 12 or Lys 43, would be the most favorable approach to reduce αS aggregation pathology.

It should be noted that it is not clear at this point exactly what the acetylation levels at K_12_, K_43_ and K_80_ are in synucleinopathy patients. Taking advantage of our capability to produce authentically acetylated αS, we determined the extent of acetylation within human protein samples by quantitative liquid chromatography MS (SI, Figures S41-S49). The ^Ac^K αS standards allowed us to correct for changes in trypsinization and ionization efficiency of acetylated peptides, the latter of which turned out to be very low for the ^Ac^K_80_ peptide due to its large size (a result of the missed K_80_ cut site due to acetylation, SI Table S5). The level of acetylation was variable – no clear trend was observed between healthy control and patients – nor between patients of different diseases (SI, Table S4, Supplementary Data 1). Nevertheless, the MS data suggest that the 10 and 25% acetylation that we used for aggregation experiments are in the (patho)physiological range. Given the results reported here, it will be valuable to generate antibodies to acetylated peptides for the ^Ac^K_12_, ^Ac^K_43_ and ^Ac^K_80_ epitopes to more easily quantify the levels of acetylation in both soluble and fibrillar αS for immunofluorescence microscopy and Western blotting studies.

More broadly, our experiments show the value of a ncAA mutagenesis approach in systematically investigating a PTM that occurs at many locations in a protein. Since the yields were similar between NCL and ncAA mutagenesis, the ability to scan many sites by simple site-directed mutation to a TAG codon clearly makes ncAA mutagenesis the method of choice for our application. We efficiently scanned 12 different modification sites and fluorescently labeled proteins for binding studies. The ncAA approach was also crucial to generating isotopically labeled, acetylated αS for solution-phase NMR experiments and MS analysis. The isotopic labeling approach could be used in future solid-state NMR experiments to give detailed structural insight into slowed aggregation and distinct fibril morphology.

Our future experiments will include assessing the site-specificity of KATs and other KDACs for sites in αS in a similar fashion to the HDAC8 experiments here, studies enabled by our ability to easily produce ^Ac^K αS constructs. We can study modulation of HDAC8 and these other enzymes for their ability to specifically increase acetylation at Lys 80 without altering acetylation at Lys 12 or 43. We will also investigate effects on αS aggregation in cellular models in the presence of small molecule modulators of HDAC8 and these other enzymes. Furthermore, combining ncAA mutagenesis and NCL would allow us to study more complex PTM effects in αS, such as the combinatorial effects between multiple lysine acetylations or crosstalk between acetylation and other PTMs.

## Supplementary Material

Supplement 1

Detailed protocols for plasmid mutagenesis, protein semi-synthesis and ncAA mutagenesis, fluorescent labeling, CD, aggregation measurements, neuron seeding, TEM, lipid vesicle preparation, FCS measurements, NMR data acquisition, cryo-EM structure determination, and MS analysis as well as additional figures (PDF and spreadsheet) are provided in Supporting Information. The authors have cited additional references within the Supporting Information.(Anderson, 1946; Delaglio et al., 1995; Dikiy and Eliezer, 2014; Lee et al., 2015; Maciejewski et al., 2017; Volpicelli-Daley et al., 2011; Waxman and Giasson, 2008)
