# Supplementary material for: Investigation of All Disease-Relevant Lysine Acetylation Sites in α-Synuclein Enabled by Non-canonical Amino Acid Mutagenesis
Source: bioRxiv. 2025 Aug 22:2025.01.21.634178. Preprint. [Version 3] doi: 10.1101/2025.01.21.634178 (PMC11785115; doi:10.1101/2025.01.21.634178)
Supplement: Supplement 1 [file media-1.pdf]

Supporting Information for

Investigation of All Disease-Relevant Lysine Acetylation Sites in α-Synuclein Enabled by Non-canonical Amino Acid Mutagenesis

Marie Shimogawa,[1] Grace Shin Hye Park,[2] Jennifer Ramirez,[2] Hudson Lee,[3] Paris R. Watson,[1] Swati Sharma,[1] Zongtao Lin,[4] Chao Peng,[5] Virginia M.-Y. Lee,[6] Benjamin A. Garcia,[4] David W. Christianson,[1] David Eliezer,[3] Elizabeth Rhoades,[1,7] and E. James Petersson[1,7]*

[1]*Department of Chemistry; University of Pennsylvania; 231 South 34th Street; Philadelphia, PA 19104, USA.*

[2]*Graduate Group in Biochemistry and Molecular Biophysics, Perelman School of Medicine, University of Pennsylvania, 206 Anatomy-Chemistry Building, 3620 Hamilton Walk, Philadelphia, PA 19104, USA.*

[3]*Department of Biochemistry, Weill Cornell Medicine, 1300 York Avenue; New York, NY, 10065, USA.*

[4]*Department of Biochemistry and Molecular Biophysics, Washington University St Louis, St Louis, MO 63130, USA*

[5]*Department of Neurology, David Geffen School of Medicine, University of California - Los Angeles, Los Angeles, California 90095, USA*

[6]*Department of Pathology and Laboratory Medicine, Center for Neurodegenerative Disease Research, University of Pennsylvania, 3600 Spruce Street, Philadelphia, PA 19104, USA*

[7]*Department of Biochemistry and Biophysics, Perelman School of Medicine, University of Pennsylvania, 421 Curie Boulevard, Philadelphia, PA 19104, USA.*

*email: ejpetersson@sas.upenn.edu

Contents:

List of Figures, Schemes, and Tables **S2**
General information **S4**
Protein semi-synthesis for generation of αS -$^{Ac}K_{80}$ **S5**
Construction of expression plasmids **S5**
Production of recombinant αS constructs **S6**
Fluorescent labeling **S7**
Circular dichroism (CD) **S7**
Protein aggregation kinetics and percentage incorporation into fibrils **S8**
In situ aggregation in cultured primary hippocampal neurons **S9**
Preparation of synthetic lipid vesicles **S9**
Heteronuclear single quantum coherence spectroscopy (HSQC) **S10**
Fluorescence correlation spectroscopy (FCS) **S10**
Vesicle binding affinity **S11**
Transmission electron microscopy (TEM) **S12**
Cryo-electron microscopy (Cryo-EM) data acquisition **S13**
Cryo-EM data processing **S13**
Model building and refinement **S14**
Preparation of human HDAC8 .... **S14**
HDAC8 deacetylation assay **S15**
Proteomic analysis of acetylated αS standards **S16**

Quantitative analysis of αS Lys acetylation in human samples **S17**
Schemes **S19**
Figures and Tables **S20**
References **S67**

Figures:

Figure S1. Protein semisynthesis to incorporate acetyllysine at position 80 .... **S20**
Figure S2. ncAA mutagenesis to incorporate acetyllysine at position 12 .... **S21**
Figure S3. ncAA mutagenesis to incorporate acetyllysine at position 21 .... **S21**
Figure S4. ncAA mutagenesis to incorporate acetyllysine at position 23 .... **S22**
Figure S5. ncAA mutagenesis to incorporate acetyllysine at position 32 .... **S22**
Figure S6. ncAA mutagenesis to incorporate acetyllysine at position 34 .... **S23**
Figure S7. ncAA mutagenesis to incorporate acetyllysine at position 43 .... **S23**
Figure S8. ncAA mutagenesis to incorporate acetyllysine at position 45 .... **S24**
Figure S9. ncAA mutagenesis to incorporate acetyllysine at position 58 .... **S24**
Figure S10. ncAA mutagenesis to incorporate acetyllysine at position 60 .... **S25**
Figure S11. ncAA mutagenesis to incorporate acetyllysine at position 96 .... **S25**
Figure S12. ncAA mutagenesis to incorporate acetyllysine at position 102 .... **S26**
Figure S13. Local sequential contexts of amber codon suppression **S26**
Figure S14. Individual CD wavelength scans for αS-^{Ac}K constructs **S27**
Figure S15. Aggregation kinetics curves for each αS-^{Ac}K construct **S29**
Figure S16. Effects of 10% αS-^{Ac}K on aggregation kinetics **S31**
Figure S17. Primary SDS-PAGE gels for quantifying monomer incorporations of αS-^{Ac}K constructs ... **S32**
Figure S18. Effects of αS-^{Ac}K on total monomer incorporation **S35**
Figure S19. Neuron imaging data **S36**
Figure S20. Recombinant ^{15}N- αS-$^{Ac}K_{12}$ **S37**
Figure S21. Recombinant ^{15}N- αS-$^{Ac}K_{43}$ **S37**
Figure S22. Recombinant ^{15}N- αS-$^{Ac}K_{80}$ **S37**
Figure S23. HSQC spectra acquired for αS-$^{Ac}K_{12}$, $^{Ac}K_{43}$ or $^{Ac}K_{80}$ in buffers **S38**
Figure S24. Chemical shift perturbation from WT calculated at each residue of αS-WT, $^{Ac}K_{43}$ or $^{Ac}K_{80}$. **S41**
Figure S25. Chemical shift perturbation calculated at each residue of vesicle-bound αS-WT, $^{Ac}K_{12}$,$^{Ac}K_{43}$ or $^{Ac}K_{80}$ **S41**
Figure S26. HSQC spectra acquired for vesicle-bound αS-WT, $^{Ac}K_{12}$,$^{Ac}K_{43}$ or $^{Ac}K_{80}$ **S42**
Figure S27. Recombinant αS-C_{114} and fluorescent labeling **S46**
Figure S28. Recombinant αS- $^{Ac}K_{12}C_{114}$ and fluorescent labeling **S46**
Figure S29. Recombinant αS- $^{Ac}K_{43}C_{114}$ and fluorescent labeling **S47**
Figure S30. Recombinant αS- $^{Ac}K_{80}C_{114}$ and fluorescent labeling **S47**
Figure S31. Vesicle FCS data **S48**
Figure S32. Lipid binding affinity of acetylated αS determined by FCS **S49**
Figure S33. TEM images of ^{Ac}K fibrils **S50**
Figure S34. Cryo-EM structure from 25% $^{Ac}K_{80}$ fibril preparation **S52**

Figure S35. Map and model validation **S54**
Figure S36. Local resolution cryo-EM maps for WT fibrils **S55**
Figure S37. Local resolution cryo-EM maps for $^{Ac}K_{80}$ fibrils **S56**
Figure S38. Cryo-EM structures of WT and $^{Ac}K_{80}$ fibrils **S57**
Figure S39. Comparison of cryo-EM density for $^{Ac}K_{80}$ fibrils formed in TBS and PBS **S58**
Figure S40. Comparison of $^{Ac}K_{80}$ fibril structure to other cryo-EM structures **S59**
Figure S41. MS spectra of $^{Ac}K_{12}$ peptide from αS $^{Ac}K_{12}$ standard **S60**
Figure S42. MS spectra of $^{Ac}K_{43}$ peptide from αS $^{Ac}K_{43}$ standard **S60**
Figure S43. MS spectra of $^{Ac}K_{80}$ peptide from αS $^{Ac}K_{80}$ standard **S61**
Figure S44. MS spectra of unmodified K_{12} peptide from αS WT standard **S61**
Figure S45. MS spectra of unmodified K_{43} peptide from αS WT standard **S62**
Figure S46. MS spectra of unmodified K_{80} peptide from αS WT standard **S62**
Figure S47. Representative MS spectra of $^{Ac}K_{12}$ peptide from αS in patient sample 2 ....**S63**
Figure S48. Representative MS spectra of $^{Ac}K_{43}$ peptide from αS in patient sample 15 ....**S63**
Figure S49. Representative MS spectra of $^{Ac}K_{80}$ peptide from αS in patient sample MSA5-3675 ....**S64**

Schemes:

Scheme S1. Semi-synthesis scheme for creating αS-$^{Ac}K_{80}$ **S19**

Tables:

Table S1. Cryo-EM particle numbers for fibril samples **S15**
Table S2. Statistics of cryo-EM data collection and refinement for 25% $^{Ac}K_{80}$ fibrils **S51**
Table S3. Statistics of cryo-EM data collection and refinement for WT and 100% $^{Ac}K_{80}$ fibrils **S53**
Table S4. Quantification of acetylation %ratio by LC-MS/MS data analysis **S65**
Table S5. Ionization Efficiency of Acetylated Tryptic Peptides **S66**

General Information

Reagents for peptide synthesis, including 2-(*1H*-benzotriazol-1-yl)-1,1,3,3-tetramethyluronium hexafluorophosphate (HBTU), *N*,*N*-diisopropylethylamine (DIPEA), and Fmoc-amino acids, were purchased from EMD Millipore (Burlington, MA, USA) or ChemImpex International (Wood Dale, IL, USA). Reagents for native chemical ligation (NCL): $NaNO_2$, *tris*(2-carboxyethyl)phosphine (TCEP), and mercaptophenyl acetic acid (MPAA) were purchased from Sigma-Aldrich (St. Louis, MO, USA). *E. coli* BL21(DE3) cells and *E. coli* Dh5α cells were purchased from New England Biotechnologies (Ipswich, MA, USA). DNA oligomers were purchased from Integrated DNA Technologies, Inc (Coralville, IA, USA). DNA extraction and Miniprep kits were purchased from Qiagen (Hilden, Germany). Buffers were made with MilliQ filtered (18 MΩ) water (Millipore; Billerica, MA, USA). Preparation of the pTXB1-αS-intein-H_6 plasmid containing α-synuclein (αS) with a C-terminal fusion to the *Mycobacterium xenopi* GyrA intein and C-terminal His_6 tag was described previously.(Batjargal et al., 2015) This plasmid was used as a starting point for the preparation of αS (mutants) -intein constructs. For overexpression of αS in HEK cells, the expression vector pcDNA5/TO was purchased from Thermo Fisher Scientific (Waltham, MA). pTECH-chAcK3RS (IPYE) was a gift from David Liu via Addgene (plasmid # 104069 ; http://n2t.net/addgene:104069 ; RRID:Addgene_104069; Watertown, MA, USA). Acetyllysine was purchased from ChemImpex. Nicotinamide was purchased from Alfa Aeser (Tewksbury, MA, USA). Atto 488 maleimide was purchased from Sigma-Aldrich. Matrix-assisted laser desorption/ionization mass spectrometer (MALDI-MS) data were collected with a Bruker Ultraflex III MALDI-MS instrument or a Bruker Microflex MALDI-MS (Billerica, MA, USA). UV/Vis absorbance spectra were obtained with a Hewlett-Packard 8452A diode array spectrophotometer (currently Agilent Technologies; Santa Clara, CA). Gel images were obtained with a Typhoon FLA 7000 (GE Lifesciences; Princeton, NJ, USA). Thioflavin T (ThT) absorbance spectra were collected on a Tecan SPARK plate reader (Mannedorf, Switzerland). Proteins were purified on a 1260 Infinity II preparative high-performance liquid chromatography (HPLC) system (Agilent Technologies). NCL reactions were monitored on a 1260 Infinity II Analytical HPLC system (Agilent Technologies) using a Jupiter C4 column (Phenomenex; Torrance, CA, USA). Water + 0.1% trifluoroacetic acid (TFA) (solvent A) and acetonitrile + 0.1% TFA (solvent B) were used as the mobile phase in HPLC.

Protein semi-synthesis for generation of αS -$^{Ac}K_{80}$.

To synthesize αS acetylated at K_{80}, an N-to-C three-part native chemical ligation (NCL) was performed between the fragments $αS_{1-76}$, $αS_{77-84}$-$^{Ac}K_{80}$, and $αS_{85-140}$. All fragments and intermediate products were purified by reverse-phase high-performance liquid chromatography (RP-HPLC) over a C4 column.

N-terminal thioester fragment $αS_{1-76}$-MES (**1a**) and C-terminal fragment $αS_{85-140}$-C_{85} (**4**) were constructed through deletion polymerase chain reaction (PCR) of previously published αS fragment constructs. They were each recombinantly expressed as a fusion with a polyhistidine-tagged GyrA intein from *Mycobacterium xenopi*. The N-terminal thioester was generated by adding excess sodium mercaptoethane sulfonate (MESNa) to cleave the intein by N,S-acyl shift.(Muir et al., 1998) (reported yield 24.1 mg/L.(Pan et al., 2020)) Endogenous methionyl aminopeptidase processes the N-terminus of the 85-140 peptide to expose the N-terminal cysteine,(Xiao et al., 2010) which further reacts with aldehydes or ketones *in vivo* to form thiazolidine derivatives.(Liu et al., 2016) The thiazolidine derivatives were deprotected with methoxyamine to give a free N-terminal cysteine (4.40 mg/L, Figure S1b).

The middle peptide $αS_{77-84}$-$Pen_{77}$$^{Ac}K_{80}$-$NHNH_2$ (**2**, Pen: penicillamine(Haase et al., 2008)) was synthesized as a C-terminal acyl hydrazide through Fmoc-based, solid-phase peptide synthesis (Yield: 12.4 mg, 12 μmol, 48%, Figure S1a).

$αS_{1-76}$-MES (**1a**) and $αS_{77-84}$-$Pen_{77}$$^{Ac}K_{80}$-$NHNH_2$ **(2)** were ligated overnight under routine NCL conditions in the presence of MPAA. (Yield: 1.46 mg, 172 nmol, 57%, Figure S1d). The purified product (**3a**) was activated to MES thioester (**3b**) (Yield: 1.29 mg, 126 nmol, 73%, Figure S1c). The second ligation between $αS_{1-84}$-$Pen_{77}$$^{Ac}K_{80}$-MES (**3b**) and $αS_{85-140}$-C_{85} (**4**) was performed in the presence of methyl thioglycolate(Huang et al., 2016) to allow for desulfurization without intermediate purification (Figure S1e). The product αS-$^{Ac}K_{80}$ (**5b**) was obtained in 43% yield (0.90 mg, 62 nmol, Figure S1f).

Construction of expression plasmids

The following primers were designed for site-directed mutagenesis to introduce TAG (= Z) codons at each lysine acetylation site. Site-directed mutagenesis for TAG mutations were performed on the plasmid encoding αS-MxeGyrA-His_6.

Primer sequences:

αS-Z_{12}	Forward	5’-CAAAGGCCTAGGAGGGAGTT-3’
	Reverse	5’-AAAGTCCTTTCATGAATACATCCATATGTATA-3’
αS-Z_{21}	Forward	5’-GTGGCTGCTGCTGAGTAGACCAAACAGGGT-3’
	Reverse	5’-AACTCCCTCCTTGGCCTTTGAAAGTCCTTT-3’
αS-Z_{23}	Forward	5’-GCTGCTGAGAAAACCTAGCAGGGTGTGGCA
	Reverse	5’-AGCCACAACTCCCTCCTTGGCCTTTGAAAG
αS-Z_{32}	Forward	5’-GCAGAAGCAGCAGGATAGACAAAAGAGGGT
	Reverse	5’-CACACCCTGTTTGGTTTTCTCAGCAGCAGC
αS-Z_{34}	Forward	5’-GCAGCAGGAAAGACATAGGAGGGTGTTCTC
	Reverse	5’-TTCTGCCACACCCTGTTTGGTTTTCTC
αS-Z_{43}	Forward	5’-GTAGGCTCCTAGACCAAGG
	Reverse	5’-ATAGAGAACACCCTCTTTTGTCTTTC
αS-Z_{45}	Forward	5’-GGCTCCAAAACCTAGGAGGGAGTGGTG
	Reverse	5’-TACATAGAGAACACCCTCTTTTGTCTTTCCTGC
αS-Z_{58}	Forward	5’-GCAACAGTGGCTGAGTAGACCAAAGAGCAA
	Reverse	5’-CACACCATGCACCACTCCCTCCTTGGT
αS-Z_{60}	Forward	5’-GTGGCTGAGAAGACCTAGGAGCAAGTGACA
	Reverse	5’-TGTTGCCACACCATGCACCACTCC
αS-Z_{80}	Forward	5’-GCAGTAGCCCAGTAGACAGTGGAGGGA
	Reverse	5’-TGTCACACCCGTCACCACTGC
αS-Z_{96}	Forward	5’-GCCACTGGCTTTGTCTAGAAGGACCAGTTG
	Reverse	5’-TGCTGCAATGCTCCCTGCTCCCTC
αS-Z_{102}	Forward	5’-CAGTTGGGCTAGAATGAAGAAGG
	Reverse	5’-GTCCTTTTTGACAAAGCCAGTG

Production of recombinant αS constructs

To generate acetylated αS, each plasmid encoding αS with a TAG mutation at the site of interest and a machinery plasmid for acetyllysine incorporation, pTECH-chAcK3RS (IPYE), were co-transformed by heat shock at 42 °C into BL21 (DE3) competent cells. Cells were plated and incubated on ampicillin/chloramphenicol (Amp/Chlor) plates. Single colonies were picked to inoculate primary cultures in LB media supplemented with 0.1 mg/mL Amp and 0.025 mg/mL Chlor. Primary cultures were incubated overnight or until they were cloudy at 37 °C. Secondary cultures in LB media were inoculated and grown at 37 °C with shaking at 250 rpm until the optical density reached ~ 0.6. The culture was subsequently cooled to 18 °C. 50 mM nicotinamide and 10 mM ε-acetyl lysine were added to the culture and incubated for ~ 5 min before inducing the expression of the gene of interest with 1 mM isopropyl ß-D-1-thiogalactopyranoside (IPTG). To generate isotopically labeled, acetylated αS, protein expression was performed as above, except that cells were grown in M9 minimal media(Anderson, 1946) that contains ^{15}N-ammonium chloride. Induced cells were then grown in the shaker-incubator at 18 °C overnight. After centrifugation (4000 rpm, 20 min, 4 °C), cell pellets were re-suspended in buffer (40 mM Tris pH 8.3, with one Roche protease inhibitor tablet) and sonicated in a cup in an ice bath (5 min, 1 s ON, 2 s OFF). The

resulting lysate was centrifuged (14,000 rpm, 30 min, 4 °C), and supernatant containing the αS variant was purified over a Ni-NTA affinity column. Intein cleavage was carried out by incubation with 200 mM β-mercaptoethanol (βME) on a rotisserie overnight at room temperature. Cleaved αS variant was dialyzed into 20 mM Tris, pH 8 buffer before purification over a second Ni-NTA column to remove the free intein from the sample. The αS proteins were purified by RP-HPLC over a C4 column (acetylated proteins) or by fast-protein liquid chromatography (FPLC) using a Hi-Trap Q 5 mL column (glutamine mutants), dialyzed into 1x phosphate buffered saline (PBS) and spin-concentrated. For purification of acetylated Cys mutants for fluorescent labeling, TCEP was added to a final concentration of 1 mM prior to HPLC injection and samples were dialyzed into 20 mM Tris 50 mM NaCl pH 7.4 after purification. Upon flash-freezing, protein stocks were kept at -80 °C until further use. Isotopically labeled αS samples were lyophilized after HPLC purification.

Fluorescent labeling

To label αS-$^{Ac}K_XC_{114}$, the protein stocks in 20 mM Tris, 50 mM NaCl, pH 7.4 were incubated with 2-10 eq. TCEP, then 10 eq. Atto 488-maleimide dye was added and incubated at room temperature for 2-4 hours or at 4 °C for overnight until product formation was observed by MALDI-MS. The product was purified by HPLC over a C4 column and dialyzed into 20 mM Tris, 50 mM NaCl, pH 8.

Circular Dichroism (CD)

αS samples were filtered through 100 kDa-cutoff spin concentrators and the concentrations were determined by UV-Vis absorbance. Based on the quantification, all the αS samples were first diluted to 15 μM with 1x PBS. Samples for CD acquisition were then prepared in triplicate by dilution of the protein stock solutions using 4.3 mM Na_2HPO_4, 1.47 mM KH_2PO_4, pH 7.4, and mixing with 100 mM sodium dodecyl sulfate (SDS) in 4.3 mM Na_2HPO_4, 1.47 mM KH_2PO_4, pH 7.4 to yield samples composed of 5 μM αS with 10 mM SDS in 45.7 mM NaCl, 0.9 mM KCl, 4.3 mM Na_2HPO_4, 1.47 mM KH_2PO_4, pH 7.4. CD spectra were acquired on a JASCO J-1500 spectrometer in quartz cuvettes with a path length of 1 mm at 25 °C. Spectra were collected over the range of 190-260 nm using a 1 nm data pitch, 2 nm bandwidth, 8 s data integration time and 50 nm/min scanning speed. At each wavelength, spectra were background subtracted for buffer blank and corrected for concentration, path length, and number of residues as described by the following equation, where θ_{sample} and θ_{blank} refer to raw ellipticity values for the sample and buffer blank, respectively, ε is the path length of the cuvette, *c* is the protein concentration, and *n* is the number of residues.

$$[\theta] = \frac{((\theta_{sample} - \theta_{blank}) - (\theta_{sample,260nm} - \theta_{blank,260nm}))}{10\ \times \varepsilon \times c \times n} \times 10^{-3}$$

To determine the effects on helicity, the $[\theta_{222}]$ value acquired for each acetylated αS was normalized by the $[\theta_{222}]$ value of αS WT.

Protein aggregation kinetics and percentage incorporation into fibrils

Aggregation of αS monomer seeded by preformed fibrils (PFFs) of wild type (WT) αS was performed with 100% WT αS or with mixtures of 10% or 25% acetylated αS or αS Gln mutants. The seeding experiments were carried out by agitation at 1400 rpm at 37 °C and monitored by ThT. αS WT monomer in buffer (1x phosphate-buffered saline (PBS), pH 7.4) was prepared as a 40 μM stock solution and a 50 μL aliquot was added to each well of a 96-well half area clear bottom plate (6 replicates per one construct). The plate was sealed with a plastic film and incubated at 37 °C for 30 min before aggregation. PFF seeds were prepared by resuspending fibrils in PBS to make a 4 μM stock solution and freshly sonicating in an Eppendorf tube in an ice bath (2 min, 1 s ON, 1 s OFF). A 50 μL aliquot of sonicated seeds was added to monomers in each well (10% seeds). Samples were shaken on an IKA MS3 orbital shaker set to 1400 rpm at 37 °C. At each time point, ThT fluorescence was measured on a Tecan SPARK plate reader (excitation: 450 nm, emission: 485 nm, emission bandwidth: 5 nm, integration time: 40 μs). The extent of aggregation was determined based on normalized fluorescence intensity at 485 nm calculated from the minimum intensity and maximum intensity of each replicate. The data points were fit using GraphPad Prism software with the nonlinear regression model using the following equation:

$$y = Mi + \frac{Ma - Mi}{1 + \left(\frac{T_{1/2}}{x}\right)^{z}}$$

where Mi and Ma are the minimal and maximum y values, respectively, $T_{1/2}$ is the time at the mid-point of aggregation, and z is a parameter that determines the steepness of the curve. For the plots in Figure 4, Aggregation time was normalized to $T_{1/2}$ of the WT control, performed in parallel, and minimum and maximum fluorescence values were also normalized.

After the final time point, samples were pelleted at maximum speed on a tabletop centrifuge for 90 min. The supernatant was removed, and pellet was resuspended in the original volume of buffer. Samples were supplemented with SDS to a 25 mM final concentration, boiled for 20 min, and chilled on ice. Monomeric samples for calibration were prepared by 2-fold serial dilutions in water. All samples were analyzed by SDS-PAGE (4-15 or 4-20% acrylamide). Gels were stained with Coomassie Brilliant Blue dye.

Quantification of the intensity of bands was done using the ImageJ software (National Institutes of Health; Bethesda, MD, USA. Values reported for aggregation kinetics and monomer incorporation are the average and standard error of mean taken from independent replicates.

In situ aggregation in cultured primary hippocampal neurons

Primary hippocampal neurons were isolated from mouse neonate brain and plated on a Thermo Fisher Scientific 96-well plate at a density of 350 cells/µL in 100µL of neurobasal (NB) media with 5% Fetal Bovine Serum. After 24 hours of incubation, the media was replaced by aspiration with fresh NB media. PFFs were formed by agitation for 3 days at 3500 rpm at 37 °C for both WT αS and post-translationally modified variants for the following conditions: 25% $^{Ac}K_{12}$, 25% $^{Ac}K_{43}$, 25% $^{Ac}K_{80}$. At 8 days in vitro (DIV), PFF constructs were sonicated in (QSonica Microson XL-2000) for 20 cycles (1s ON, 1s OFF) and used to treat the neurons for a final concentration of 50 ng/µL. The neurons were cultured for an additional 14 days following treatment of the fibril seeds, during which the medium was exchanged every 7 days. At 21 DIV, the cells were fixed with a final concentration of 4% paraformaldehyde (Electron Microscopy Sciences) + 4% sucrose (Sigma-Aldrich) in PBS, washed 3x with PBS (Sigma-Aldrich), and permeabilized with 0.1% Triton X-100 (Thermo Fisher). After 3x washes with PBS, the plate was then incubated overnight in fluorescent western blot blocking buffer (Rockland Immunochemical) and probed with 1:3000 phosphoserine 129 antibody (m81A(Waxman and Giasson, 2008)) for detection of intracellular αS aggregates and neuronal cytoskeleton antibody (MAP2(Volpicelli-Daley et al., 2011)) for detection of neuronal processes in blocking buffer at room temperature for 4 hours. Samples were washed 3x with PBS and then incubated with 1:1000 fluorophore-conjugated secondary antibodies in blocking buffer at room temperature for 1 hour. The plate was then immediately washed 3x with PBS and imaged in 75 µL of PBS (IN Cell Analyzer 2200/2000). Quantification of 81A signal was done using ImageJ. All ^{Ac}K variants were found to have significantly less *in situ* recruitment compared to WT by One-Way ANOVA and Tukey post-hoc (GraphPad Prism 9.3.1).

Preparation of synthetic vesicles

For NMR experiments, 60:25:15 1,2-dioleoyl-sn-glycero-3-phosphocholine:1,2-dioleoyl-sn-glycero-3-phosphatidylethanolamine:1,2-dioleoyl-sn-glycero-3-phospho-L-serine (DOPC:DOPE:DOPS) small unilamellar vesicles (SUVs) were generated as described previously(Dikiy and Eliezer, 2014) to mimic the size and lipid composition of native synaptic vesicles. In brief, mixtures of DOPC, DOPE, and DOPS dissolved in chloroform (Avanti Polar Lipids, Alabaster, AL) were dried under nitrogen the lipid film was resuspended in NMR buffer (10 mM Na2HPO4, 100 mM NaCl, 10% v/v D2O, pH 6.8), immersed in a bath

sonicator for 15 min at a time until clear, and further clarified by ultracentrifugation at 130,000 × g for 2 h. The supernatant was stored at 4 °C as SUV stock solution and was used for NMR experiments within 1 day.

For fluorescence correlation spectroscopy, lipid vesicles were prepared by extrusion through porous membranes. A mixture in 50:50 molar ratio of 1-palmitoyl-2-oleoyl-sn-glycero-3-phosphoserine (POPS) and 1-palmitoyl-2-oleoyl-sn-glycero-3-phosphocholine (POPC) were drawn from chloroform stock and dried under nitrogen gas to form a film inside a glass vial. Films were desiccated under vacuum and re-hydrated in 20 mM 3-(*N*-morpholino)propanesulfonic acid (MOPS), 147 mM NaCl, 2.7 mM KCl, pH 7.4. Ten freeze-thaw cycles consisting of cooling in liquid nitrogen for 40 s and warming in a 60 °C water bath for 2 min were performed to aid the formation of uniformly sized vesicles. With syringes, vesicles were then extruded 31 times through stacked 50 nm pore membranes held in place inside an extruder. Vesicles were determined by dynamic light scattering (DLS) to be monodisperse and distributed uniformly around 80 nm in diameter, consistent across different concentrations of all samples. All lipid vesicles were prepared fresh and used within 48 h of extrusion.

Heteronuclear single quantum coherence spectroscopy (HSQC)

Lyophilized WT or lysine-acetylated αS mutants were dissolved in NMR buffer (10 mM Na_2HPO_4, 100 mM NaCl, 10% v/v D_2O, pH 6.8) and mixed in a 1:1 volume ratio with SUV stock solution or NMR buffer. Final protein concentrations were ca. 100 µM and final SUV lipid concentrations were ca. 3 mM, assuming complete conversion of lipids to SUVs. NMR ^{1}H-^{15}N heteronuclear single quantum coherence (HSQC) experiments were acquired at 10 °C using a 500 MHz Bruker Avance spectrometer equipped with a cryogenic probe using TopSpin 3.2 software. Data processing and analysis were performed using NMRbox(Maciejewski et al., 2017), NMRPipe(Delaglio et al., 1995), and NMRFAM-SPARKY(Lee et al., 2015) software. Amide resonance peaks were assigned based on previously published chemical shift assignments for WT αS monomer(Eliezer et al., 2001). NMR peak intensity ratios were calculated as the ratio of peak intensity in the presence of SUVs to that in the absence of SUVs for matched samples. To correct for protein concentration variations in the samples, the intensity ratios were normalized by the average ratio for the C-terminal 40 residues, which do not feature appreciable membrane interactions at these lipid concentrations.

Fluorescence correlation spectroscopy (FCS)

Fluorescence correlation spectroscopy (FCS) experiments to study the binding of αS-$^{Ac}K_{43}C^{488}_{114}$ and αS-$^{Ac}K_{80}C^{488}_{114}$, including preparation of synthetic lipid vesicles, collection of FCS data, and data analysis,

were carried out as described previously for arginylated αS.(Pan *et al.*, 2020) Eight-well chambered coverglasses (Nunc, Rochester, NY, USA) were prepared by plasma cleaning followed by incubation overnight with polylysine-conjugated polyethylene glycol (PEG-PLL), prepared using a modified Pierce PEGylation protocol (Pierce, Rockford, IL, USA). PEG-PLL coated chambers were rinsed with and stored in Milli-Q water until use. FCS measurements were performed on a lab-built instrument based on an Olympus IX71 microscope with a continuous emission 488 nm DPSS 50 mW laser (Spectra-Physics; Santa Clara, CA, USA). All measurements were made at 20 °C. The laser power entering the microscope was adjusted to 4.5 µW. Fluorescence emission collected through the objective was separated from the excitation signal through a Z488rdc long pass dichroic filter and an HQ600/200m bandpass filter (Chroma; Bellows Falls, VT, USA). Emission signal was focused onto the aperture of a 50 µm optical fiber. Signal was amplified by an avalanche photodiode (Perkin Elmer; Waltham, MA, USA) coupled to the fiber. A digital autocorrelator (Flex03Q-12, correlator.com; Bridgewater, NJ, USA) was used to collect 10 autocorrelation curves of 10 seconds for each measurement of free protein in buffer without lipids or 30 autocorrelation curves of 30 seconds for each measurement in the presence of lipid vesicles. Fitting was done using lab-written code in MATLAB (The MathWorks; Natick, MA, USA).

To determine the diffusion time of each protein construct, each αS variant labeled with Atto 488 was measured in buffer without lipid. The average of 10 autocorrelation curves was fit to a 1-component autocorrelation function:

$$G(\tau) = \frac{1}{N}\left(\frac{1}{1+\frac{\tau}{\tau_1}} * \left(\frac{1}{1+\frac{s^2\tau}{\tau_1}}\right)^{1/2}\right)$$

where $G(\tau)$ is the autocorrelation function, N is the number of molecules in the focal volume, τ_1 is the diffusion time of αS, and s is the radial-to-axial ratio of the excitation volume. The counts per molecule (CPM) for each sample was calculated by dividing the average intensity (Hz) of the measured signal by the number of molecules N. The normalized CPM of each αS was calculated by dividing by the CPM of freely diffusing fluorescent standard Alexa Fluor 488.

Vesicle binding affinity

αS constructs labeled with Atto488 were examined in the presence of varying concentrations (0.001 mM to 0.5 mM lipid) of lipid vesicles consisting of 50:50 POPS/POPC. The average of 30 autocorrelation curves was fit to a 2-component equation:

$$G(\tau) = \frac{1}{N}\left(A * \frac{1}{1+\frac{\tau}{\tau_1}} * \left(\frac{1}{1+\frac{s^2\tau}{\tau_1}}\right)^{1/2} + Q * (1-A) * \frac{1}{1+\frac{\tau}{\tau_2}} * \left(\frac{1}{1+\frac{s^2\tau}{\tau_2}}\right)^{1/2}\right)$$

where $G(\tau)$ is the autocorrelation function, N is the number of molecules in the focal volume, τ_1 is the characteristic diffusion time of αS, τ_2 is the characteristic diffusion time of the vesicles, s is the radial-to-axial ratio of the excitation volume, Q is the ratio of the brightness of vesicle-bound αS relative to αS, and A is the fraction of free αS. When fitting the autocorrelation curves for αS in the presence of lipid vesicles, the diffusion time of bound and unbound αS were respectively fixed to experimentally determined values. The diffusion time of unbound protein, τ_1, was determined by measurements of the protein in buffer without lipids. Since bound protein diffuses with the vesicles to which they are bound, the diffusion time of the vesicles, τ_2, was determined by measurements of the protein in the presence of a concentration of vesicles that gave the maximum diffusion time (0.1 mM lipid). In the binding assay, the fraction of αS bound at each lipid concentration was obtained from the fit to each autocorrelation curve. Averages and standard deviations were calculated from at least 3 independent measurements performed on separate days at each lipid concentration. The resulting binding curve was fit to the following equation, from which the $K_{d,app}$ was determined.

$$A = \frac{B_{max}x}{K_{d,app} + x}$$

where A is the fraction of αS bound, x is the accessible lipid concentration, B_{max} is the maximum fraction of αS bound, and $K_{d,app}$ is the apparent dissociation constant.

Transmission electron microscopy (TEM)

TEM imaging was carried out on an FEI Tecnai T12 instrument (Hillborough, OR, USA) with an accelerating voltage of 100 kV. Fibril samples were prepared by shaking 100 µM αS (WT with 25% ^{Ac}K variants) at 1500 rpm for 72 hours, then diluted into water at a final concentration of 0.1 mg/mL. A 5 µL drop of sample was deposited on glow discharged carbon Formvar coated 300-mesh Cu grids and allowed to rest for 1 min at room temperature. 5 µL of stain (2% w/v uranyl acetate in water) was then applied to the grid. The liquid was wicked off with grid paper, and another 5 µL of stain was applied and wicked off. Images were collected at magnifications ranging from 11000x to 42000x.

Cryo-electron microscopy (Cryo-EM) data acquisition

PFFs for cryo-EM studies were prepared as following: αS monomer in buffer (PBS: 1x PBS, pH 7.4; TBS: 20 mM Tris 140 mM sodium chloride pH 7.0 at 37 ºC) was prepared as 100 µM stock solutions. The monomers were incubated at 37 ºC for 30 minutes prior to shaking to ensure that the same temperature is adjusted. Samples were then shaken at 1500 rpm at 37 ºC in Eppendorf tubes for overnight.

Prior to vitrification, fibril samples were concentrated 10-fold by centrifugation at 14,000 rpm for 10 minutes using a Beckman Microfuge 18 centrifuge (Beckman Coulter; Indianapolis, Indiana) to a final concentration of approximately 1 mM. A 3.5 µl aliquot was applied to a glow-discharged holey copper grid (Quantifoil R2/1, 300 mesh) equilibrated at 4 °C and 100% humidity. After a 1-second blot, the grids were plunge-frozen into liquid ethane using a Vitrobot Mark IV (Thermo Fisher). Cryo-EM images were acquired on a 200 kV Talos Glacios microscope equipped with a Falcon 4i camera and Selectris energy filter. The magnification was set to 100,000X, resulting in a calibrated pixel size of 1.16 Å/pix. To minimize radiation damage, the total dose applied to the samples was limited to 40 e-/Å^2 delivered over a 5.01-second exposure time using 1539 movie frames with electron event representation (EER) fractions of 27. A defocus range of -2.5 to -0.8 µm was employed along with a spherical aberration of 2.7 mm. The microscope was operated at 200 kV with a 50 µm objective aperture and a 20 eV energy filter. For further analysis, a total of 5387, 4038, and 5001 images were collected for 25% $^{Ac}K_{80}$, 100% $^{Ac}K_{80}$, and WT αS fibril preparations, respectively.

Cryo-EM data processing

Cryo-EM data processing began with CryoSPARC v4.1.1 (Structura Biotechnology Inc.; Toronto, Canada).(Punjani et al., 2017) EER data files were imported with 27 fractions and dose rate of 1 e$^-$/Å^2/fraction. Patch Motion correction and Patch CTF estimation were applied to the imported data. For initial filament picking, template-free filament tracing was used on all images, targeting filaments with diameters between 50 and 250 nm. Particles of size 768x768 pixels with 12.5 or 50 Å inter-box distance were extracted and subjected to 2D classification (see Table S1 for numbers of template-free filament tracing particles for each fibril sample). The best 3 classes were then selected and used to create a template for a second round of filament tracing on the entire dataset. Following filament tracing, Particles were again extracted from the micrographs with a box size of 512 pixels and underwent 3 rounds of 2D classification with only a subset of particles retained (see Table S1 for numbers of extracted particles and retained for each fibril sample). CryoSPARC (.cs) files were then converted to Star files using the PyEM csparc2star.py script. A custom Python script, generated using ChatGPT, was used to extract the start-to-end coordinates

of the filaments. The createAutopick function from cryolo_boxmanager_tools.py in crYOLO(Wagner et al., 2019) was used to generate a STAR file that links particle coordinates to movie files, which was then used for particle extraction.

For 25% αS-$^{Ac}K_{80}$ preparations, particles were re-extracted in Relion 4.0.0(Kimanius et al., 2021) using 3 unique asymmetric units and box size of 256 pixels (see Table S1 for numbers of re-extracted particles). 2D classification was then conducted using Relion with Regularization parameter (T) of 2, in-plane angular sampling of 2 degrees and 20 iterations using the EM algorithm. An initial model was generated from the good 2D classes using the relion_helix_inimodel2d function in Relion (see Table S1 for numbers of initial model particles). 3D auto-refinement was employed to obtain the cryo-EM map with soft-edge mask (see Table S1 for numbers of selected 3D classification particles). A final map with resolution of 2.88 Å was obtained after particle Bayesian polishing and CTF refinement.

For WT and 100% αS-$^{Ac}K_{80}$ fibril preparations (from both TBS and PBS), particles were re-extracted in Relion 5.0-beta-1(Burt et al., 2024) using 3 unique asymmetric units and a box size of 256 pixels (see Table S1 for numbers of re-extracted particles). Initial maps were generated using the helical refinement function in CryoSPARC (see Table S1 for numbers of initial model particles for each fibril sample). 3D auto-refinement in Relion was employed to obtain the cryo-EM map with soft-edge masks. However, blurry maps were observed for both WT αS and $^{Ac}K_{80}$ αS in one of two chains, indicating the presence of multiple structures.(Lövestam and Scheres, 2022) Two runs of 3D classification were then performed with 50 iterations, with alignment and optimization of twist and rise parameters to separate the two structures (see Table S1 for numbers of selected 3D classification particles for each fibril sample). Two maps with similar backbone traces but different alignments were obtained (A and B polymorphs for WT and αS-$^{Ac}K_{80}$ for both PBS and TBS preparations, see Figure 8 in main text). Final maps were obtained after particle Bayesian polishing and CTF refinement.

Model building and refinement

For 25% αS-$^{Ac}K_{80}$ preparation, the cryo-EM map was first fitted with the PDB ID 6a6b structure in Chimera.(Li et al., 2018) The resulting atomic model was then adjusted in Coot according to the map. Next, the model was refined in real space using PHENIX 1.21.1.(Liebschner et al., 2019) For cross validation, all atoms in the refine model were randomly displaced 0.3 Å and refined against the first half map from Relion 3D auto-refinement. Final statistics for the 25% αS-$^{Ac}K_{80}$ preparation are given in Table S2.

For WT (TBS) and 100% αS-$^{Ac}K_{80}$ (PBS) fibril preparations, cryo-EM maps were first fitted with a single chain of the PDB ID 6rt0 structure(Guerrero-Ferreira et al., 2019) for WT αS and a single chain of the PDB

ID 8pix structure(Frey et al., 2024) for αS-$^{Ac}K_{80}$ in Chimera. The resulting atomic models were then adjusted, and layers were added in Coot according to the map. Next, models were refined in real space using PHENIX 1.21.1. For cross validation, all atoms in the refined model were randomly replaced by 0.3 Å and refined against the first half map from Relion 3D auto-refinement. Final statistics for the WT and αS-$^{Ac}K_{80}$ fibril polymorphs are given in Table S3.

Table S1. Cryo-EM particle numbers for fibril samples

	**25% $^{Ac}K_{80}$**	**WT TBS**	**WT PBS**	**$^{Ac}K_{80}$ TBS**	**$^{Ac}K_{80}$ PBS**
Template-Free Filament Tracing Particles	4,942,459	650,880	2,031,379	871,724	6,247,663
Extracted Particles	3,364,884	1,163,693	269,189	332,878	4,158,103
Retained Particles	228,881	764,292		199,843	705,709
Re-extracted Particles	338,172	2,341,469		834,159	893,660
Initial Model Particles	338,081	2,341,469		834,159	893,660
3D Classification Particles	296,473				
3D Classification Particles A		696,830		48,610	102,778
3D Classification Particles B		1,317,407		312,253	200,042

Preparation of human HDAC8

Recombinant human HDAC8 was expressed and purified as previously described with minor modifications(Decroos et al., 2014; Dowling et al., 2008; Osko et al., 2021). Briefly, the HDAC8-6His-ET20b expression plasmid was grown in BL21-DE3 cells, and a 250 mL culture was incubated overnight in Lysogeny Broth (LB) media supplemented with 100 µg/mL of ampicillin at 37 ºC. Aliquots of this culture were used to inoculate 6 x 1 L cultures of 2XYT media supplemented with 100 µg/mL of ampicillin. Cultures were grown at 37 ºC until reaching OD = 0.6–0.8; after cooling to 18 °C, cultures were induced by the addition of 0.4 mM isopropyl-β-D-thiogalactopyranoside (IPTG) and 0.5 mM $ZnSO_4$ and grown overnight. Cells were pelleted by centrifugation (5244g, 15 min). Purification was achieved exactly as recently summarized(Osko *et al.*, 2021) with slight modification of the lysis buffer: 50 mM Tris (pH 8.0), 500 mM KCl, 5% w/v glycerol, and the reducing agent 1 mM TCEP in place of 3 mM β-mercaptoethanol (BME).

HDAC8 deacetylation assay

Acetylated αS substrates (αS $^{Ac}K_X$) and ^{15}N-labeled αS WT were buffer-exchanged into HDAC8 reaction buffer (50 mM Tris, 137 mM NaCl, 2.7 mM KCl, 1 mM $MgCl_2$). Concentrations of each was quantified and deacetylation reaction was setup in triplicate, with final concentrations of HDAC8 8µM, 1.25 µM αS $^{Ac}K_X$, 1.25 µM ^{15}N-αS WT. The reaction was performed at room temperature for 24 h. MALDI-TOF-MS spectra were collected for each reaction, at 0h and 24h timepoints. Peak picking and peak area quantification, via fitting to Gaussian curves, for deacetylated product (^{14}N-αS WT) and the standard (^{15}N-αS WT) were performed on the Bruker flexAnalysis software.

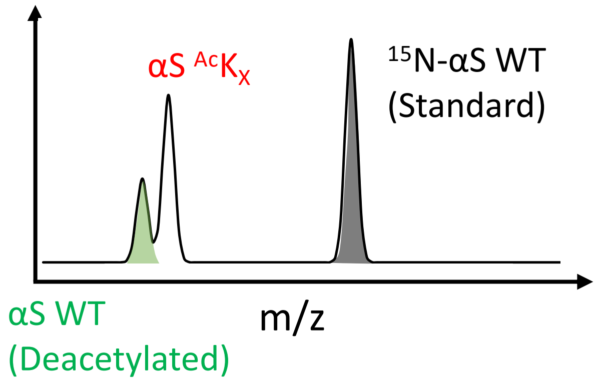

The formula below was used to calculate the increase in %Deacetylated.

$$\Delta\%_{Deacetylated} = \frac{\Delta Peak\ Area\ (^{14}N\ \alpha S_{WT})}{\Delta Peak\ Area\ (^{15}N\ \alpha S_{WT})} = \left(\frac{Peak\ Area_{t=24}\ (^{14}N\ \alpha S_{WT})}{Peak\ Area_{t=24}(^{15}N\ \alpha S_{WT})} - \frac{Peak\ Area_{t=0}\ (^{14}N\ \alpha S_{WT})}{Peak\ Area_{t=0}(^{15}N\ \alpha S_{WT})}\right) \times 100$$

Proteomic analysis of acetylated αS standards

Each pure α-synuclein protein (20 µg) was denatured in 40 µL of 8 M urea in PBS, reduced by 5 mM of TCEP with 30 min incubation at 37 °C, alkylated by 15 mM of iodoacetamide (IAA) with 30 min incubation at room temperature in the dark. The solution was diluted to 2 M urea by 50 mM ammonium bicarbonate in H_2O, digested by trypsin (sequence grade, Promega) at 1:50 trypsin/protein ratio (*w*/*w*) with overnight (~12 h) incubation at 37 °C. The resulted peptide solution was acidified by formic acid at a final concentration of 5%. The peptide solution was desalted, resuspended in 0.1% formic acid at ~100 ng/µL. An LC-MS/MS system consisted of an Vanquish Neo UHPLC coupled to a Orbitrap Ascend (Thermo Scientific) was used for peptide analysis. Peptide samples were maintained at 7 °C on sample tray in LC. Separation of peptides was carried out on an Easy-Spray™ PepMap™ Neo nano-column (2 µm, C18, 75 µm X 150 mm) at room temperature with a mobile phase consisting of a linear gradient of A (0.1% FA in H_2O) and B (acetonitrile containing 0.1% FA) under the following conditions: 0 → 90 → 110 → 110.5 → 116 min, 0% → 28% → 36% → 98% → 98% B. The flow rate was 300 nL/min. 200 ng of each sample was injected for LCMS analysis in data-independent mode. The voltage applied to the nano-LC electrospray ionization source was 1.9 kV. The temperature of ion transfer tube (ITC) was set at 275 °C. Spectra were collected in a data-dependent acquisition mode such that each scan cycle (3 sec) involved a single high-resolution (120,000) full MS spectrum of parent ions (MS1 scan from m/z 350–2000) collected in the orbitrap. Parent ions assigned as peptide in charge states +2-6 with intensity higher than 2E4 were included for fragmentation. HCD-induced fragmentation (MS2) scans were recorded in orbitrap (scan from m/z 120–1500). Dynamic exclusion was set as repeat count of 1 within exclusion time of 20 s. All other parameters were left as default values. Relative ionization efficiency of the acetylated peptide over unmodified peptide was calculated based on the peptide intensity in acetylated αS compared to WT αS.

Quantitative analysis of αS Lys acetylation in human samples

Raw data were downloaded from previous works (50 raw files from PMID37814027(Zhang et al., 2023) and 7 raw files from PMID32461689(Schweighauser et al., 2020)). Data was searched using the Byonic software (v4.5.2) against a reverse-concatenated, nonredundant database of the human proteome. Cysteine residues were searched with a static modification for carbamidomethylation (+57.02146 Da). Methionine residues were searched with up to two differential modifications for oxidation (+15.9949 Da). Peptide is allowed to up to two acetylation (+42.0106 Da) on lysine residues. Peptides were required to have two tryptic terminals and up to two missed cleavages were allowed in the database search. The parent and daughter ion mass tolerances for a minimum envelope of three isotopic peaks was set to 10 and 20 ppm,

respectively. The false-positive rate was set at 1% or lower. The identified peptides from αS were listed in Supplementary Data 1 from both published datasets. MS1 peak area was used for acetylated and unmodified peptide quantification, only the raw files with identified acetylation sites of interest were included for analysis. Skyline software (v22.2.0.527) was used to export peak areas and data visualization.

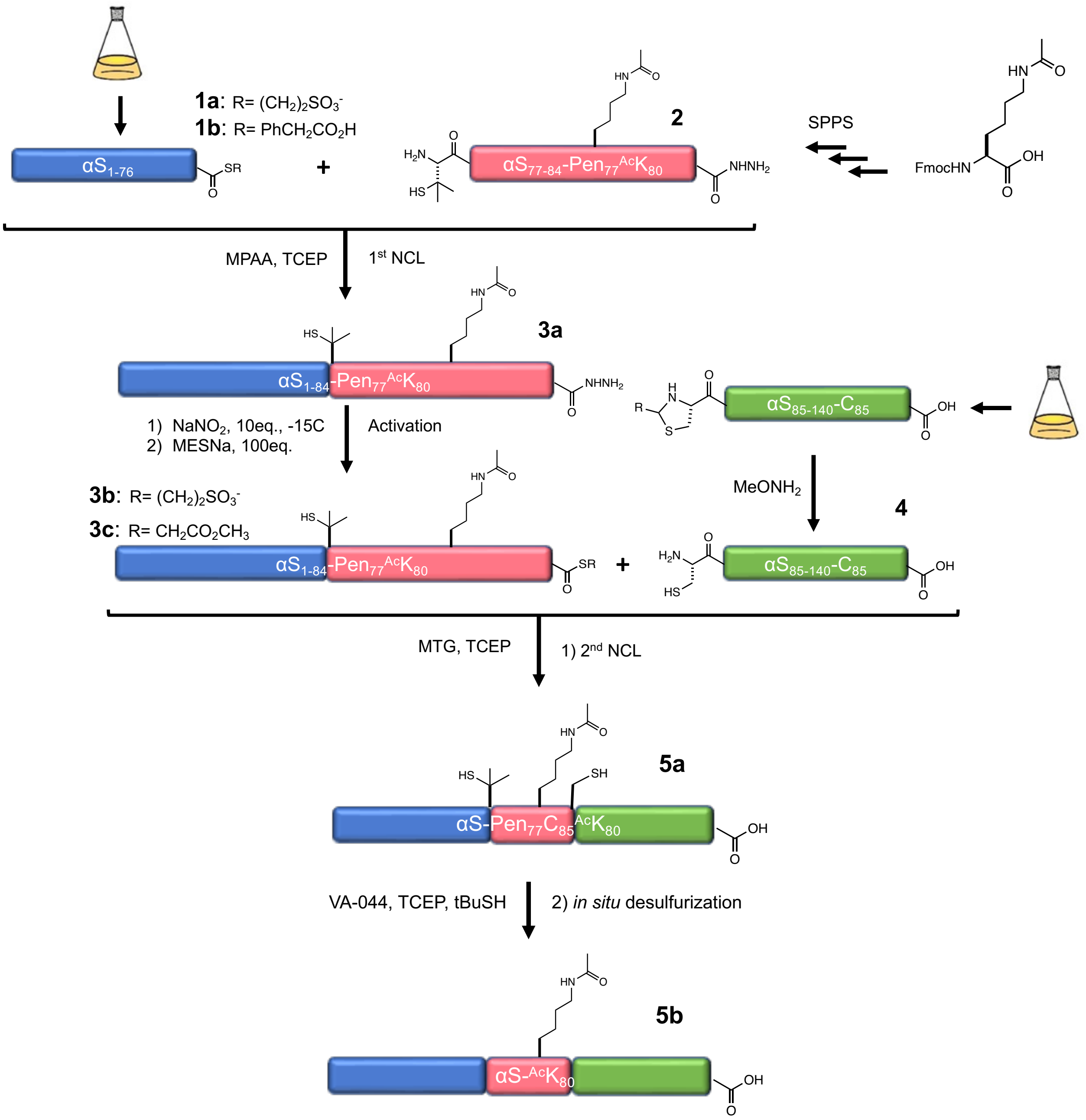

Scheme S1. Semi-synthesis scheme for creating αS-$^{Ac}K_{80}$.

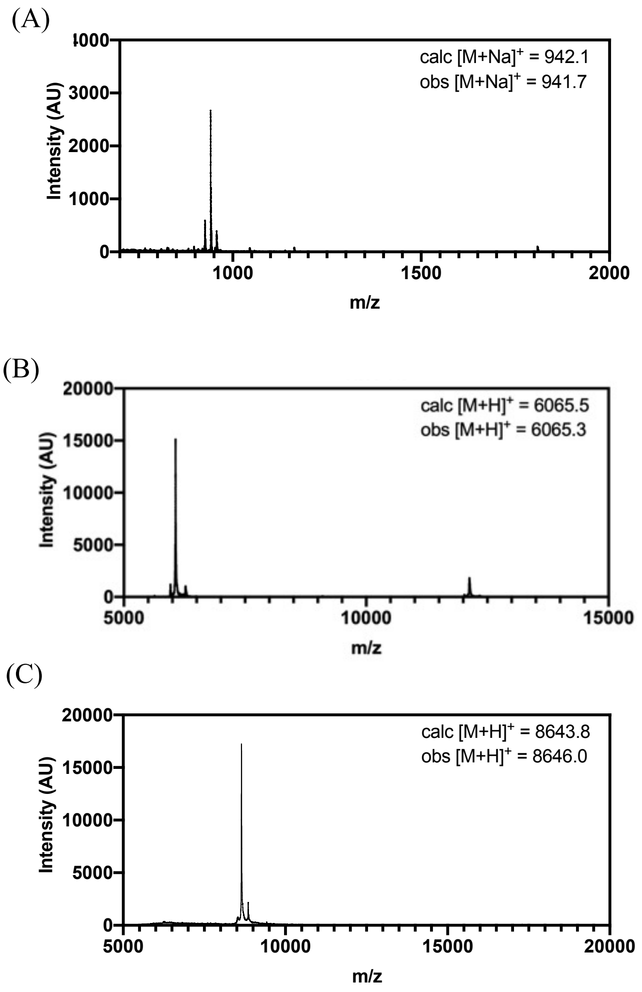

Figure S1. Protein semi-synthesis to incorporate ^{Ac}K at position 80**.** MALDI-TOF-MS characterization of (A) αS$_{77\text{-}84}$-Pen$_{77}$^{Ac}K$_{80}$-NHNH$_2$ (**2**), (B) αS$_{85\text{-}140}$-C$_{85}$ (**4**) and (C) αS$_{1\text{-}84}$-Pen$_{77}$^{Ac}K$_{80}$-MES (**3b**).

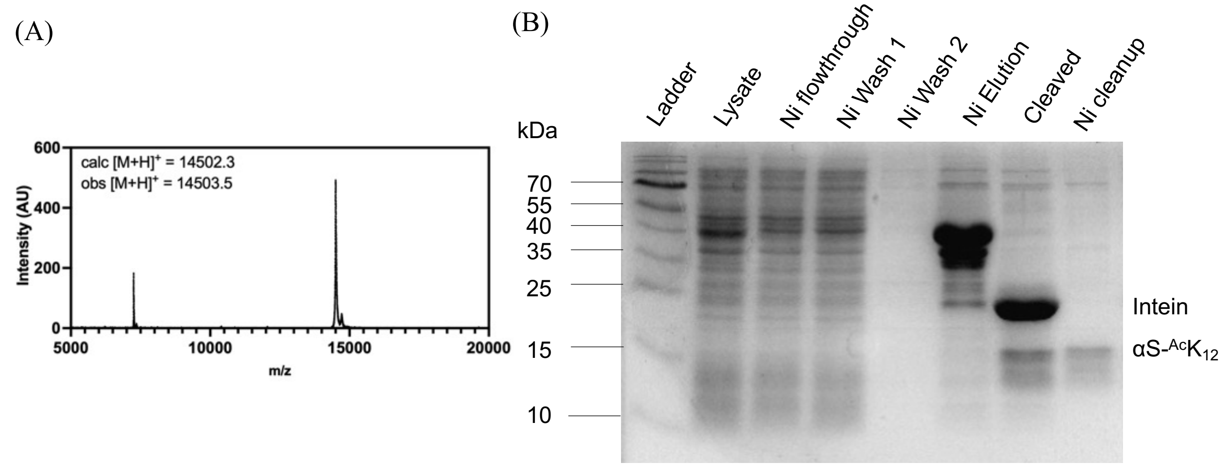

Figure S2. ncAA mutagenesis to incorporate acetyllysine at position 12. (A) MALDI-MS of purified product (B) SDS-PAGE with Coomassie staining to show affinity purification

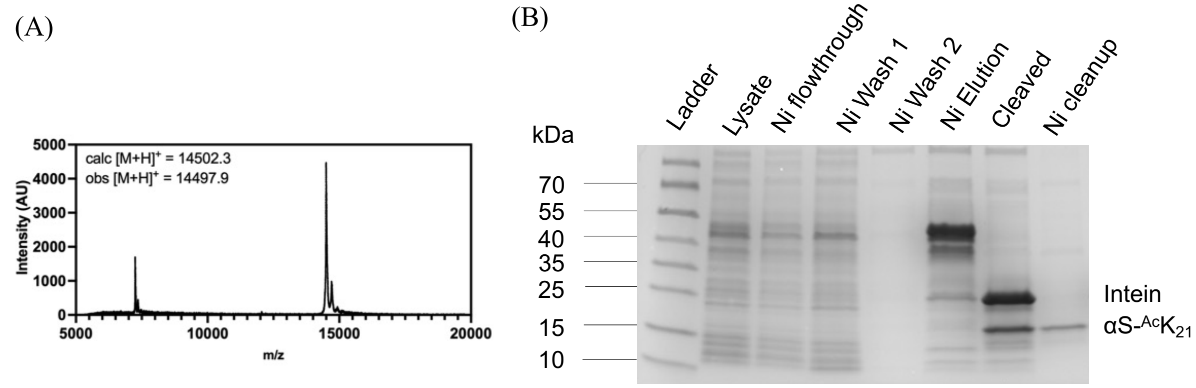

Figure S3. ncAA mutagenesis to incorporate acetyllysine at position 21. (A) MALDI-MS of purified product (B) SDS-PAGE with Coomassie staining to show affinity purification

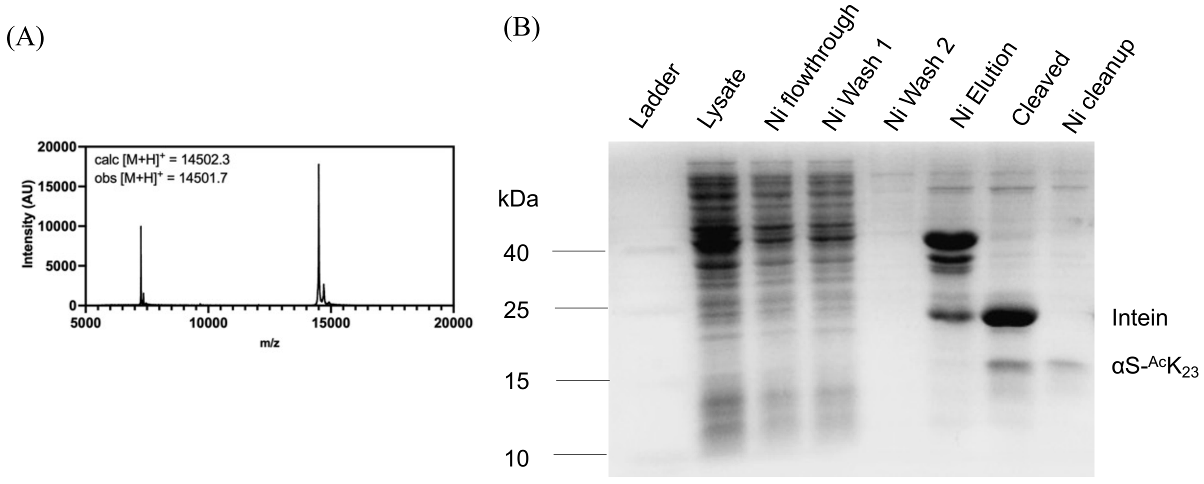

Figure S4. ncAA mutagenesis to incorporate acetyllysine at position 23. (A) MALDI-MS of purified product (B) SDS-PAGE with Coomassie staining to show affinity purification

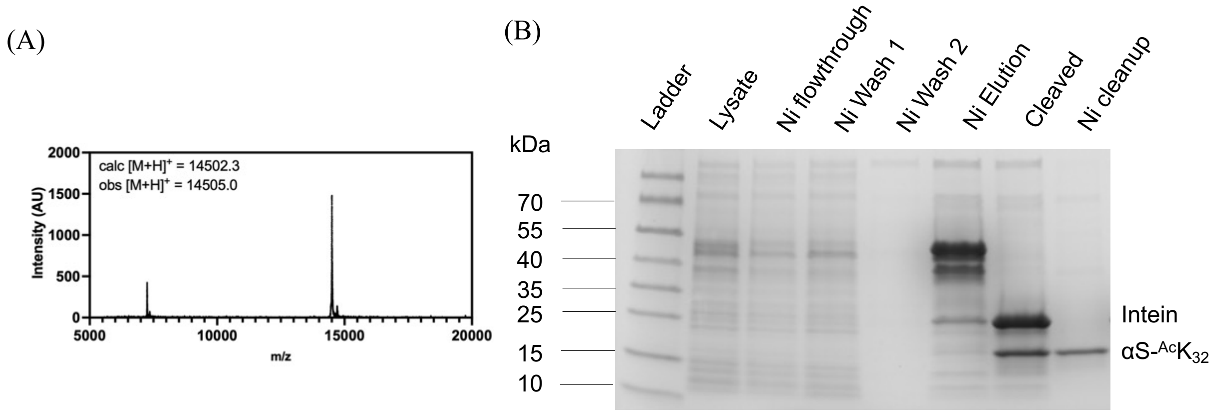

Figure S5. ncAA mutagenesis to incorporate acetyllysine at position 32. (A) MALDI-MS of purified product (B) SDS-PAGE with Coomassie staining to show affinity purification

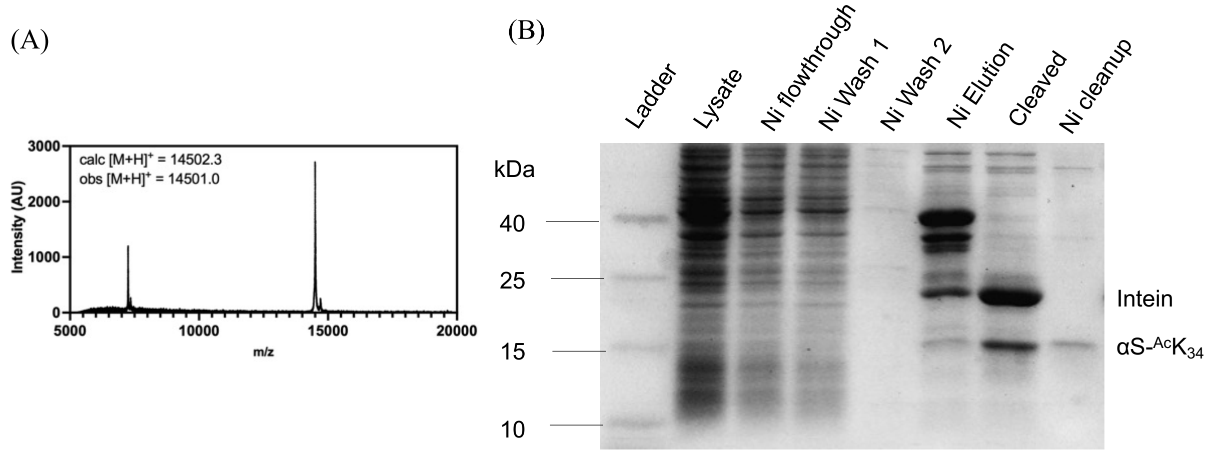

Figure S6. ncAA mutagenesis to incorporate acetyllysine at position 34. (A) MALDI-MS of purified product (B) SDS-PAGE with Coomassie staining to show affinity purification

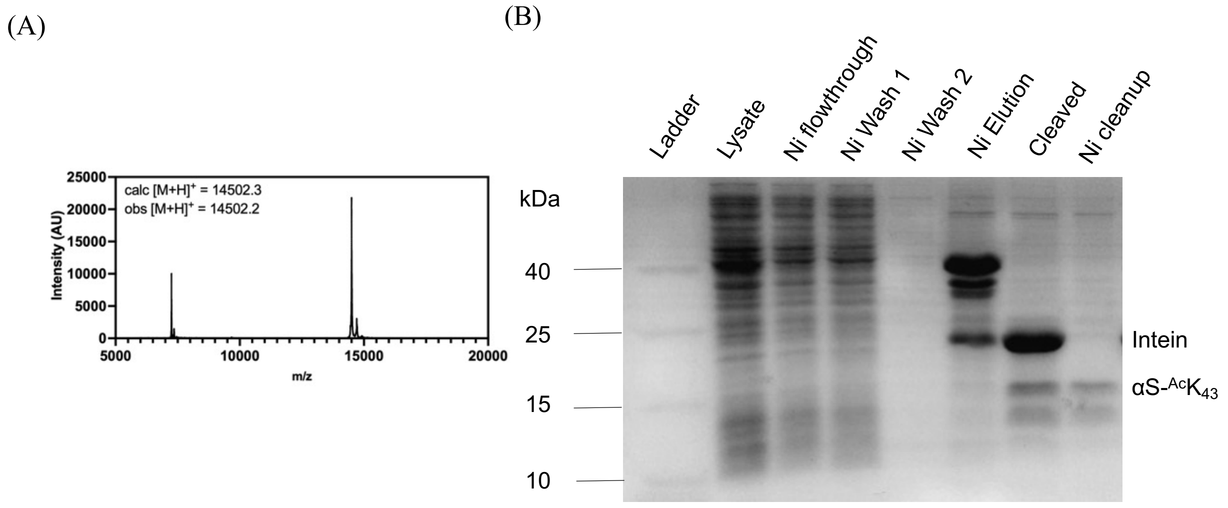

Figure S7. ncAA mutagenesis to incorporate acetyllysine at position 43. (A) MALDI-MS of purified product (B) SDS-PAGE with Coomassie staining to show affinity purification

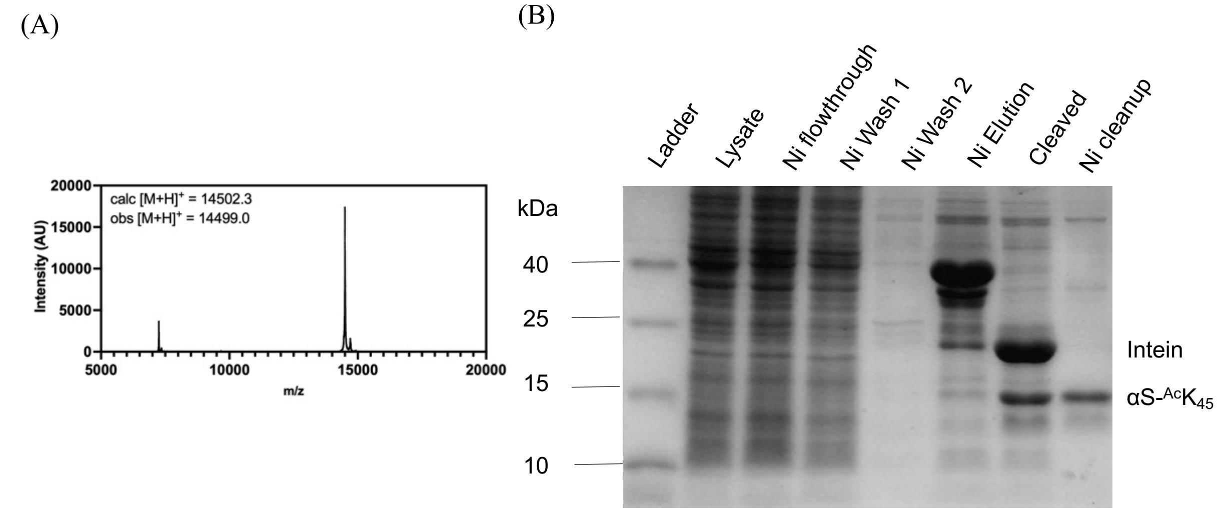

Figure S8. ncAA mutagenesis to incorporate acetyllysine at position 45. (A) MALDI-MS of purified product (B) SDS-PAGE with Coomassie staining to show affinity purification

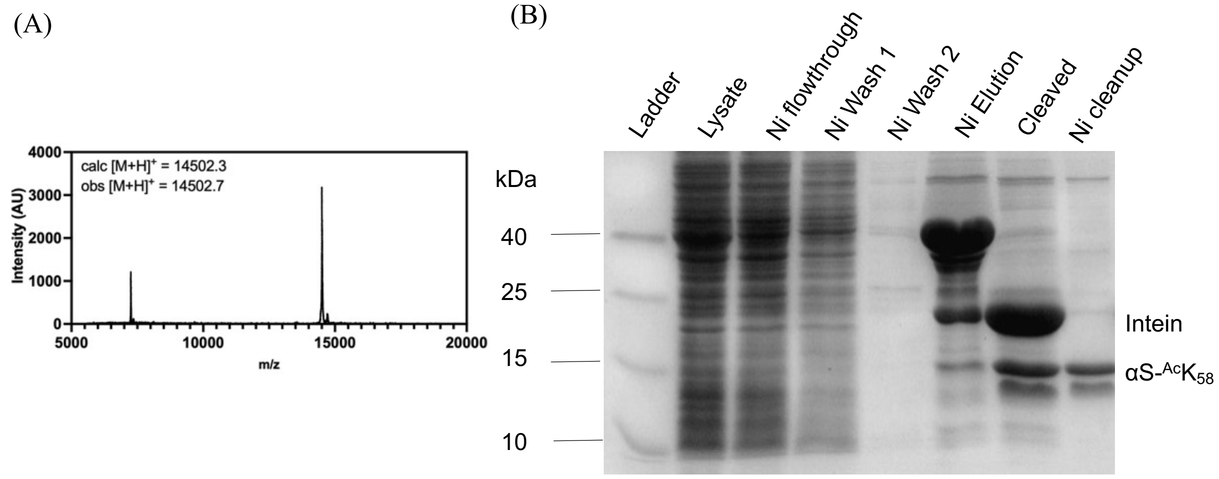

Figure S9. ncAA mutagenesis to incorporate acetyllysine at position 58. (A) MALDI-MS of purified product (B) SDS-PAGE with Coomassie staining to show affinity purification

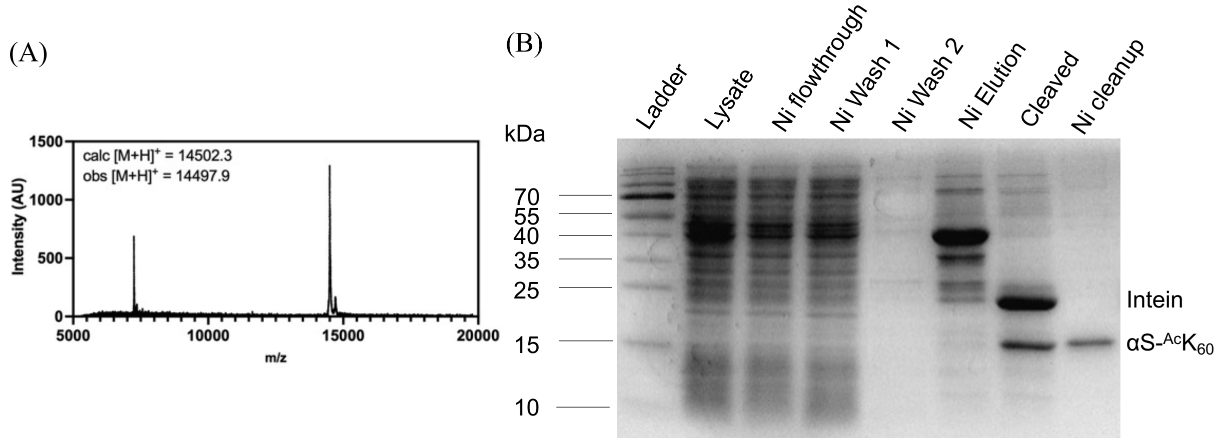

Figure S10. ncAA mutagenesis to incorporate acetyllysine at position 60. (A) MALDI-MS of purified product (B) SDS-PAGE with Coomassie staining to show affinity purification

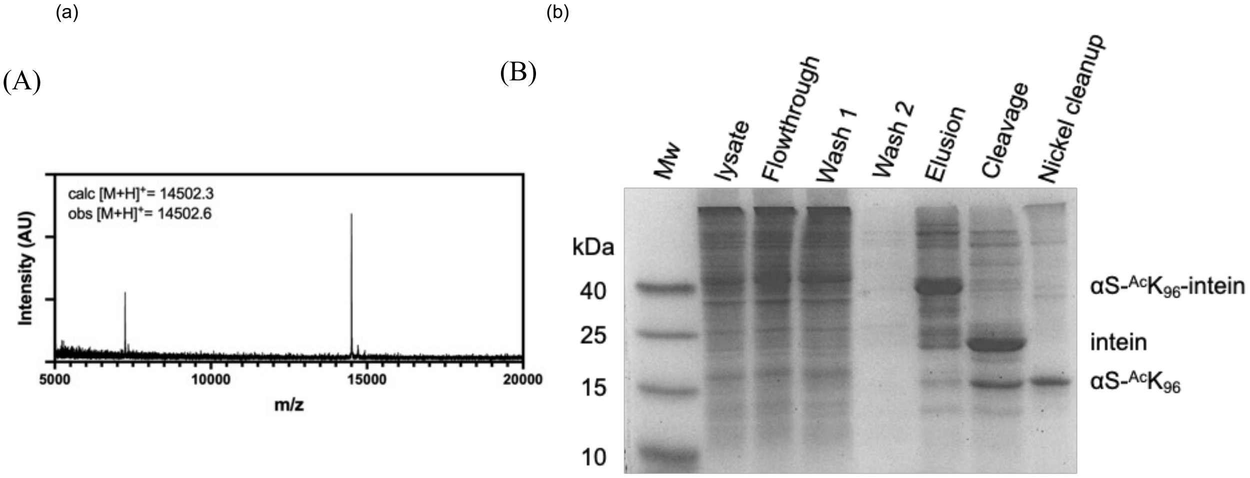

Figure S11. ncAA mutagenesis to incorporate acetyllysine at position 96. (A) MALDI-MS of purified product (B) SDS-PAGE with Coomassie staining to show affinity purification

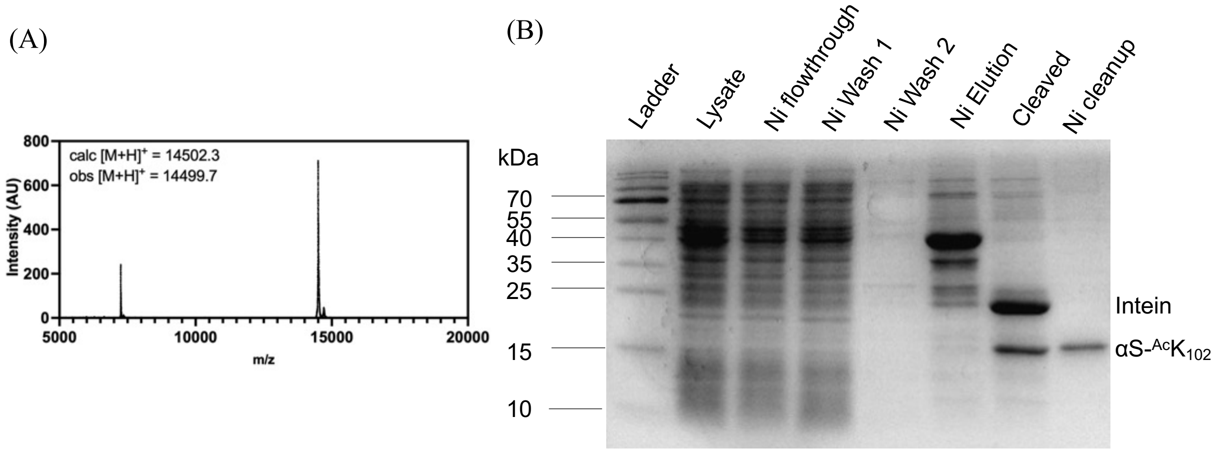

Figure S12. ncAA mutagenesis to incorporate acetyllysine at position 102. (A) MALDI-MS of purified product (B) SDS-PAGE with Coomassie staining to show affinity purification

(A) MDVFMKGLSK^{10} A**K**EGVVAAAE^{20} **K**T**K**QGVAEAA^{30} G**K**T**K**EGVLYV^{40} GS**K**T**K**EGVVH^{50} GVATVAE**K**T**K**^{60} EQVTNVGGAV^{70} VTGVTAVAQ**K**^{80} TVEGAGSIAA^{90} ATGFV**K**KDQL^{100} G**K**NEEGAPQE^{110} GILEDMPVDP^{120} DNEAYEMPSE^{130} EGYQDYEPEA^{140}

(B)

12: 0.23mg/L
TCAAAGGCCTAGGAGGGAGTT

21: 0.11mg/L
GCTGCTGAGTAGACCAAACAG

23: 0.35mg/L
GAGAAAACCTAGCAGGGTGTG

32: 0.23mg/L
GCAGCAGGATAGACAAAAGAG

34: 0.76mg/L
GGAAAGACATAGGAGGGTGTT

43: 0.46mg/L
GTAGGCTCCTAGACCAAGGAG

45: 1.03mg/L
TCCAAAACCTAGGAGGGAGTG

58: 1.47mg/L
GTGGCTGAGTAGACCAAAGAG

60: 0.22mg/L
GAGAAGACCTAGGAGCAAGTG

80: 0.65mg/L
GTAGCCCAGTAGACAGTGGAG

96: 0.20mg/L
GGCTTTGTCTAGAAGGACCAG

102: 0.38mg/L
CAGTTGGGCTAGAATGAAGAA

Figure S13. Local sequential contexts of amber codon suppression. (A) Amino acid sequence of αS with acetyl lysine incorporation sites bolded and underlined, (B) Expression yield per liter of *E. coli* culture for each acetylated construct. Sites 21 and 58 have very similar local sequence context yet gave very different suppression yields.

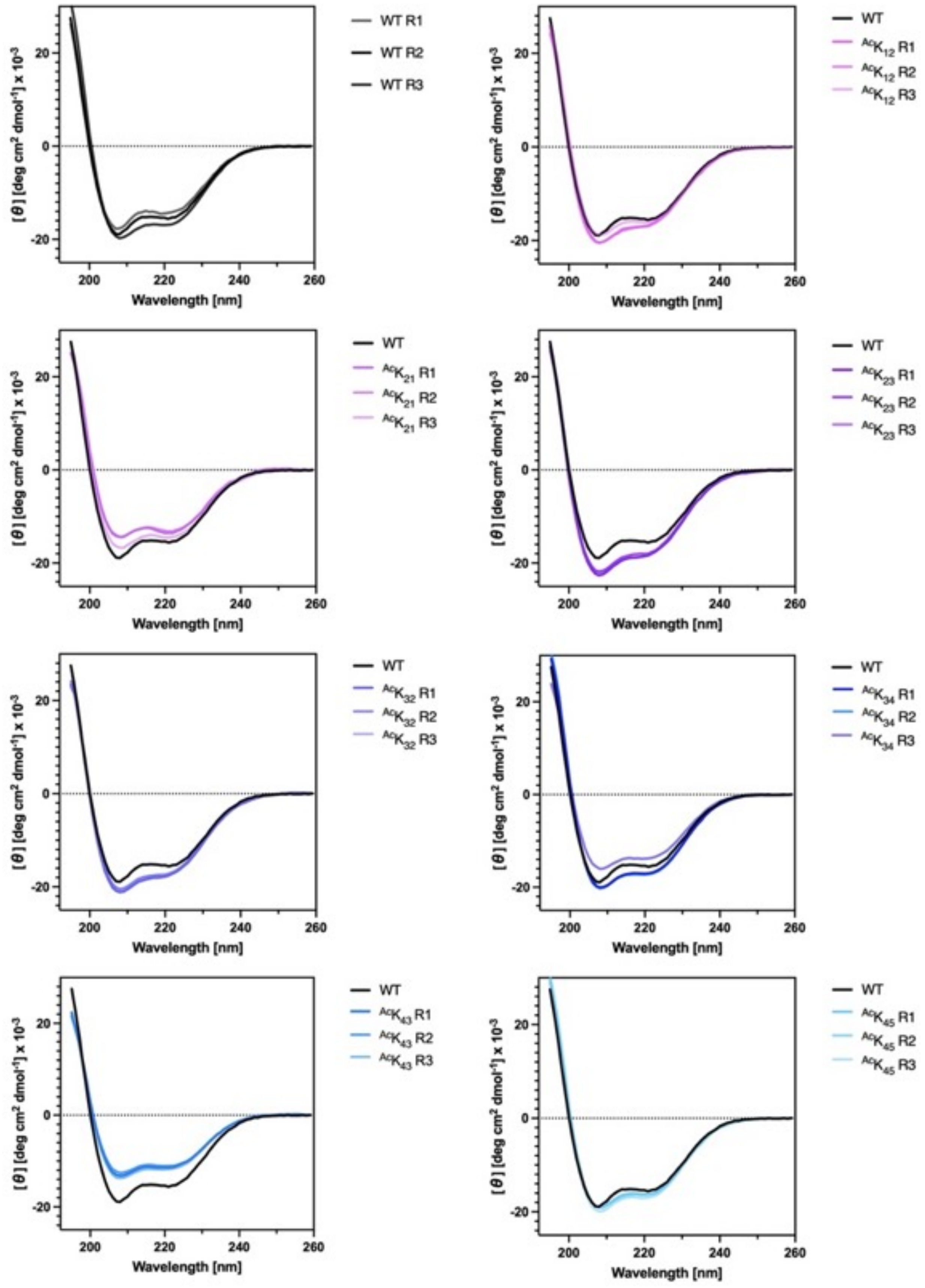
WT R1
WT R2
WT R3
WT
$^{Ac}K_{12}$ R1
$^{Ac}K_{12}$ R2
$^{Ac}K_{12}$ R3
WT
$^{Ac}K_{21}$ R1
$^{Ac}K_{21}$ R2
$^{Ac}K_{21}$ R3
WT
$^{Ac}K_{23}$ R1
$^{Ac}K_{23}$ R2
$^{Ac}K_{23}$ R3
WT
$^{Ac}K_{32}$ R1
$^{Ac}K_{32}$ R2
$^{Ac}K_{32}$ R3
WT
$^{Ac}K_{34}$ R1
$^{Ac}K_{34}$ R2
$^{Ac}K_{34}$ R3
WT
$^{Ac}K_{43}$ R1
$^{Ac}K_{43}$ R2
$^{Ac}K_{43}$ R3
WT
$^{Ac}K_{45}$ R1
$^{Ac}K_{45}$ R2
$^{Ac}K_{45}$ R3
$[\theta]$ [deg cm^2 dmol^{-1}] x 10^{-3}
Wavelength [nm]

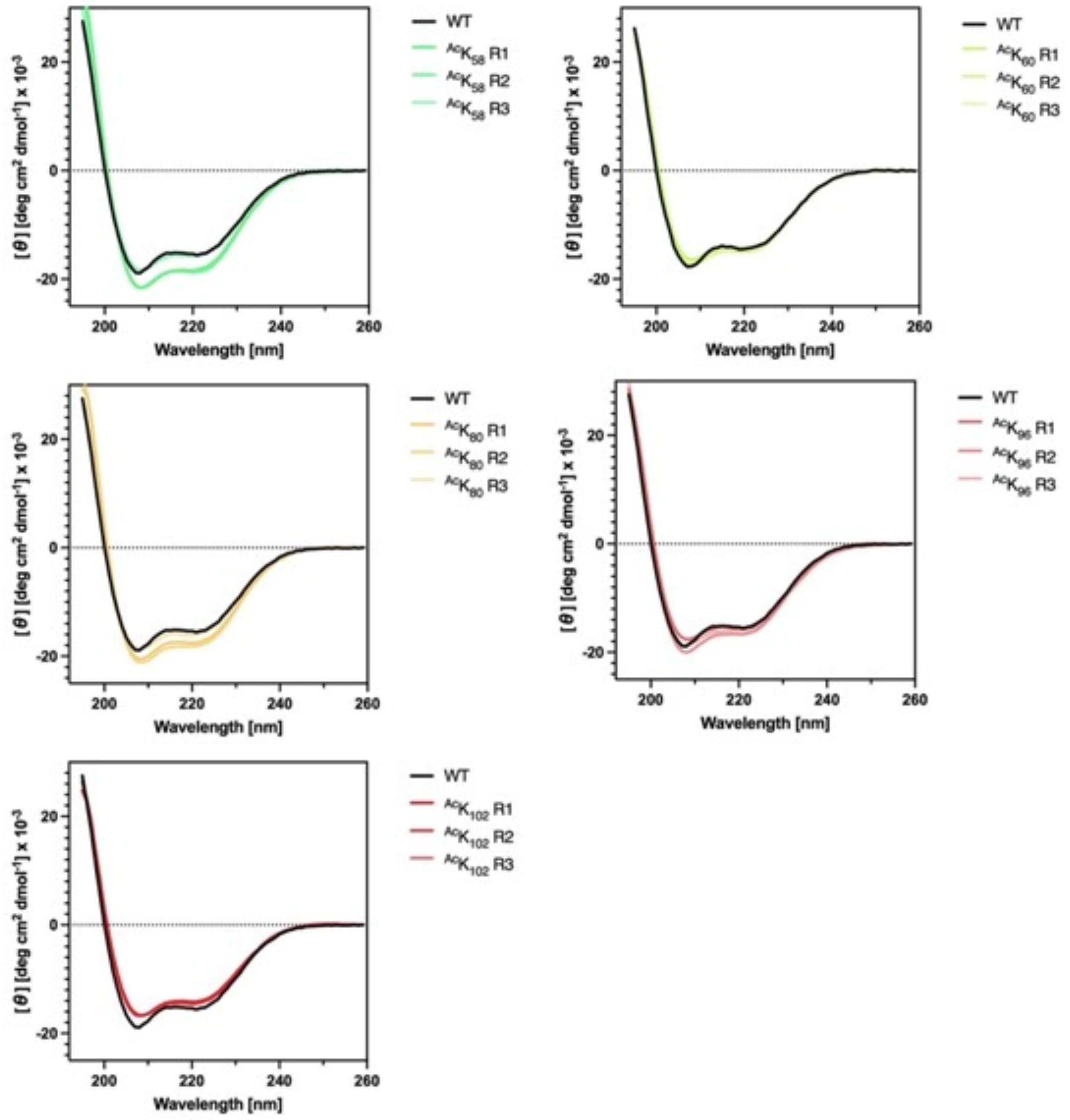

Figure S14. Individual CD wavelength scans for αS-^{Ac}K constructs.

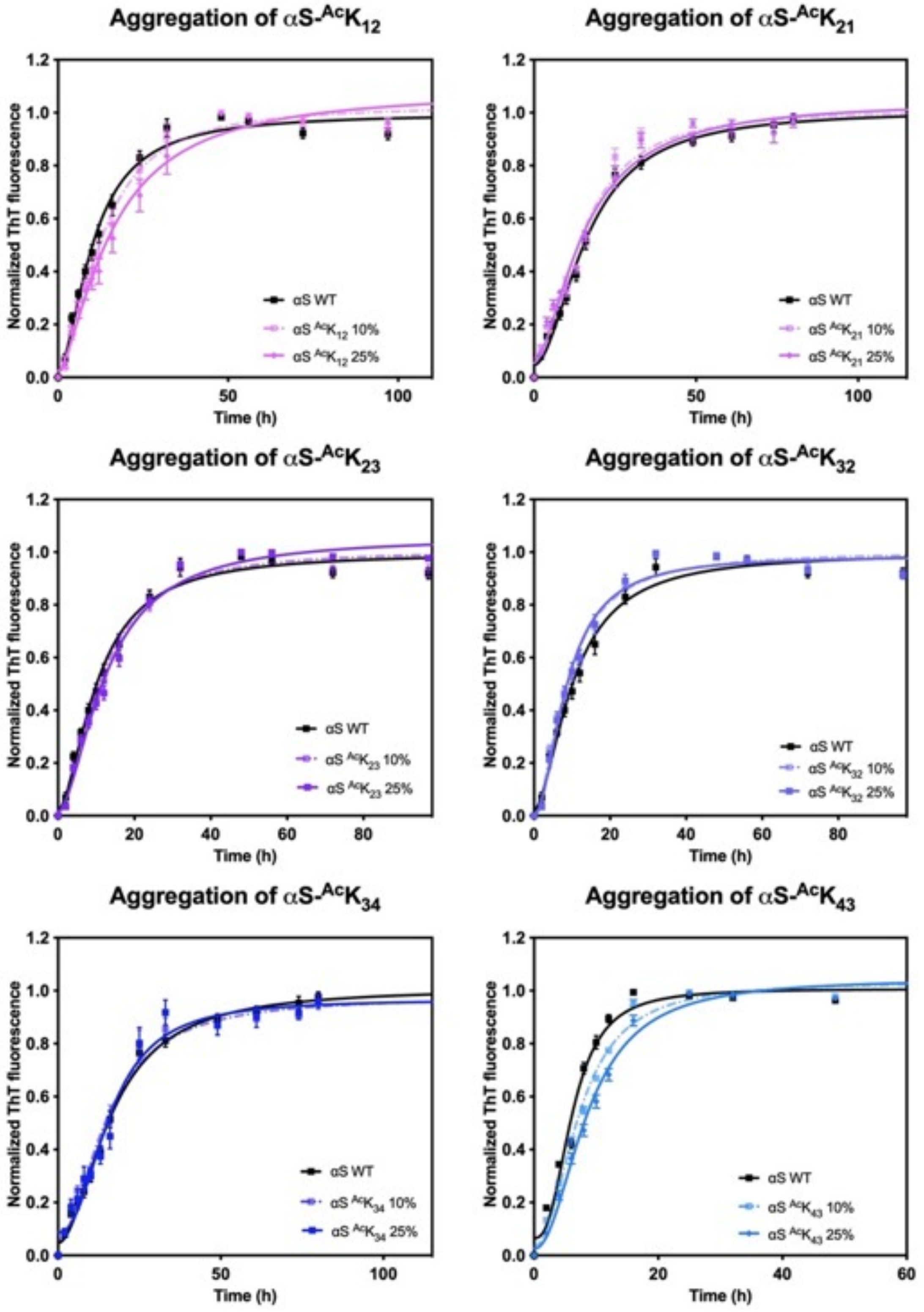

Figure S15a. Aggregation kinetics curves for each αS-^{Ac}K construct.

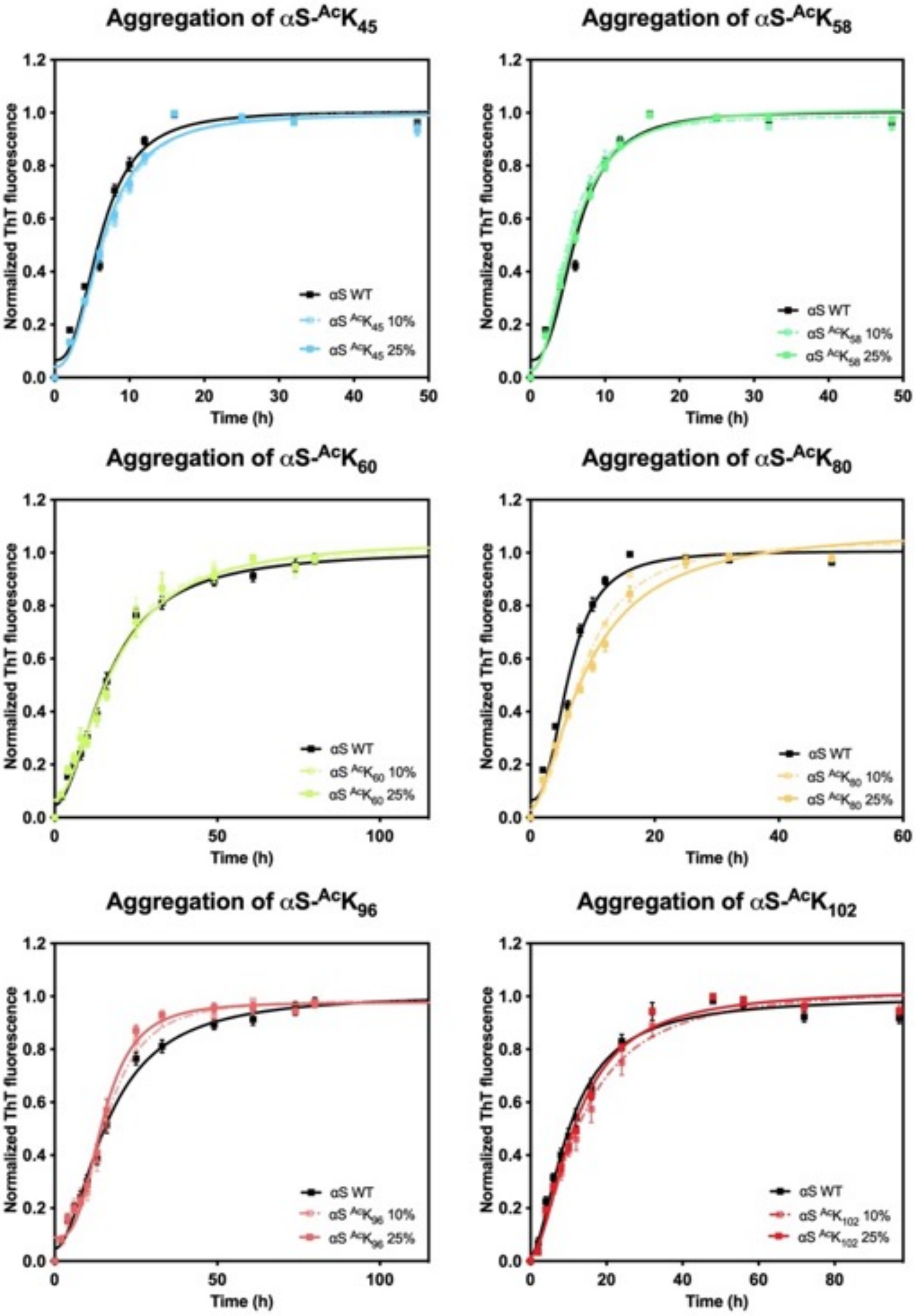

Figure S15b. Aggregation kinetics curves for each αS-^{Ac}K construct.

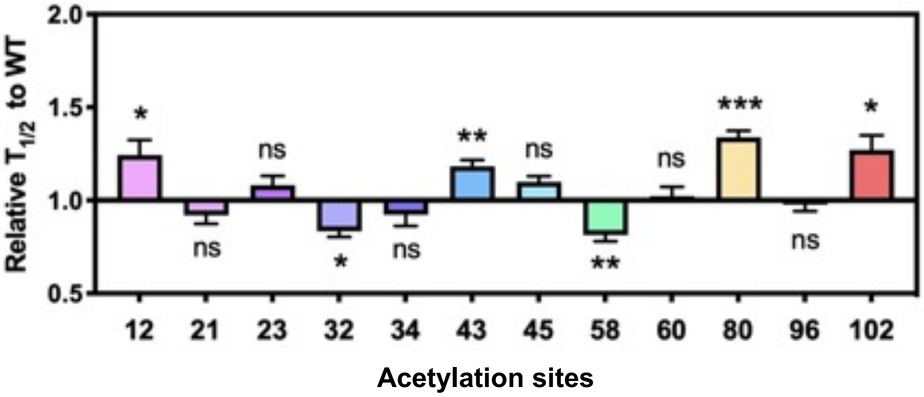

Figure S16. Effects of 10% αS-^{Ac}K on aggregation kinetics. The time to reach 50% fibrilization ($T_{1/2}$) for each condition was normalized to that of a 100% WT aggregation. Seeded aggregation was performed with αS monomers where acetylated αS was mixed with αS WT at 10%:90% ratio. Mean, with standard error of six replicates.

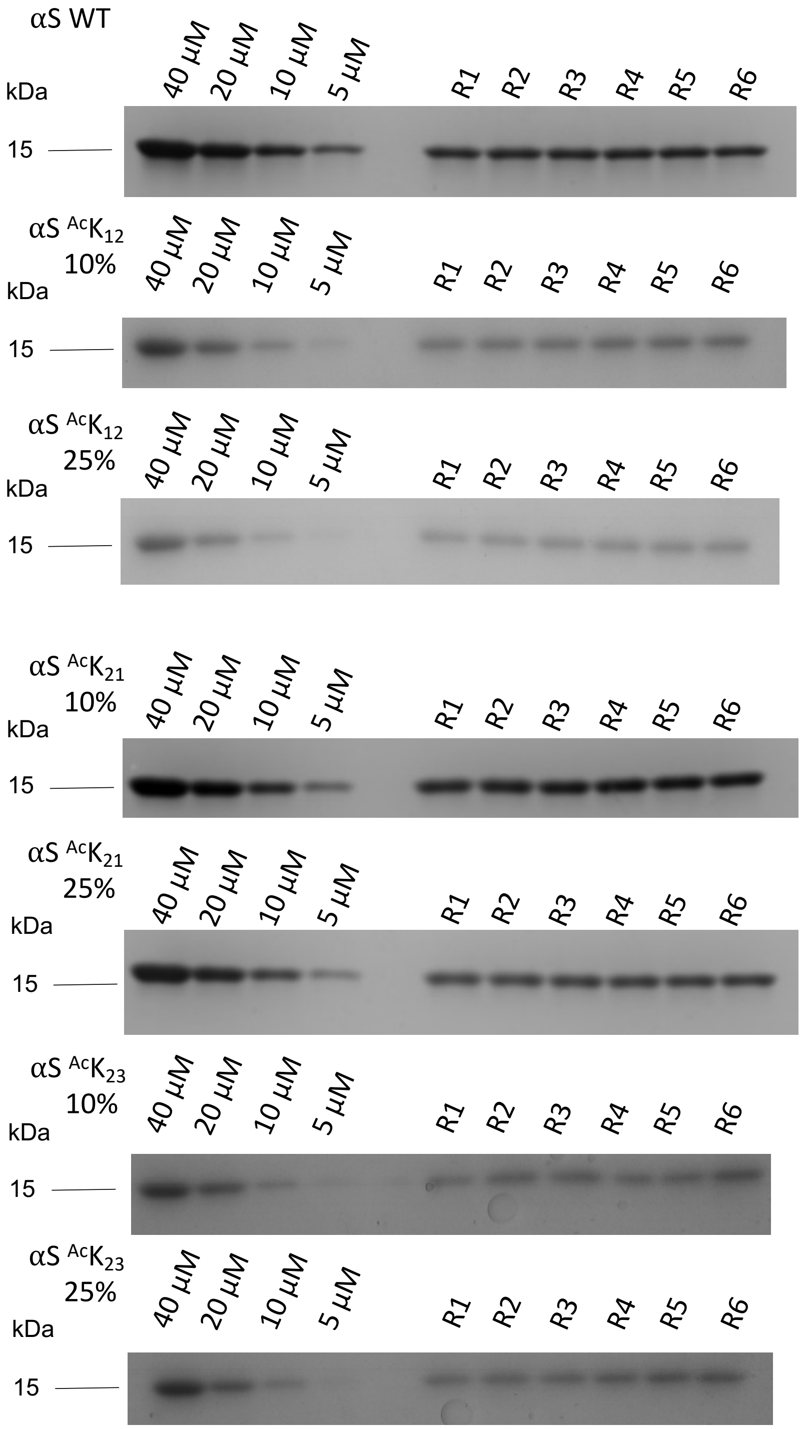

Figure S17a. Primary SDS-PAGE gels for quantifying monomer incorporations of αS-^{Ac}K constructs. Six replicates shown (R1-6).

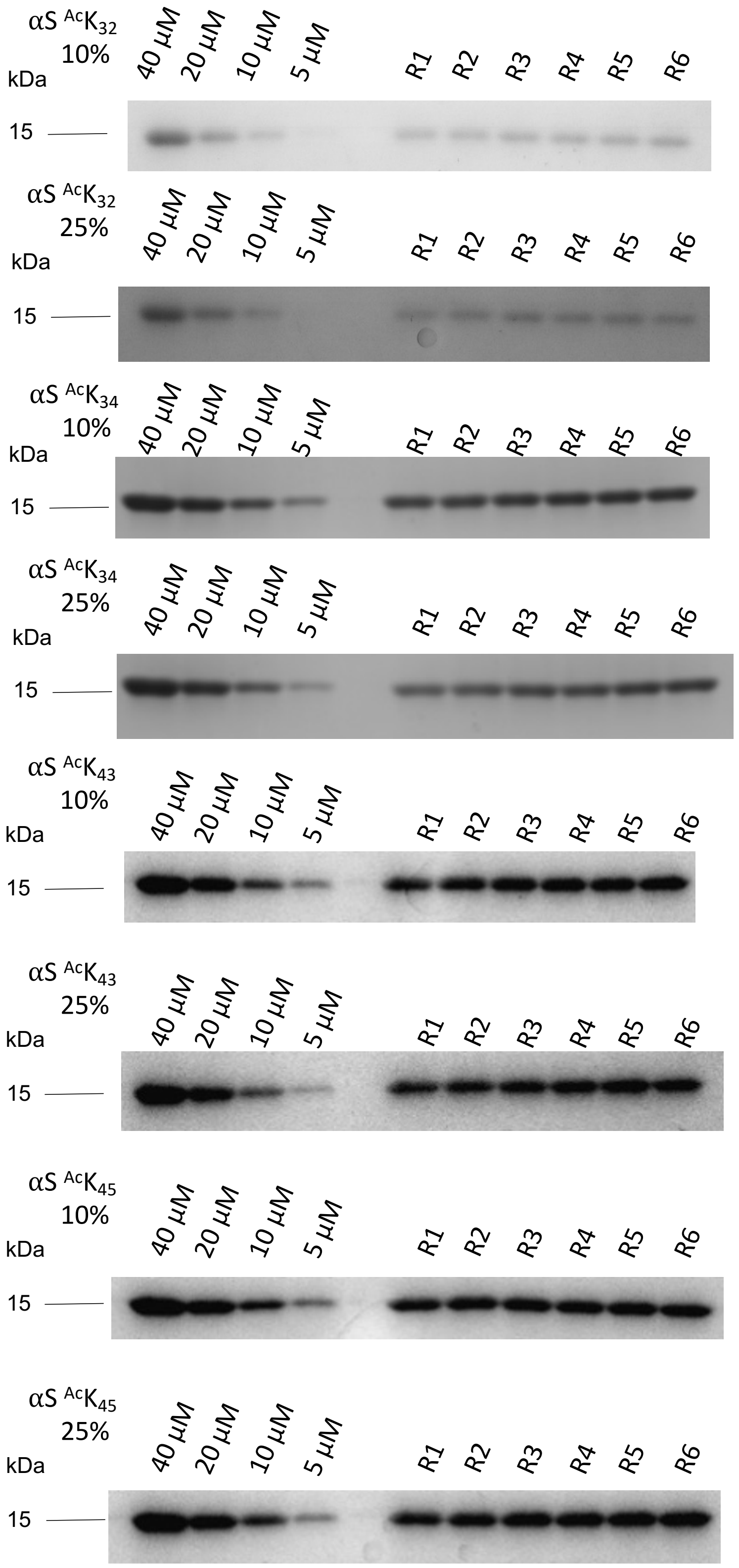

Figure S17b. Primary SDS-PAGE gels for quantifying monomer incorporations of αS-^{Ac}K constructs. Six replicates shown (R1-6)

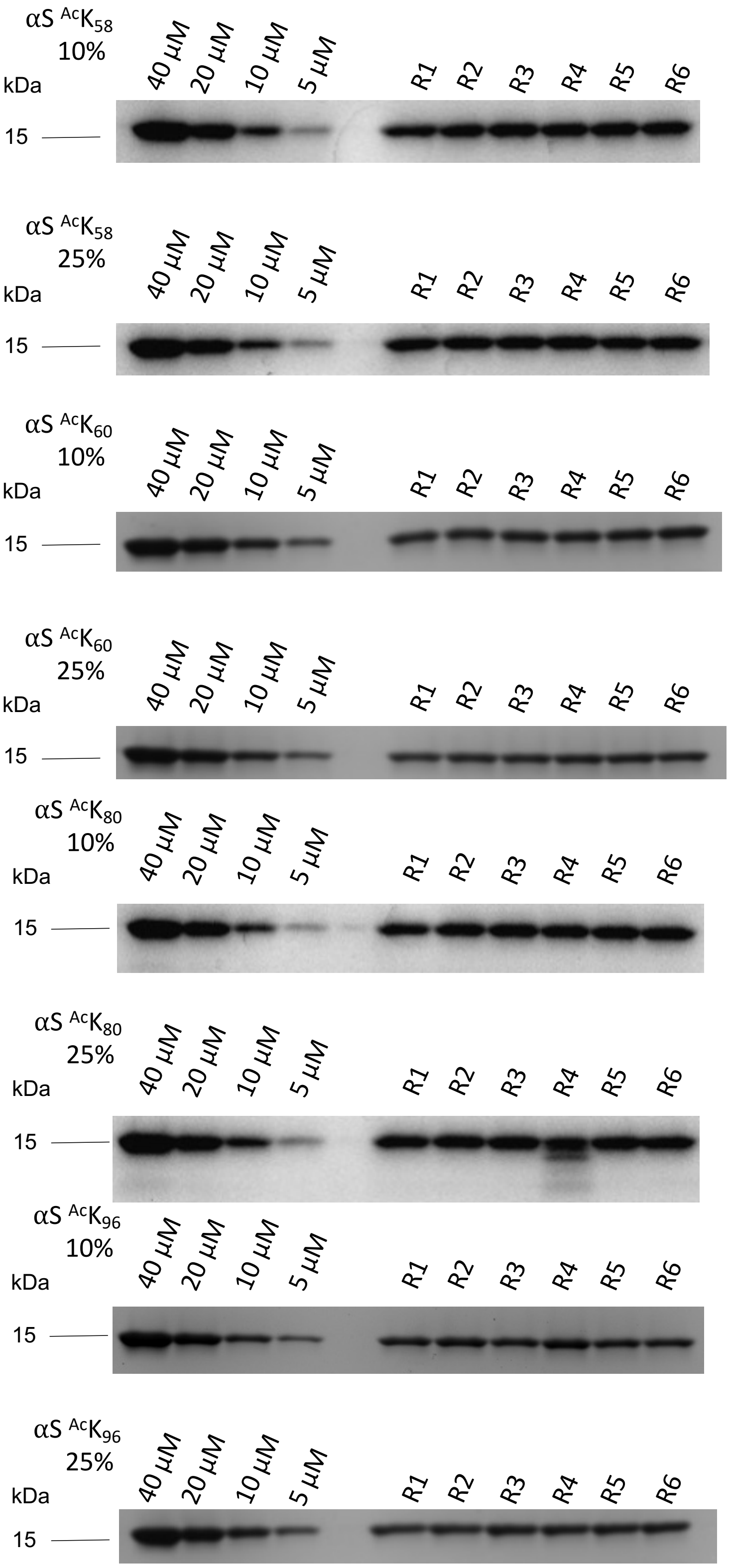

Figure S17c. Primary SDS-PAGE gels for quantifying monomer incorporations of αS-^{Ac}K constructs. Six replicates shown (R1-6).

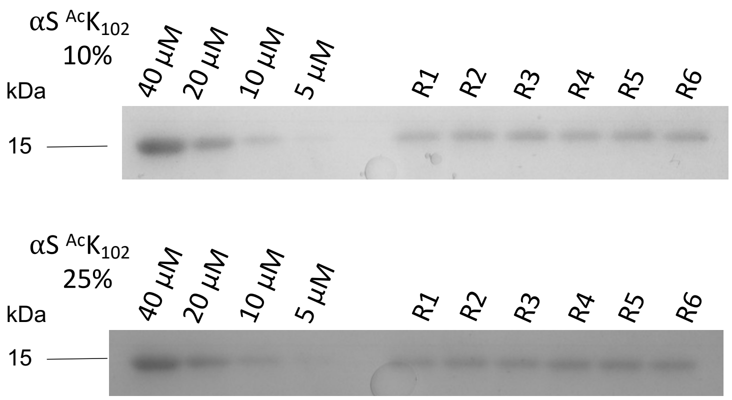

Figure S17d. Primary SDS-PAGE gels for quantifying monomer incorporations of αS-^{Ac}K constructs. Six replicates shown (R1-6).

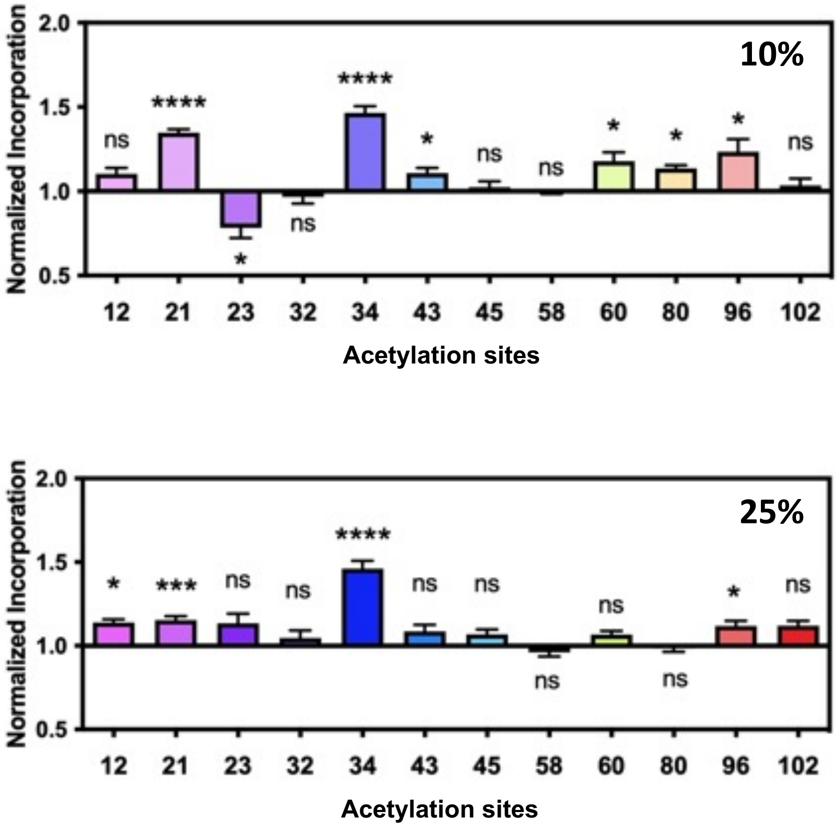

Figure S18. Effects of αS-^{Ac}K on total monomer incorporation. Monomers incorporated into fibrils were quantified by SDS-PAGE gels and normalized to WT values. Mean with standard error, R=6

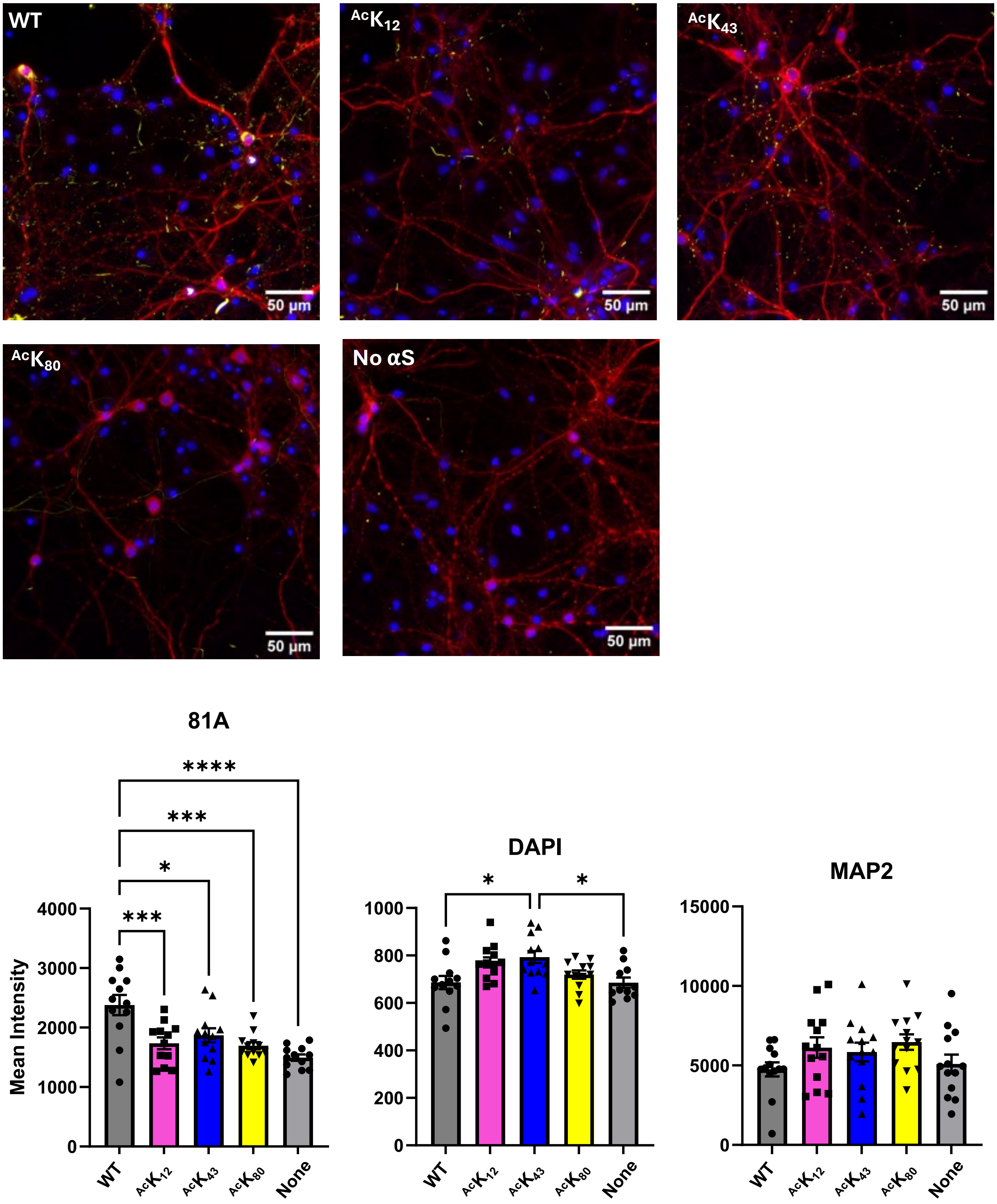

Figure S19. Neuron imaging data Top: Representative images of neuron cultures with additional stains. Yellow = 81A (anti-pS129), Blue = DAPI, Red = MAP2 (Larger fields of view shown than in main text). Bottom: Fluorescence intensity from 81A, DAPI, and MAP2 channels. * = 0.01 < p-value < 0.05; *** = 0.001 < p-value < 0.0001; **** = 0.00001 < p-value < 0.0001

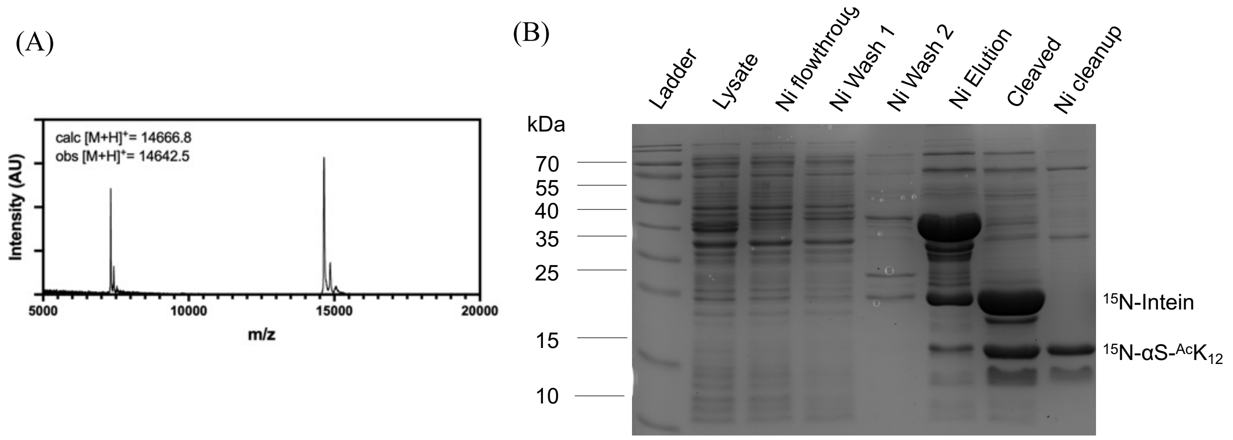

Figure S20. Recombinant ^{15}N- αS-^{Ac}K$_{12}$ (A) MALDI-MS of purified product (B) SDS-PAGE with Coomassie staining to show affinity purification.

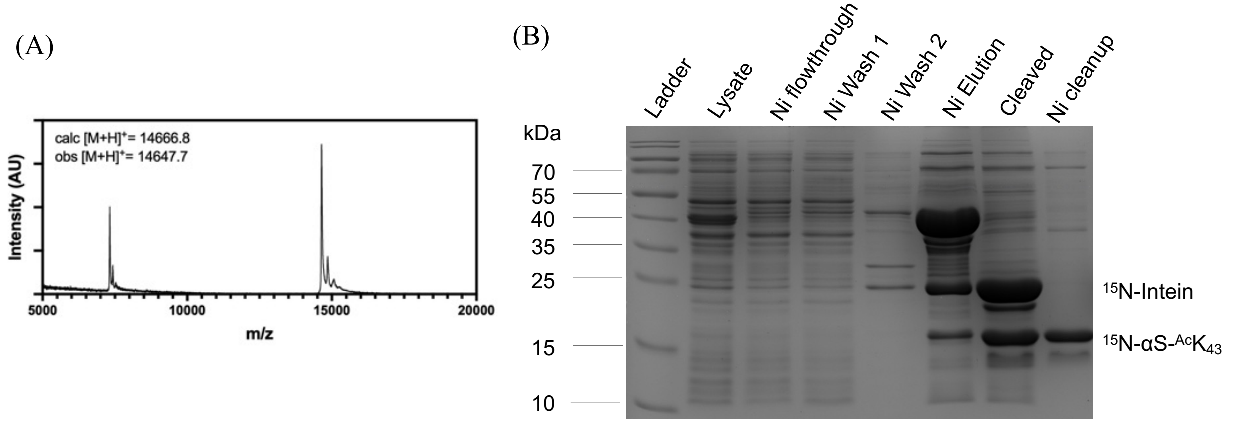

Figure S21. Recombinant ^{15}N- αS-^{Ac}K$_{43}$ (A) MALDI-MS of purified product (B) SDS-PAGE with Coomassie staining to show affinity purification.

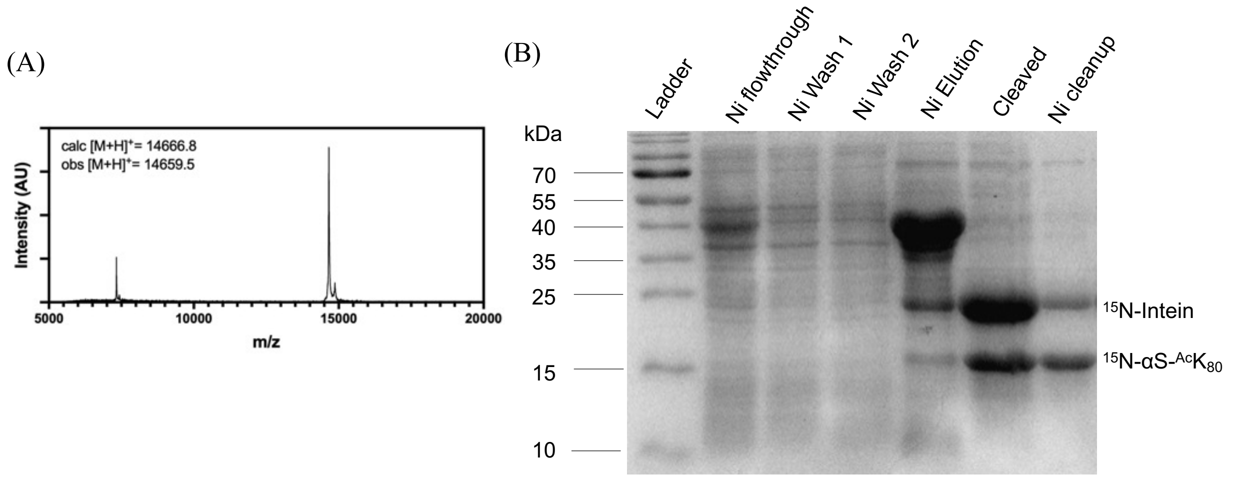

Figure S22. Recombinant ^{15}N- αS-^{Ac}K$_{80}$ (A) MALDI-MS of purified product (B) SDS-PAGE with Coomassie staining to show affinity purification.

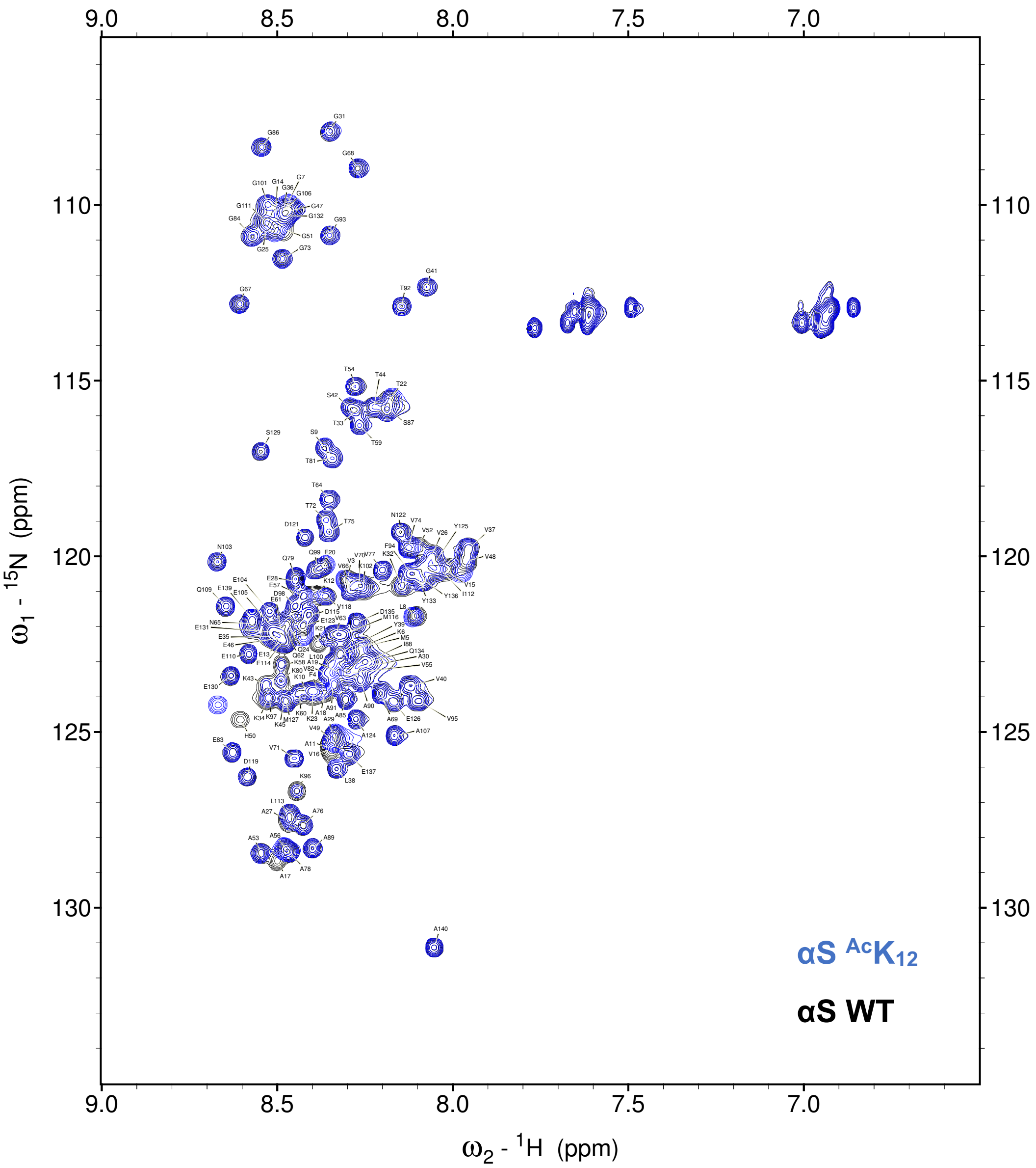

9.0
8.5
8.0
7.5
7.0
110
115
120
125
130
ω1 - 15N (ppm)
ω2 - 1H (ppm)
αS AcK12
αS WT

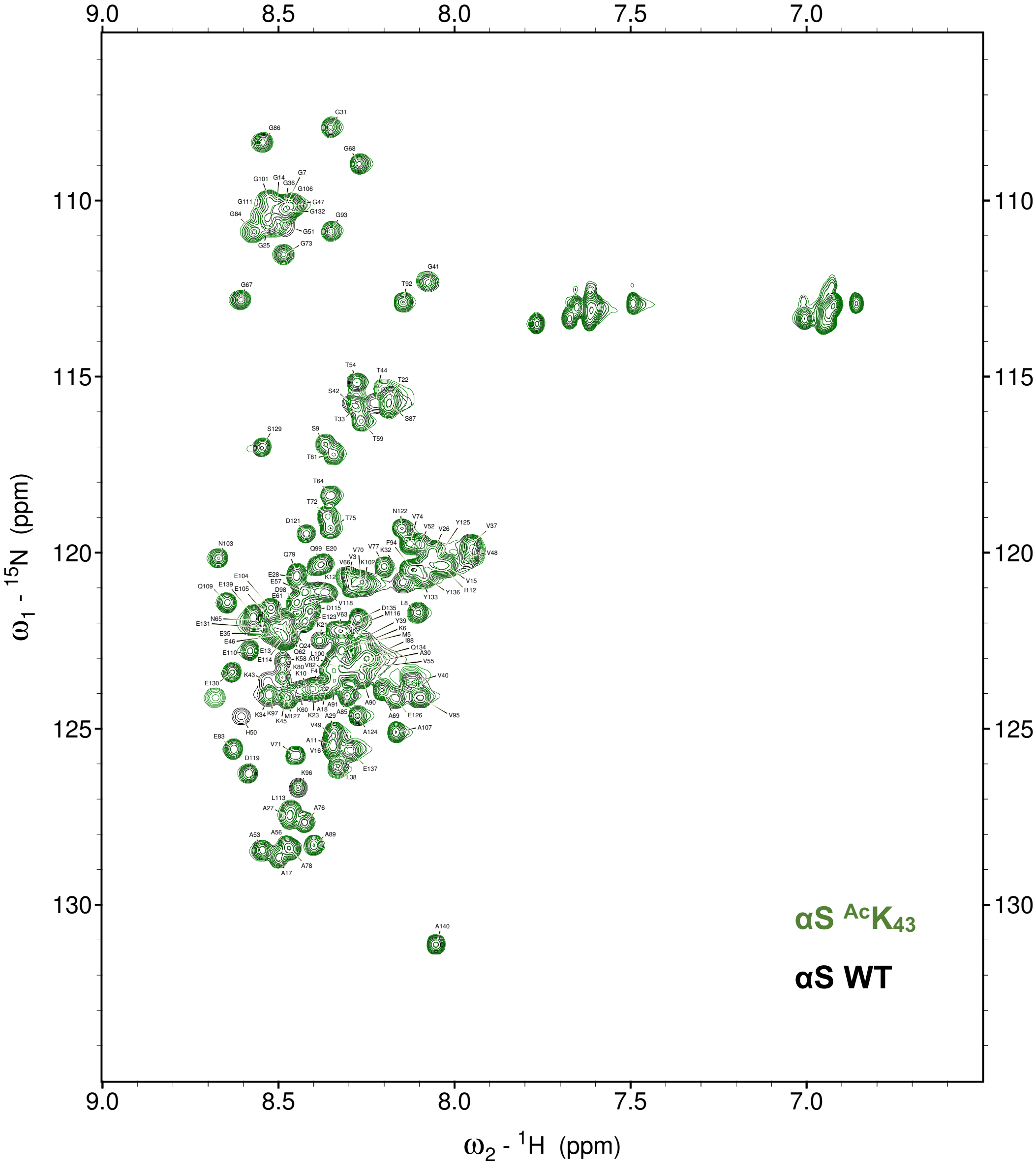

9.0
8.5
8.0
7.5
7.0
110
115
120
125
130
ω1 - 15N (ppm)
ω2 - 1H (ppm)
αS AcK43
αS WT

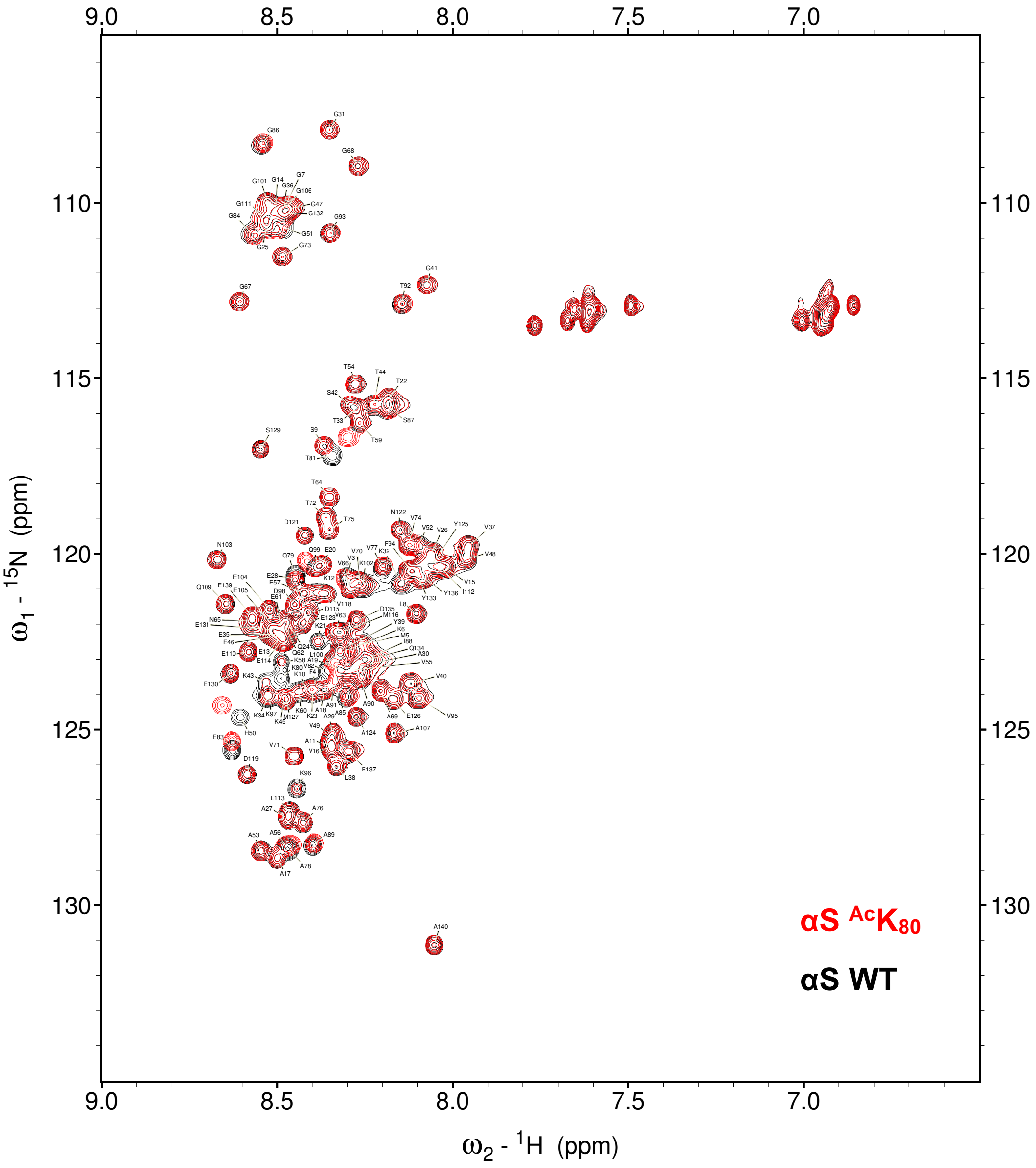

Figure S23. HSQC spectra acquired for αS-$^{Ac}K_{12}$, $^{Ac}K_{43}$ or $^{Ac}K_{80}$ in buffers. The spectra of each were overlayed with the spectra of αS WT.

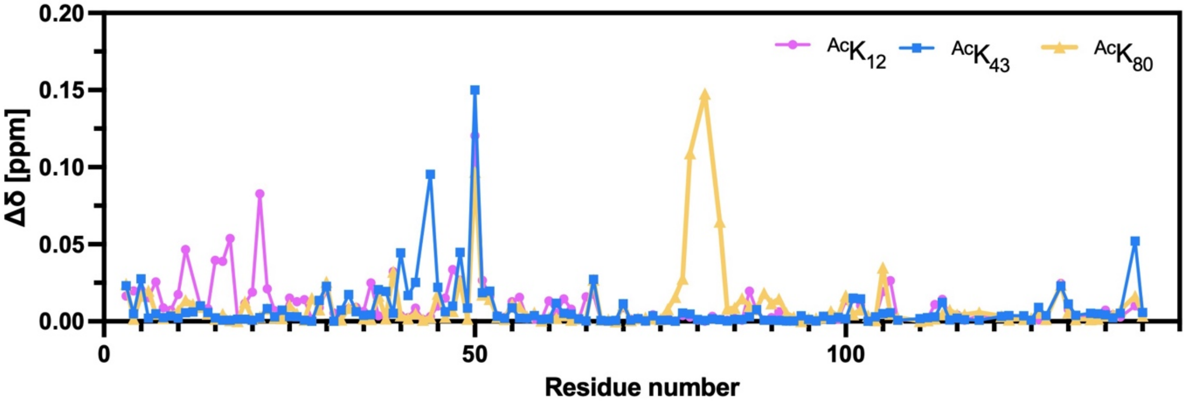

Figure S24. Chemical shift perturbation (Δδ; CSP) from αS-WT calculated at each residue of αS-$^{Ac}K_{12}$, αS-$^{Ac}K_{43}$ or αS-$^{Ac}K_{80}$

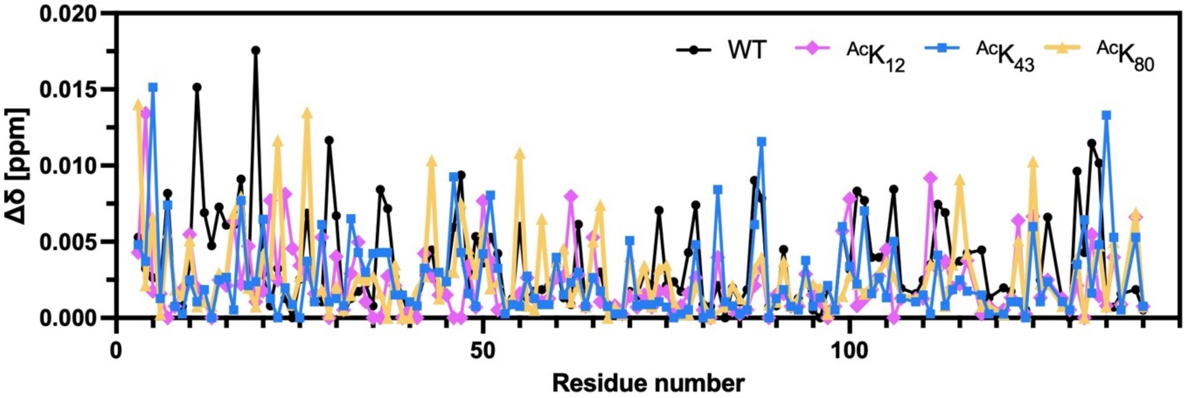

Figure S25. Chemical shift perturbation (Δδ; CSP) calculated at each residue of vesicle-bound αS-WT, αS-$^{Ac}K_{43}$ or αS-$^{Ac}K_{80}$. CSP was calculated by comparing spectra acquired for vesicle-bound state and spectra of free state.

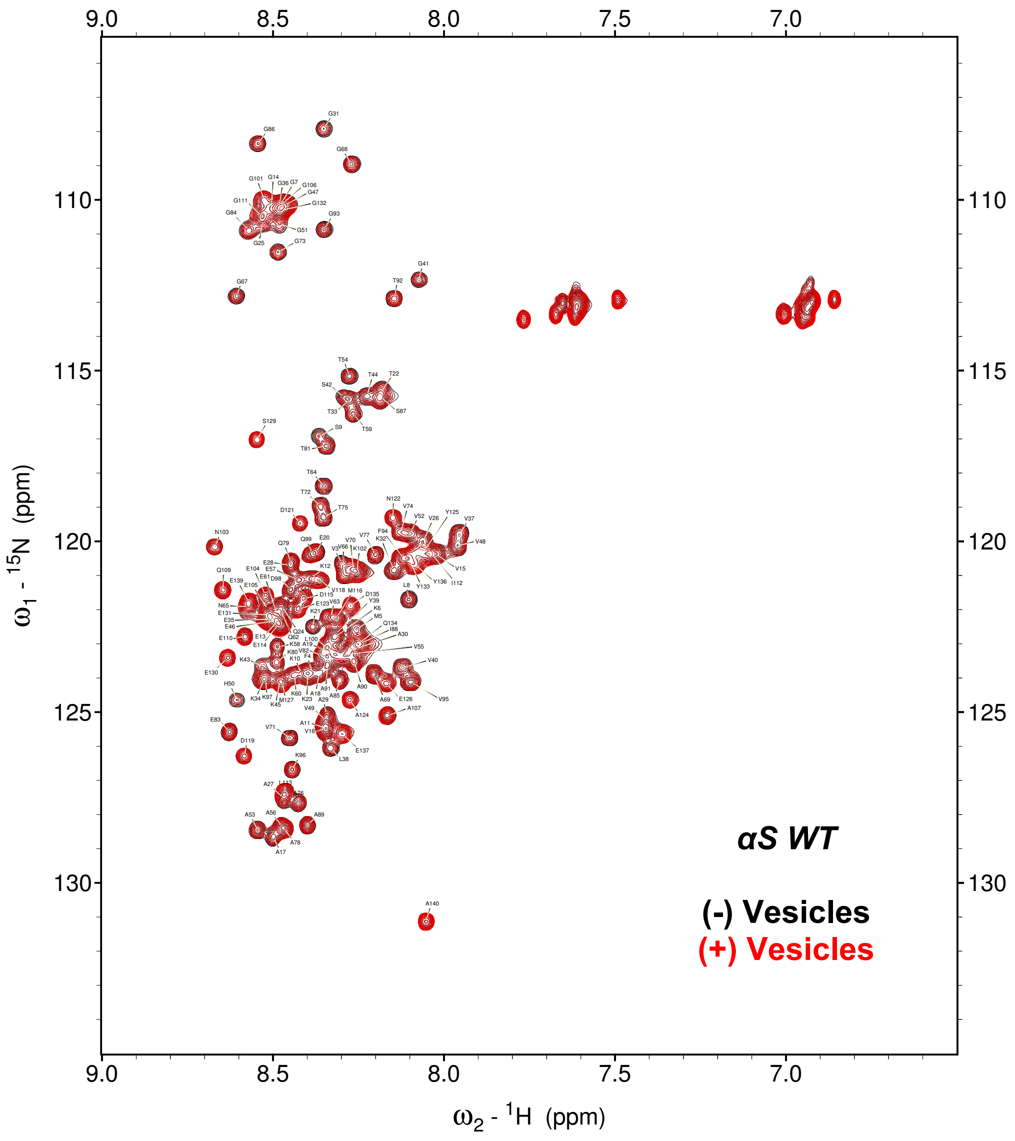
9.0
8.5
8.0
7.5
7.0
110
115
120
125
130
ω_1 - ^{15}N (ppm)
ω_2 - ^{1}H (ppm)
αS WT
(-) Vesicles
(+) Vesicles

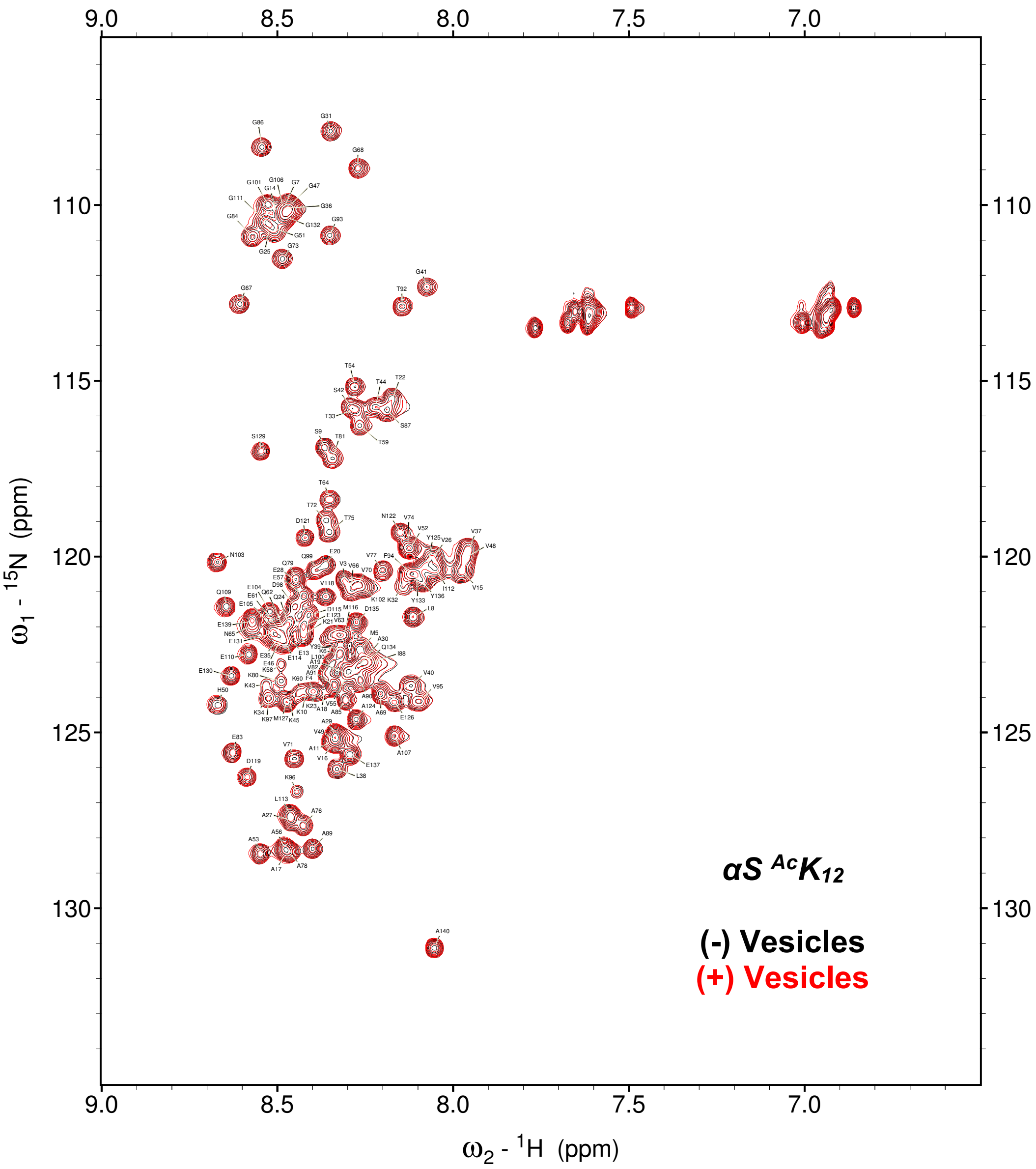

9.0
8.5
8.0
7.5
7.0
110
115
120
125
130
ω1 - 15N (ppm)
ω2 - 1H (ppm)
αS AcK12
(-) Vesicles
(+) Vesicles

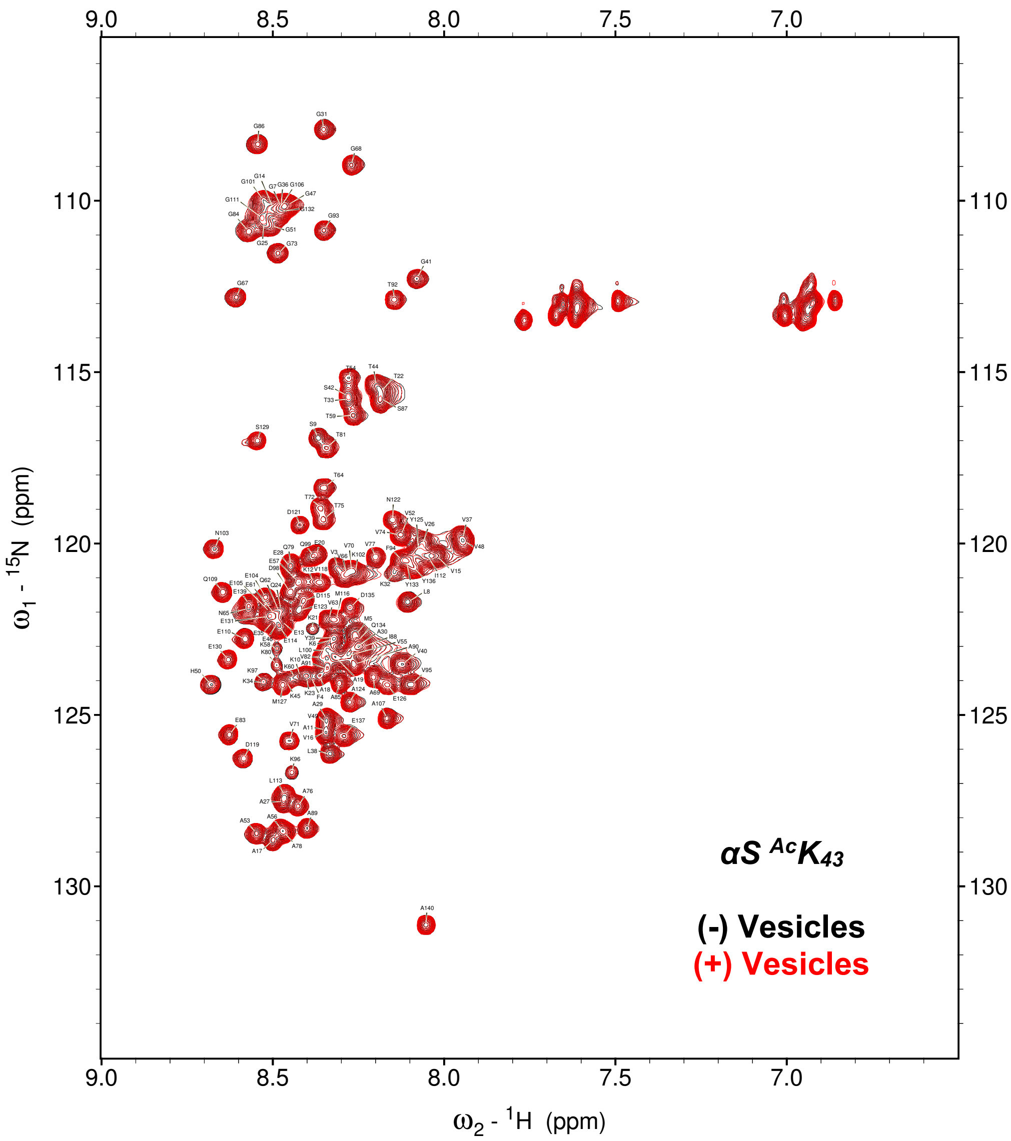

9.0
8.5
8.0
7.5
7.0
110
115
120
125
130
ω1 - 15N (ppm)
ω2 - 1H (ppm)
αS AcK43
(-) Vesicles
(+) Vesicles

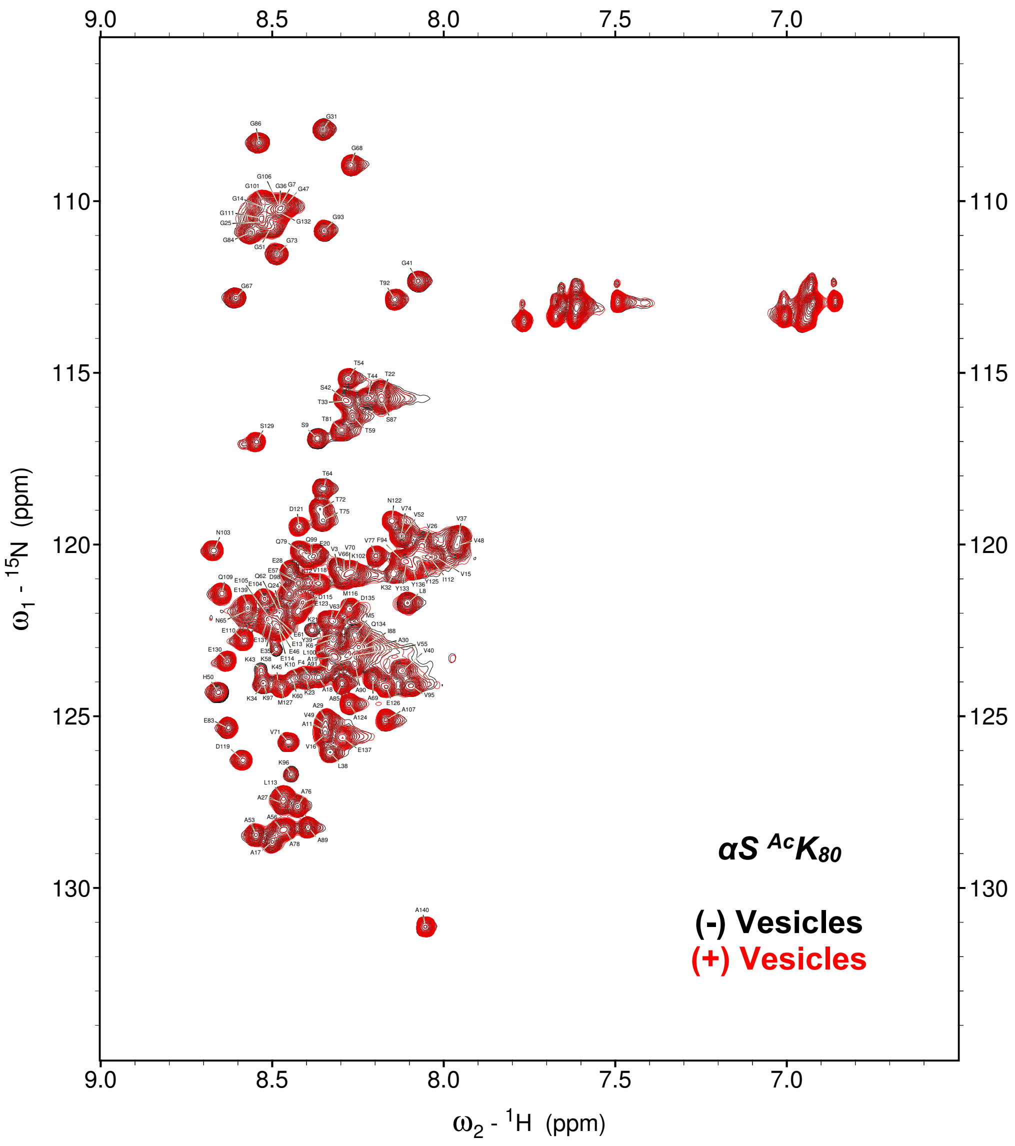

Figure S26. HSQC spectra acquired for vesicle-bound αS-WT, $^{Ac}K_{12}$, $^{Ac}K_{43}$ or $^{Ac}K_{80}$. The spectra of each construct were overlayed with spectra of the corresponding free αS variant.

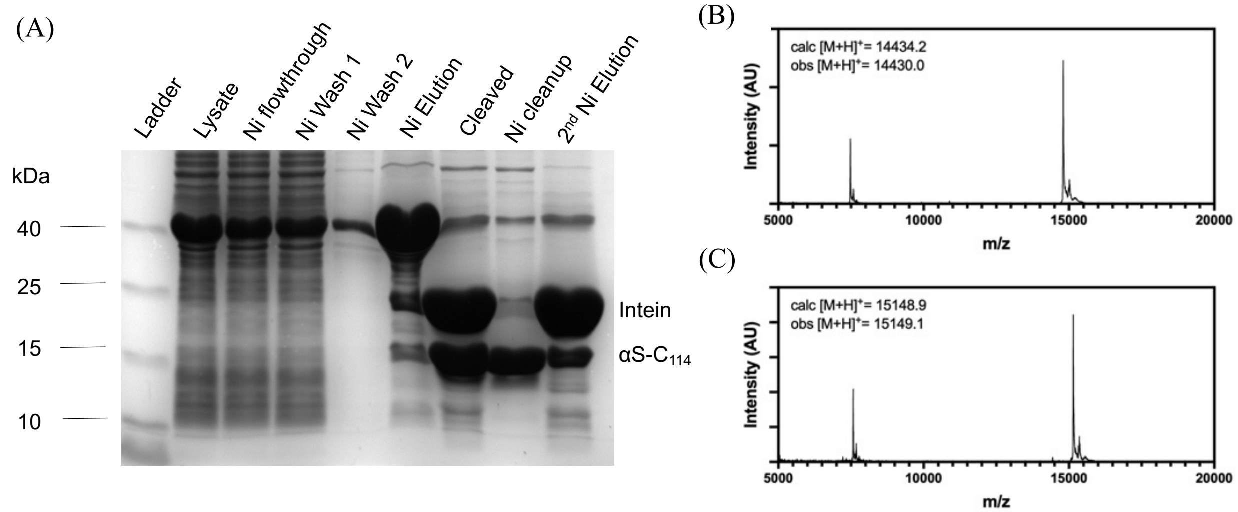

Figure S27. Recombinant αS-C_{114} and fluorescent labeling (a) SDS-PAGE with Coomassie staining to show affinity purification (b) MALDI-MS of purified product αS-C_{114} (c) MALDI-MS of fluorescently labeled, purified product αS-$C^{Atto488}{}_{114}$

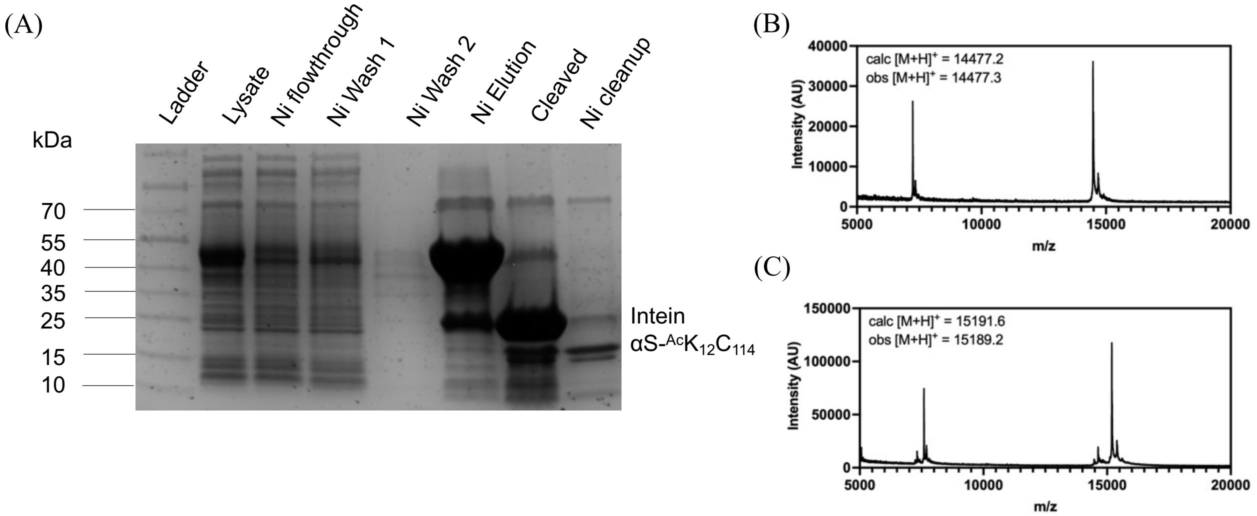

Figure S28. Recombinant αS- ${}^{Ac}K_{12}C_{114}$ and fluorescent labeling (A) SDS-PAGE with Coomassie staining to show affinity purification (B) MALDI-MS of purified product αS- ${}^{Ac}K_{12}C_{114}$ (C) MALDI-MS of fluorescently labeled, purified product αS- ${}^{Ac}K_{12}C^{Atto488}{}_{114}$

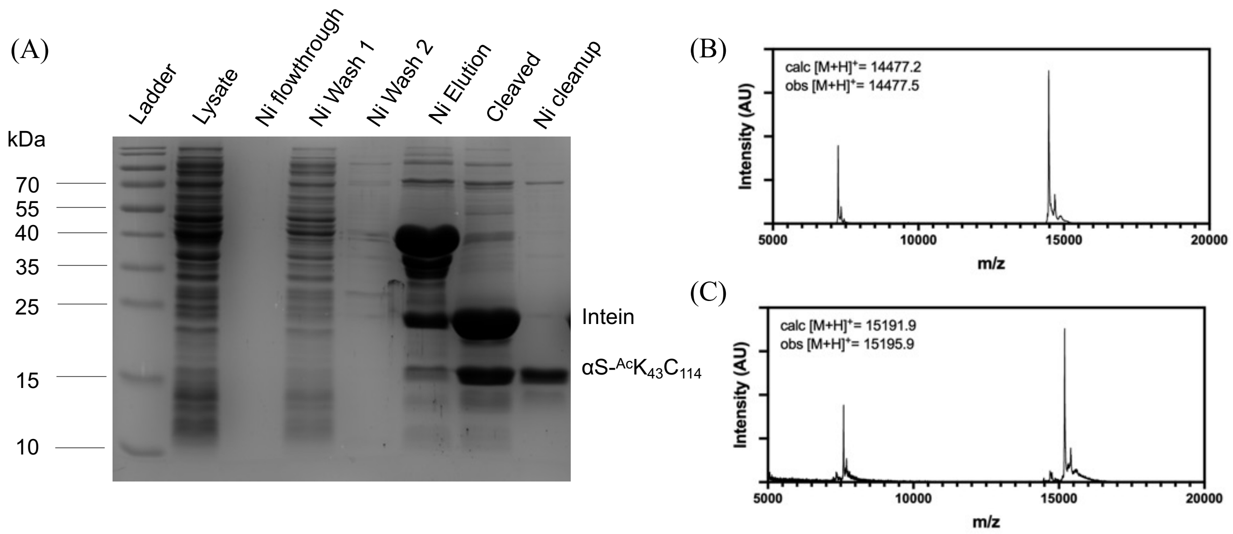

Figure S29. Recombinant αS- $^{Ac}K_{43}C_{114}$ and fluorescent labeling (A) SDS-PAGE with Coomassie staining to show affinity purification (B) MALDI-MS of purified product αS- $^{Ac}K_{43}C_{114}$ (C) MALDI-MS of fluorescently labeled, purified product αS- $^{Ac}K_{43}C^{Atto488}{}_{114}$

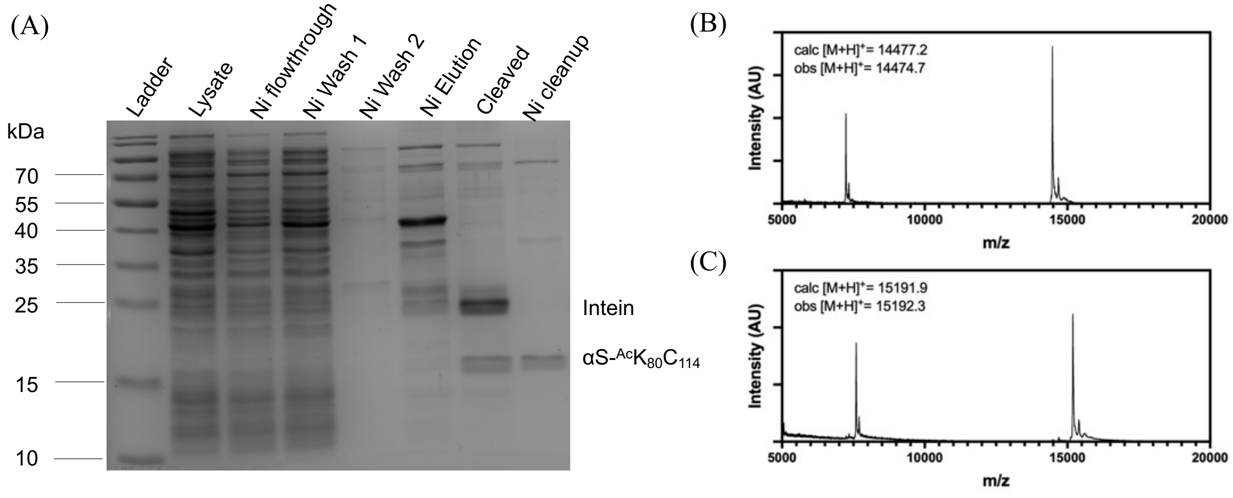

Figure S30. Recombinant αS- $^{Ac}K_{80}C_{114}$ and fluorescent labeling (A) SDS-PAGE with Coomassie staining to show affinity purification (B) MALDI-MS of purified product αS- $^{Ac}K_{80}C_{114}$ (C) MALDI-MS of fluorescently labeled, purified product αS- $^{Ac}K_{80}C^{Atto488}{}_{114}$

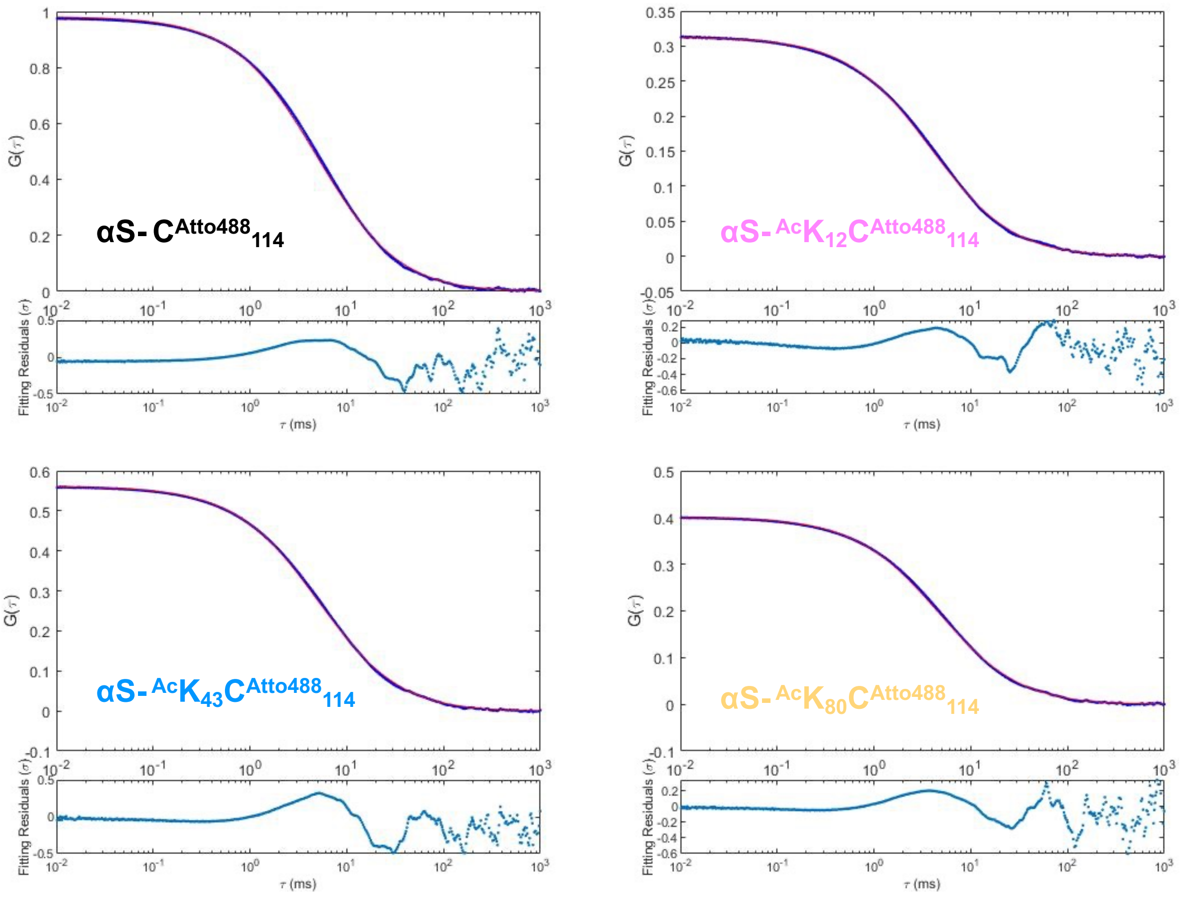

Figure S31. Vesicle FCS data. FCS autocorrelation curves for αS-$C^{Atto488}_{114}$ control (WT), αS-$^{Ac}K_{12}$-$C^{Atto488}_{114}$, αS-$^{Ac}K_{43}$-$C^{Atto488}_{114}$, or αS- $^{Ac}K_{80}C^{Atto488}_{114}$ with 0.1 mM, 50:50 POPS/POPC vesicles. 30 autocorrelation curves were averaged and fit to a single-component autocorrelation function to determine diffusion time.

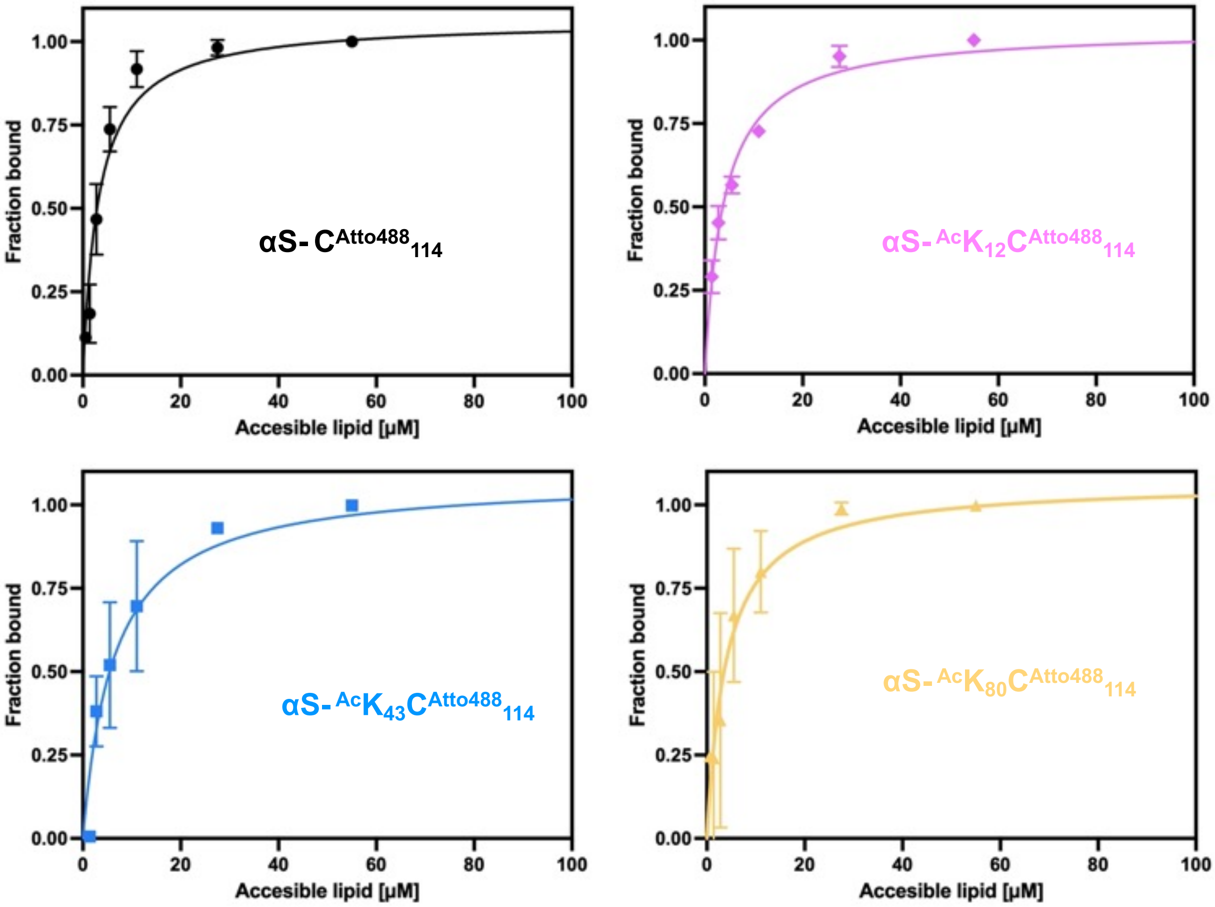

Figure S32. Lipid binding affinity of acetylated αS determined by FCS. Individual binding curves for αS constructs with varying concentrations of 50:50 POPS/POPC vesicles**.**

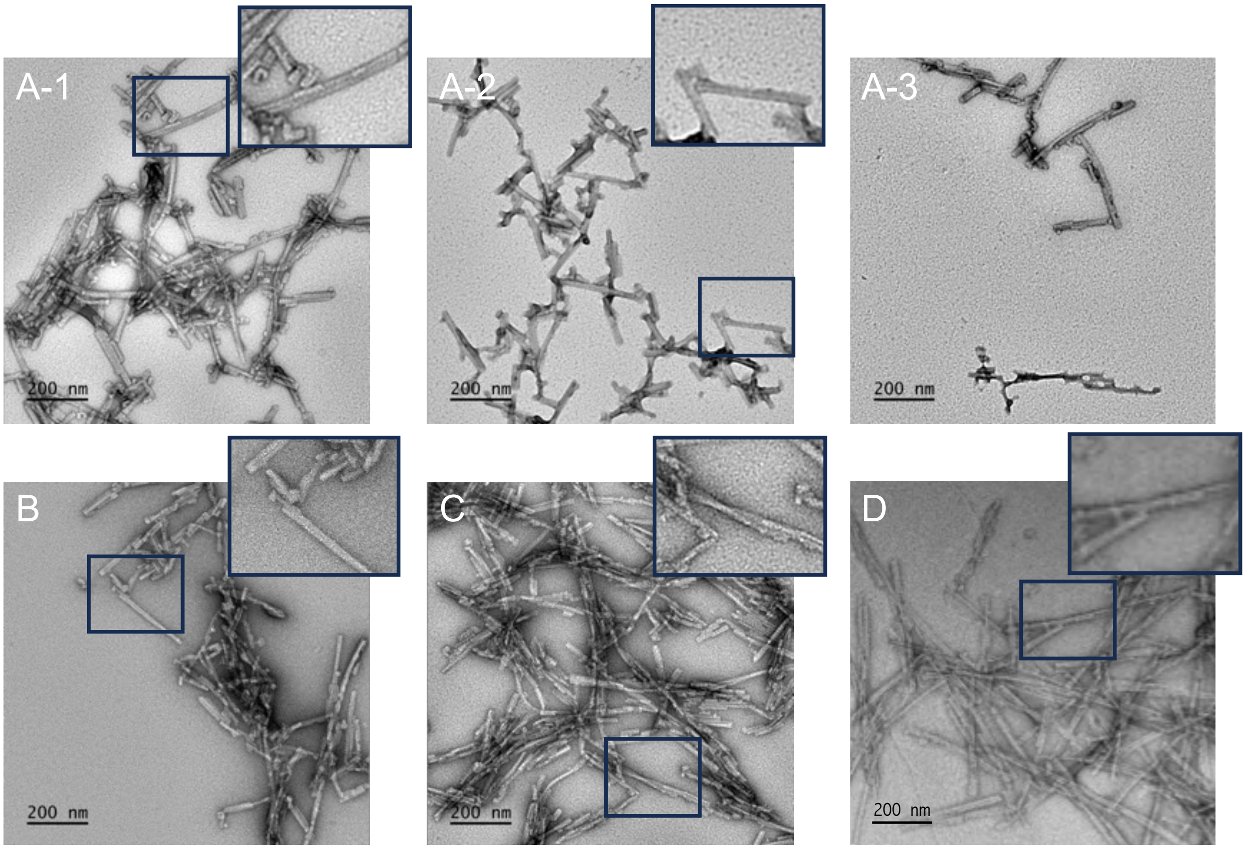

Figure S33. TEM images of ^{Ac}K fibrils. TEM images of fibrils formed from αS monomers comprised of (A-1,A-2,A-3) 25% αS-$^{Ac}K_{12}$ (B) 25% αS-$^{Ac}K_{43}$ (C) 25% αS-$^{Ac}K_{80}$ (D) 100% αS-WT.

Table S2. Statistics of cryo-EM data collection and refinement for 25% $^{Ac}K_{80}$ fibrils

	**25% $^{Ac}K_{80}$ fibrils**
PDB ID	9PT8
EMDB ID	EMD-71835
**Data collection**	
Magnification	100,000
Pixel size (Å)	1.16
Defocus Range (μm)	-2.5 to -0.8
Voltage (kV)	200
Camera	Falcon 4i
Microscope	Talos Glacios
Exposure time (s/fraction)	0.1856
Number of frame	1539
Number of fraction	27
Total dose (e^-/Å^2)	40
**Reconstruction**	
Micrographs	5,387
Box size (pixel)	256
Inter-box distance (Å)	14.3
Segments extracted (no.)	3,364,884
Segments after Class2D (no.)	338,081
Segments after Class3D (no.)	296,473
Resolution (Å)	2.97
Map sharpening B-factor (Å^2)	-85.5827
Helical rise (Å)	4.76
Helical twist (°)	-1.11
**Atomic model**	
Non-hydrogen atoms	4932
Protein residues	60
Ligands	0
r.m.s.d. Bond lengths	0.002
r.m.s.d. Bond angles	0.491
MolProbity score	1.45
All-atom clash score	8.22
Rotamer outliers (%)	0
Ramachandran Outliers (%)	0.00
Ramachandran Allowed (%)	1.72
Ramachandran Favored (%)	98.28
Ramachandran Disallowed (%)	0

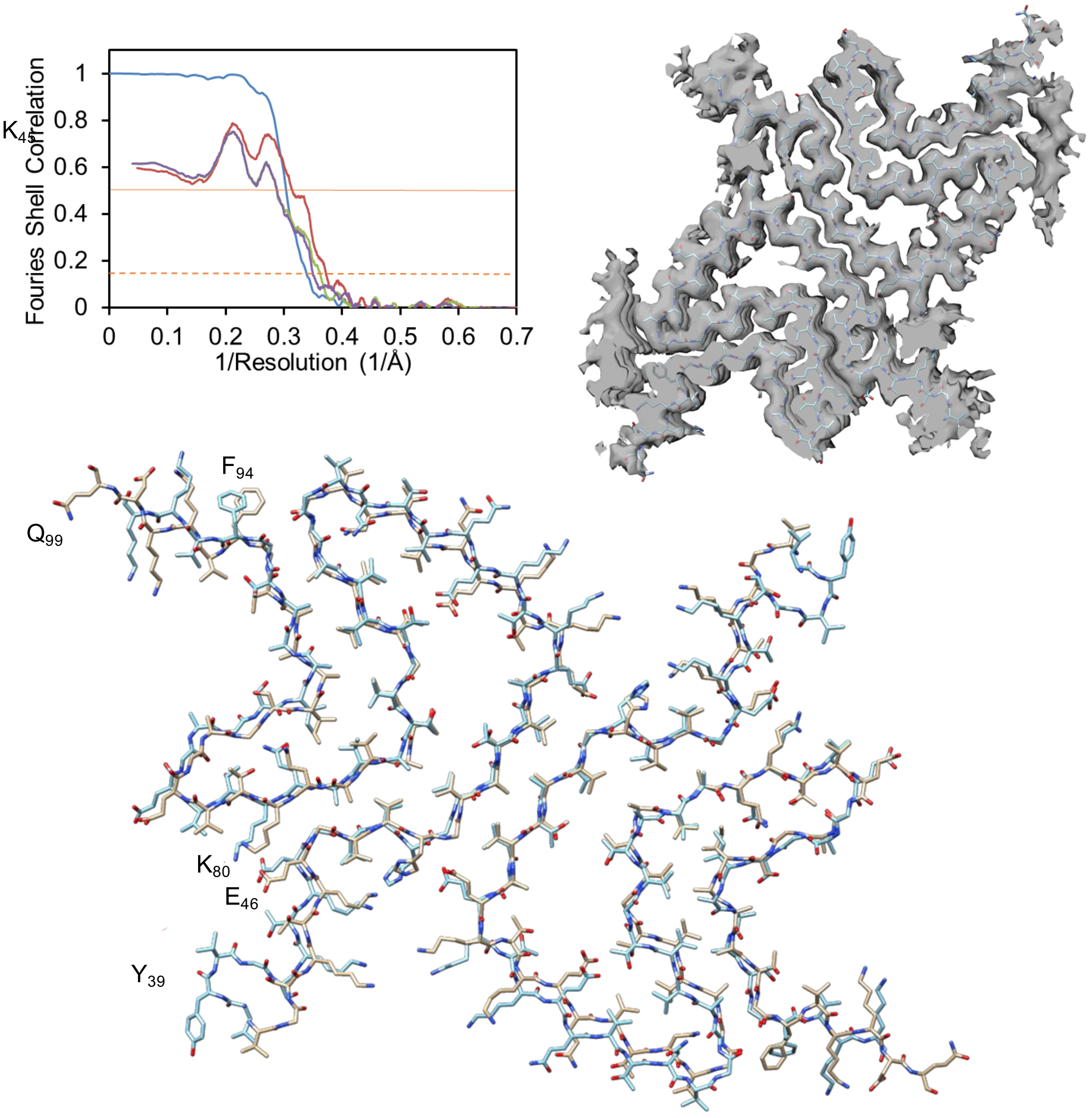

Figure S34. Cryo-EM structure from 25% $^{Ac}K_{80}$ fibril preparation. A. Map and model validation. Fourier shell correlations (FSC) curves for density map and model validation. FSC curve for the density map (FSC-masked, blue), refined models versus full maps (FSC-sum, red), half maps for cross-validation (FSC-work, green and FSC-free, purple). Orange solid line and dash line correspond to FSC values of 0.5 and 0.143, respectively. B. Overlay of the cryo-EM map and the atomic model of 25% αS-$^{Ac}K_{80}$. C. Overlay of 6cu7 (cyan) and 25% αS-$^{Ac}K_{80}$ (light brown).

Table S3. Statistics of cryo-EM data collection and refinement for WT and 100% $^{Ac}K_{80}$ fibrils

	**WT-A**	**WT-B**	**$^{Ac}K_{80}$-A**	**$^{Ac}K_{80}$-B**
PDB ID	9PTA	9PT9	9PTC	9PTB
EMDB ID	EMD-71837	EMD-71836	EMD-71839	EMD-71838
**Data collection**				
Magnification	100,000	100,000	100,000	100,000
Pixel size (Å)	1.16	1.16	1.16	1.16
Defocus Range (μm)	-2.5 to -0.8	-2.5 to -0.8	-2.5 to -0.8	-2.5 to -0.8
Voltage (kV)	200	200	200	200
Camera	Falcon 4i	Falcon 4i	Falcon 4i	Falcon 4i
Microscope	Talos Glacios	Talos Glacios	Talos Glacios	Talos Glacios
Exposure time (s/fraction)	0.1856	0.1856	0.1856	0.1856
Number of frame	1539	1539	1539	1539
Number of fraction	27	27	27	27
Total dose ($e^-/Å^2$)	40	40	40	40
**Reconstruction**				
Micrographs	5001	5001	4038	4038
Box size (pixel)	256	256	256	256
Inter-box distance (Å)	14.3	14.3	14.3	14.3
Segments extracted (no.)	2,341,469	2,341,469	614,398	614,398
Segments after Class3D (no.)	696,830	1,317,407	102,778	200,042
Resolution (Å)	3.00	3.06	3.00	2.94
Map sharpening B-factor ($Å^2$)	-100.97	-121.21	-73.00	-78.18
Helical rise (Å)	-0.83	179.62	-0.88	179.58
Helical twist (°)	4.71	2.36	4.76	2.38
**Atomic model**				
Non-hydrogen atoms	4330	4150	4530	4190
Protein residues	630	620	650	610
Ligands	0	0	0	0
r.m.s.d. Bond lengths	0.005	0.003	0.002	0.002
r.m.s.d. Bond angles	0.497	0.542	0.418	0.469
MolProbity score	2.16	2.1	1.72	2.55
All-atom clash score	10.71	10.65	6.78	11.57
Rotamer outliers (%)	2.27	0	0	4.88
Ramachandran Outliers (%)	0	0	0	0
Ramachandran Allowed (%)	4.92	10.00	5.00	7.14
Ramachandran Favored (%)	95.08	90.00	95.00	92.86

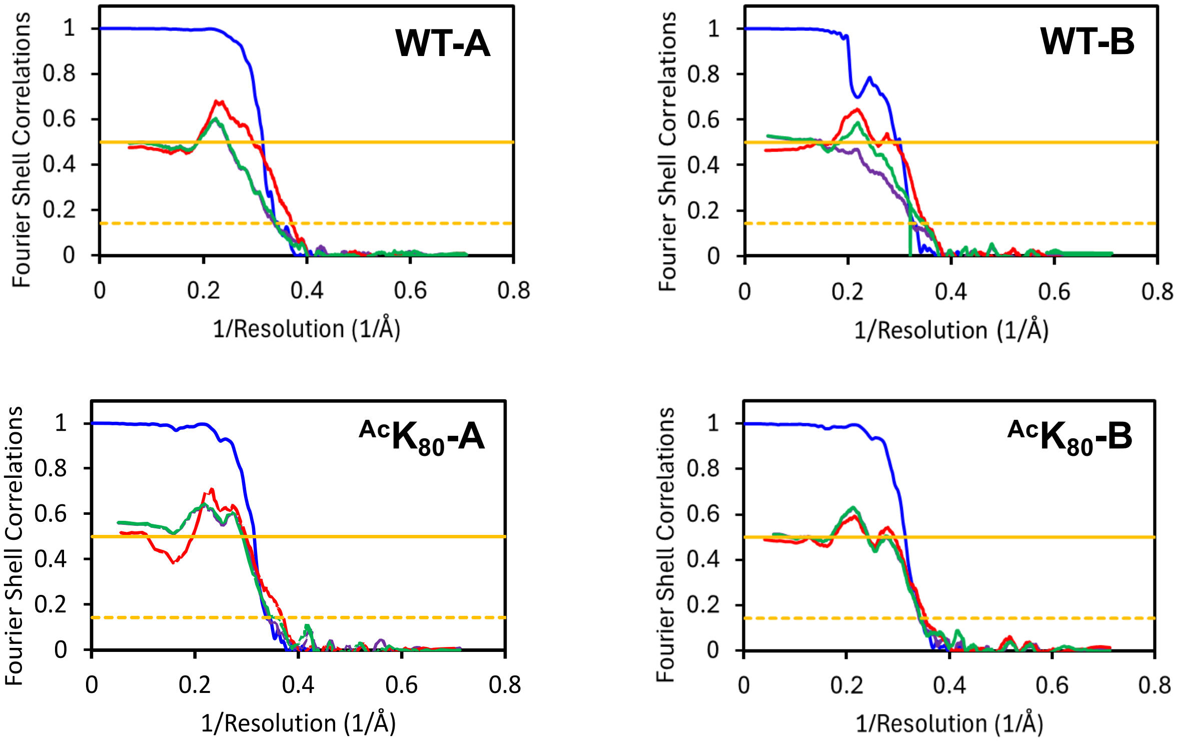

Figure S35. Map and model validation. Fourier shell correlations (FSC) curves for density map and model validation. FSC curve for the density map (FSC-masked, blue), refined models versus full maps (FSC-sum, red), half maps for cross-validation (FSC-work, green and FSC-free, purple). Orange solid line and dash line correspond to FSC values of 0.5 and 0.143, respectively.

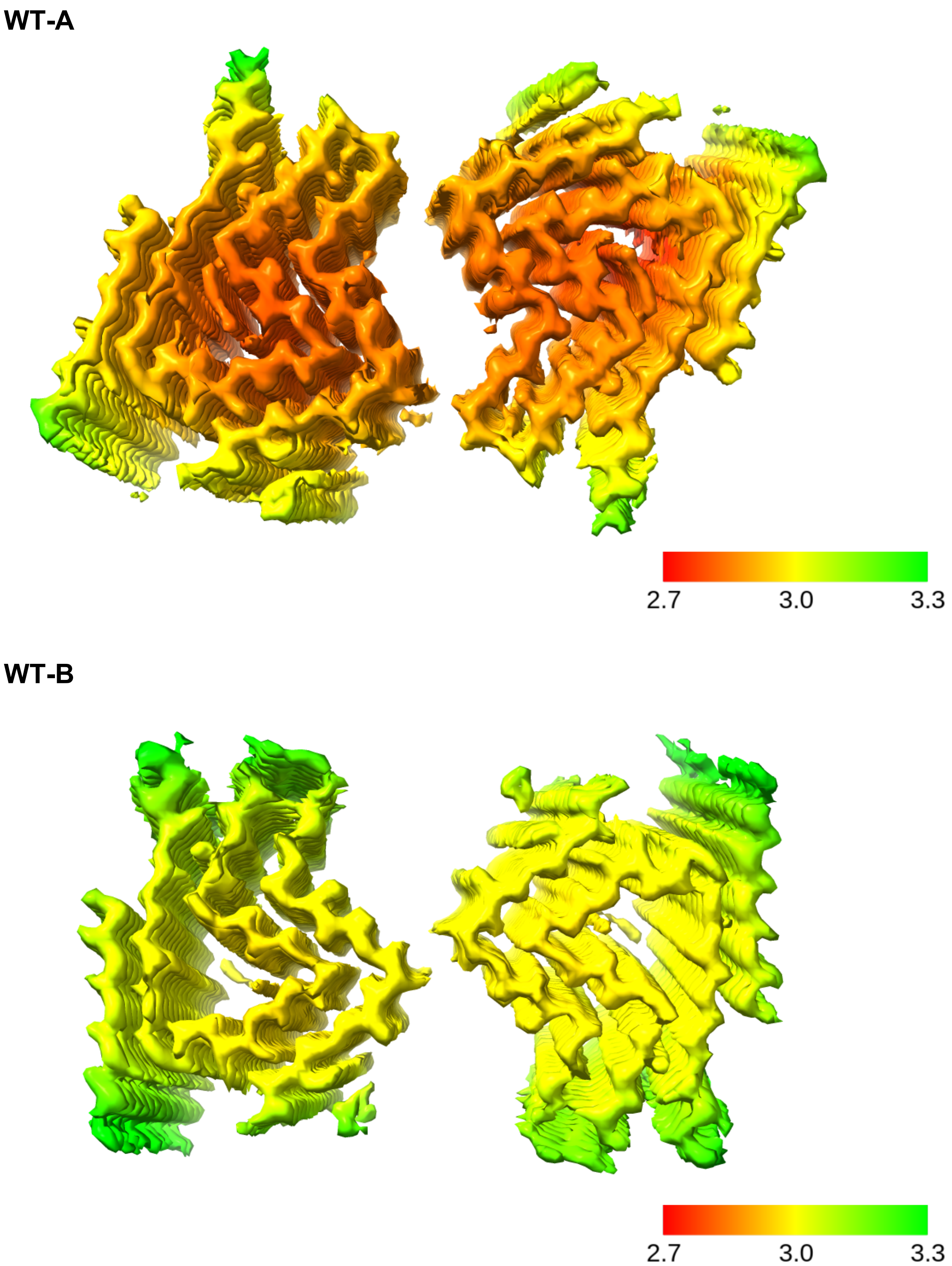

Figure 36. Local resolution cryo-EM maps for WT fibrils. Scale in Å.

$^{Ac}K_{80}$-A

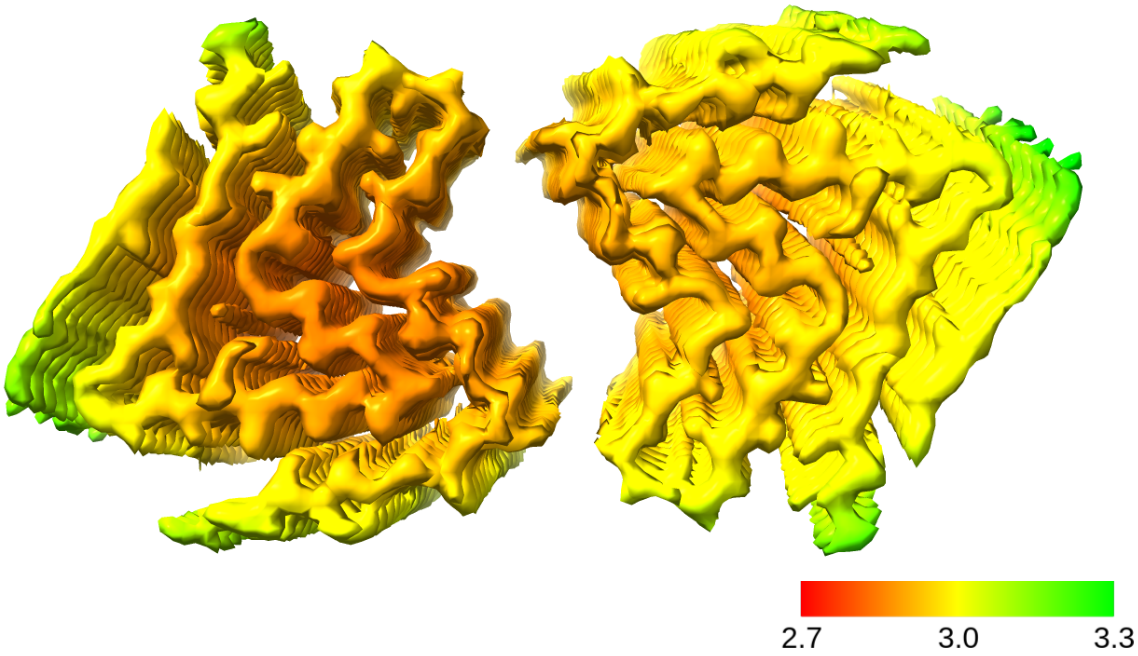

$^{Ac}K_{80}$-B

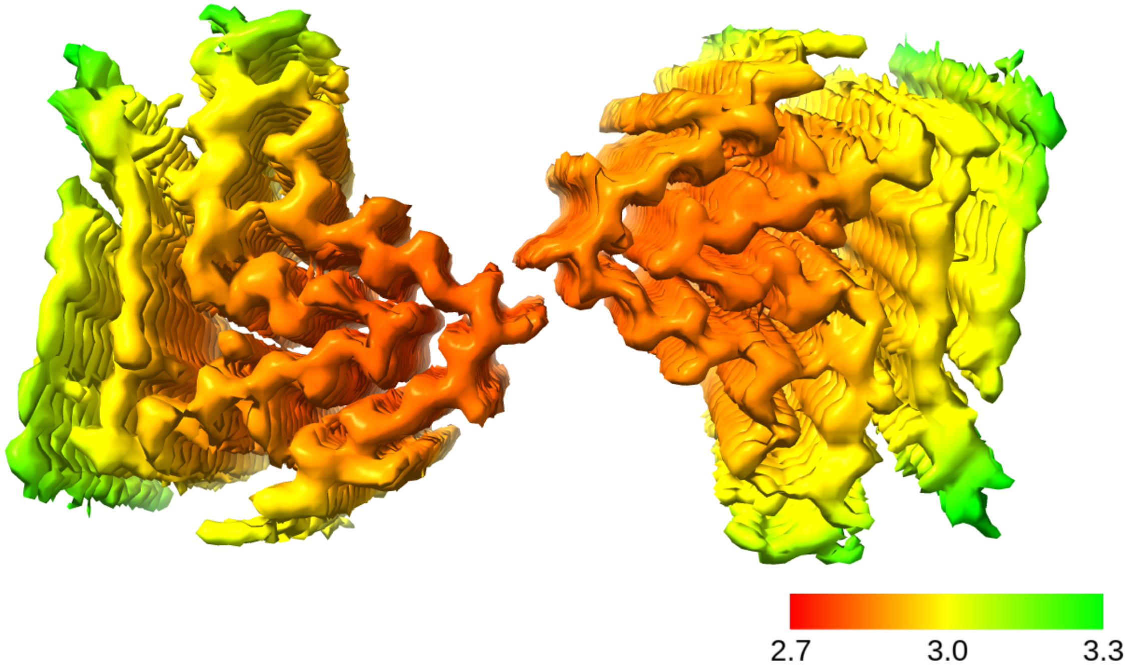

Figure S37. Local resolution cryo-EM maps for $^{Ac}K_{80}$ fibrils. Scale bar in Å.

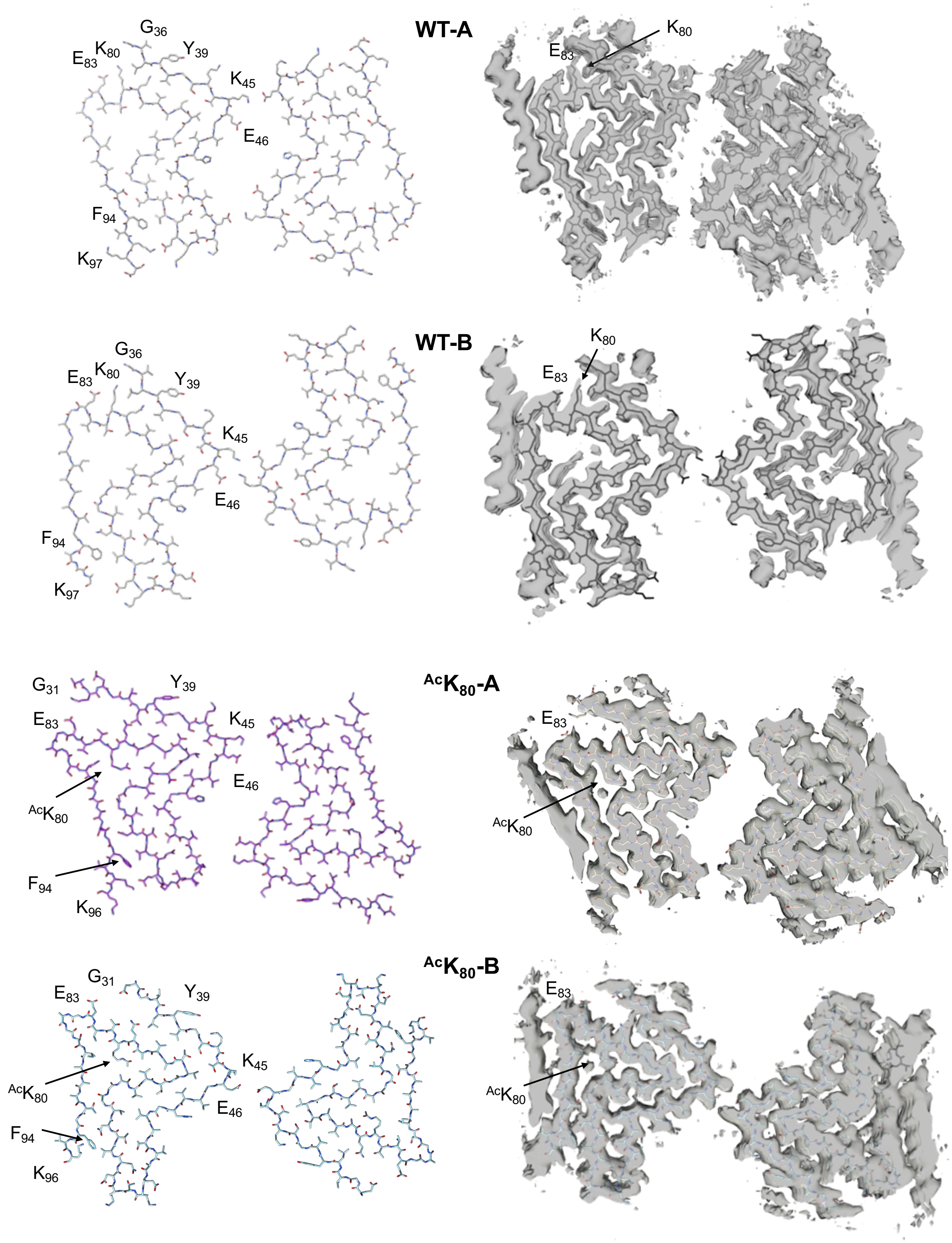

Figure S38. Cryo-EM structures of WT and $^{Ac}K_{80}$ fibrils. Left: Atomic models. Right: Overlays of the cryo-EM maps and the atomic models.

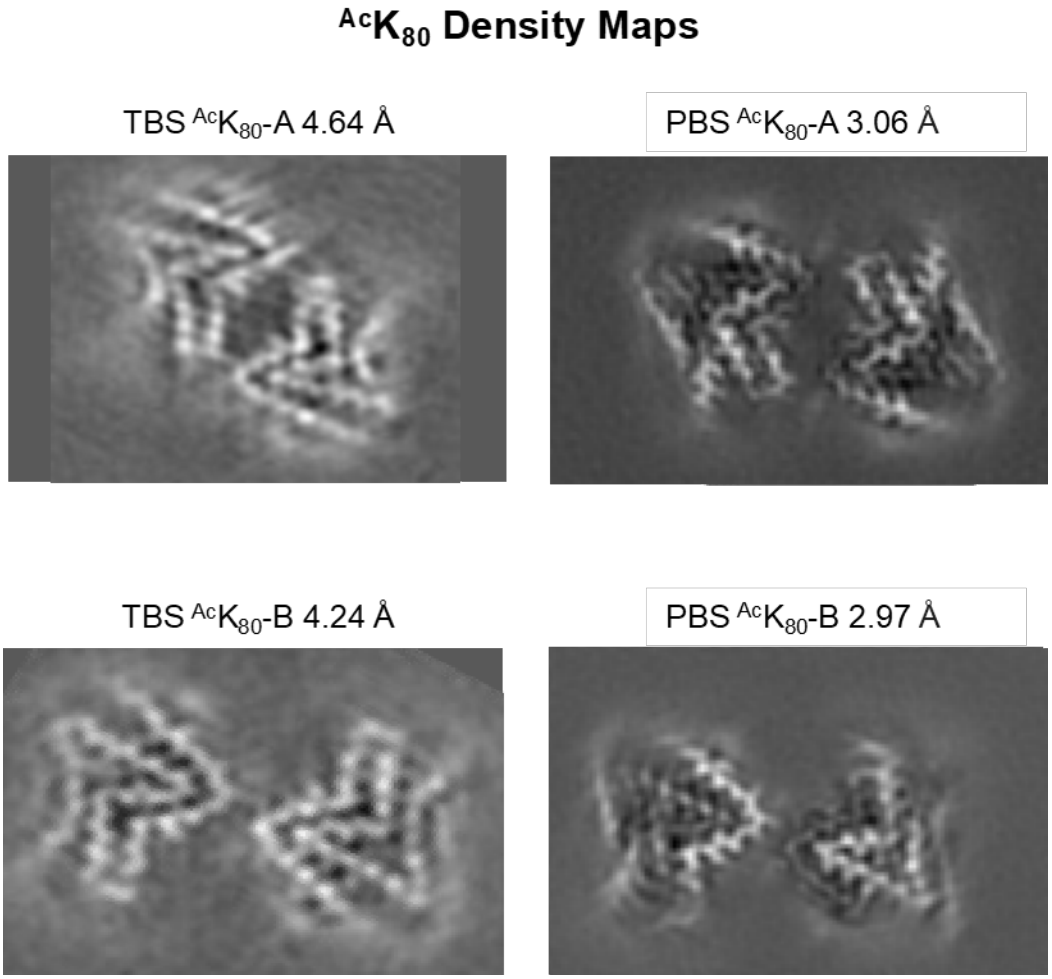

Figure S39. Comparison of cryo-EM density for $^{Ac}K_{80}$ fibrils formed in TBS and PBS.

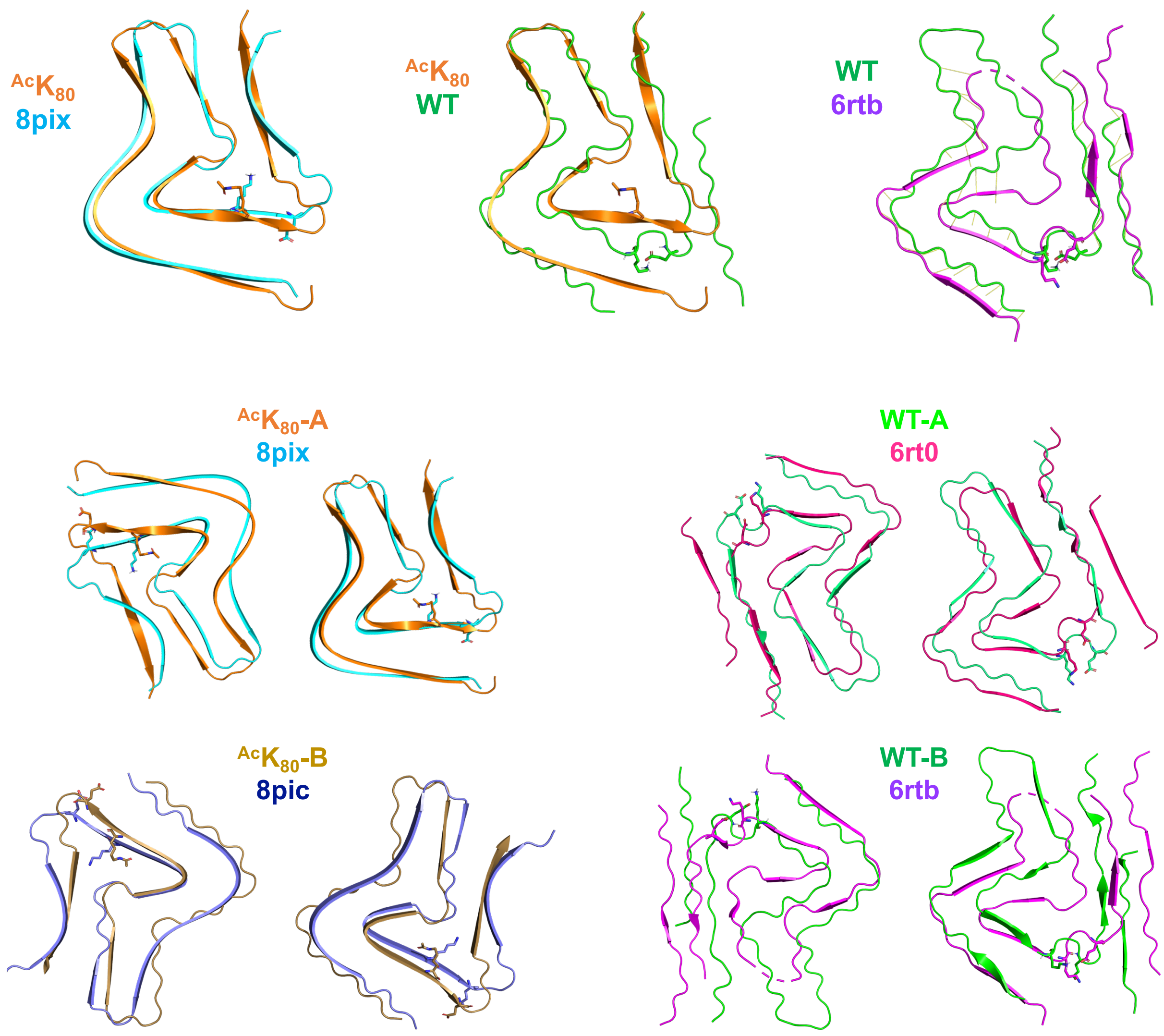

Figure S40. Comparison of $^{Ac}K_{80}$ fibril structure to other cryo-EM structures. Overlays performed using alignment tool in PyMOL show similarity to published fibril polymorphs.(Frey *et al.*, 2024; Guerrero-Ferreira *et al.*, 2019)

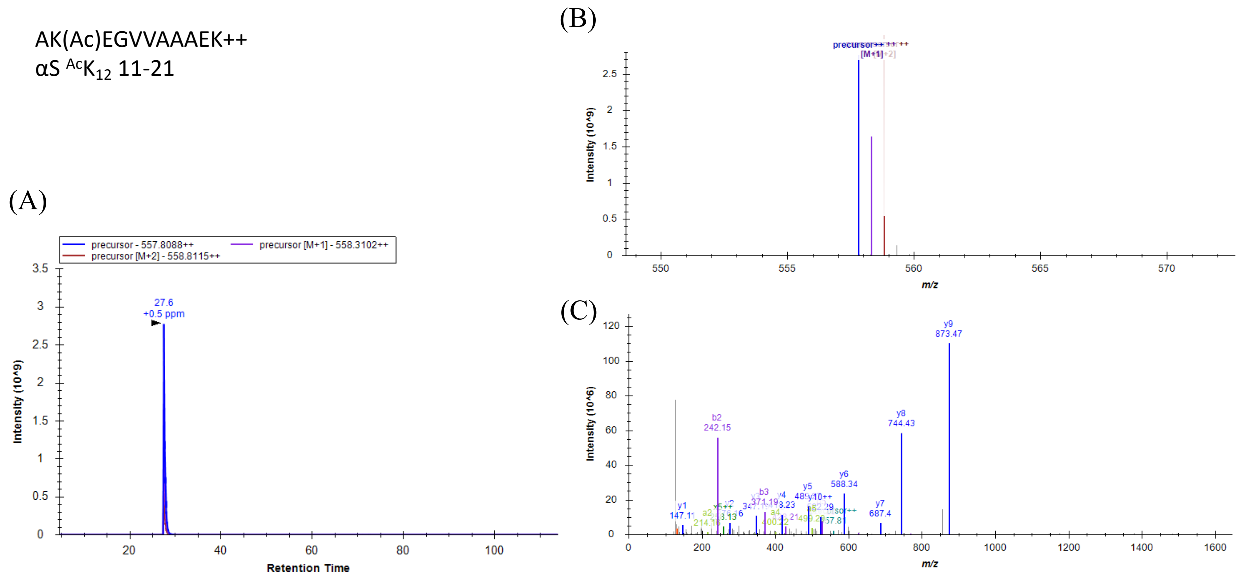

Figure S41. MS spectra of $^{Ac}K_{12}$ peptide from αS $^{Ac}K_{12}$ standard. (A) Extracted ion chromatogram (EIC) (B) MS1 spectrum (C) MS2 spectrum with fragment annotation

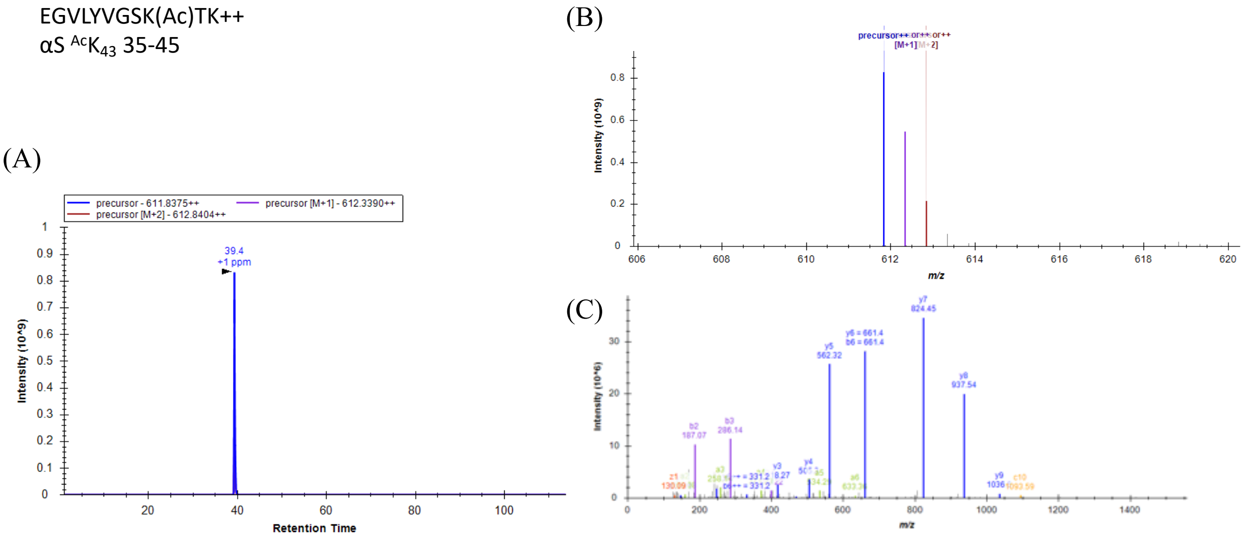

Figure S42. MS spectra of $^{Ac}K_{43}$ peptide from αS $^{Ac}K_{43}$ standard. (A) Extracted ion chromatogram (EIC) (B) MS1 spectrum (C) MS2 spectrum with fragment annotation.

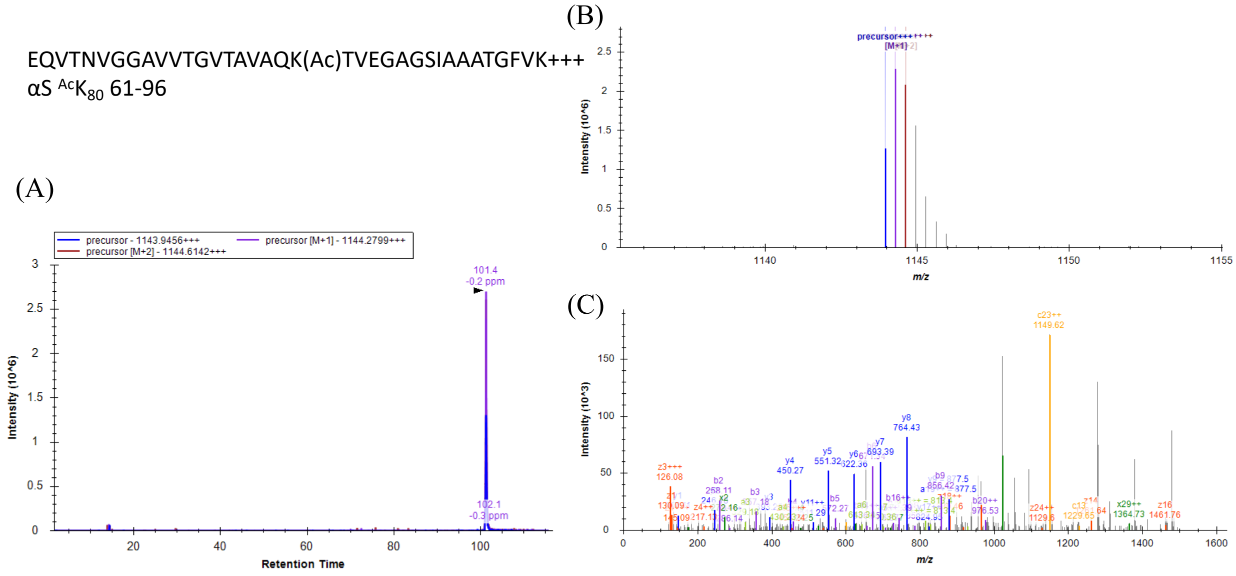

Figure S43. MS spectra of $^{Ac}K_{80}$ peptide from αS $^{Ac}K_{80}$ standard. (A) Extracted ion chromatogram (EIC) (B) MS1 spectrum (C) MS2 spectrum with fragment annotation.

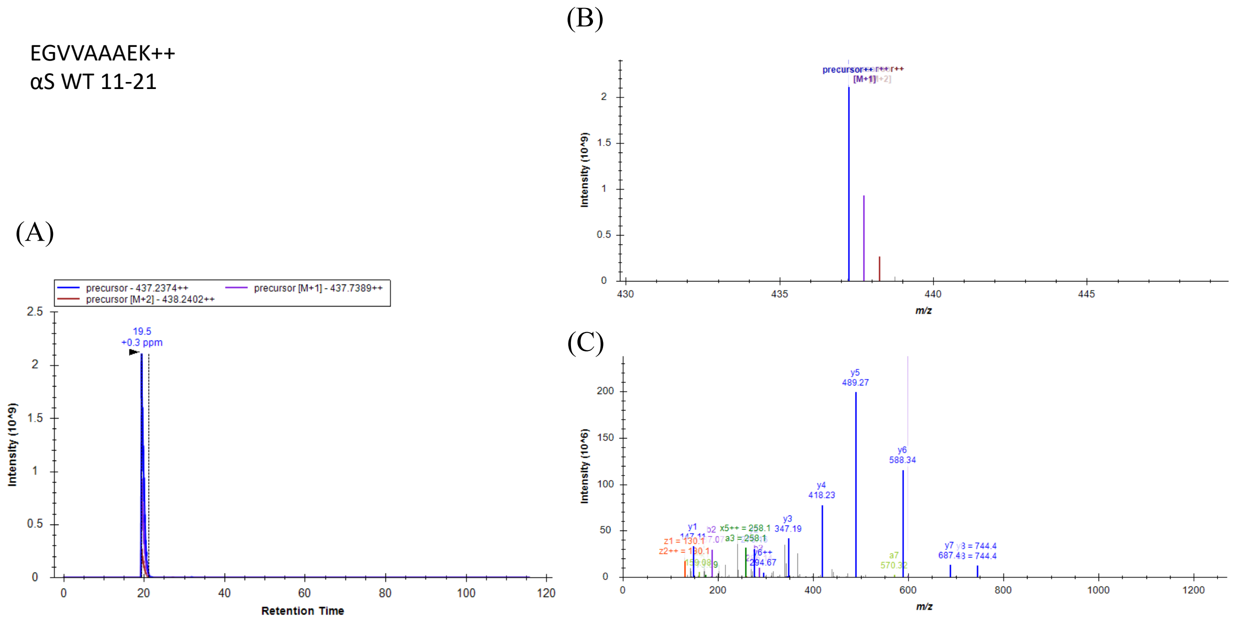

Figure S44. MS spectra of unmodified K_{12} peptide from αS WT standard. (A) Extracted ion chromatogram (EIC) (B) MS1 spectrum (C) MS2 spectrum with fragment annotation.

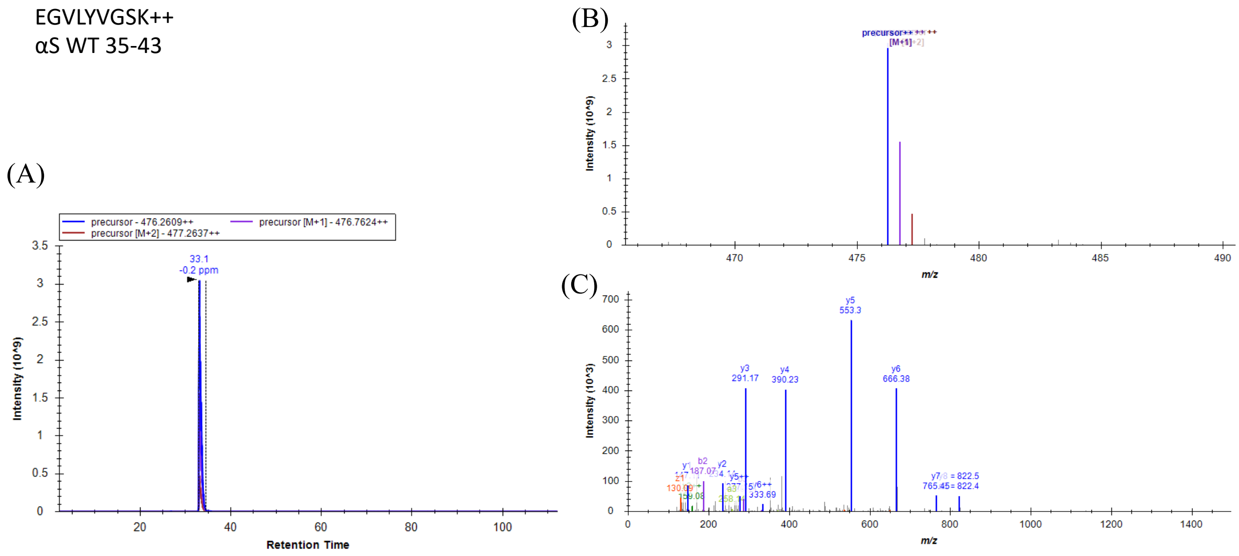

Figure S45. MS spectra of unmodified K_{43} peptide from αS WT standard. (A) Extracted ion chromatogram (EIC) (B) MS1 spectrum (C) MS2 spectrum with fragment annotation.

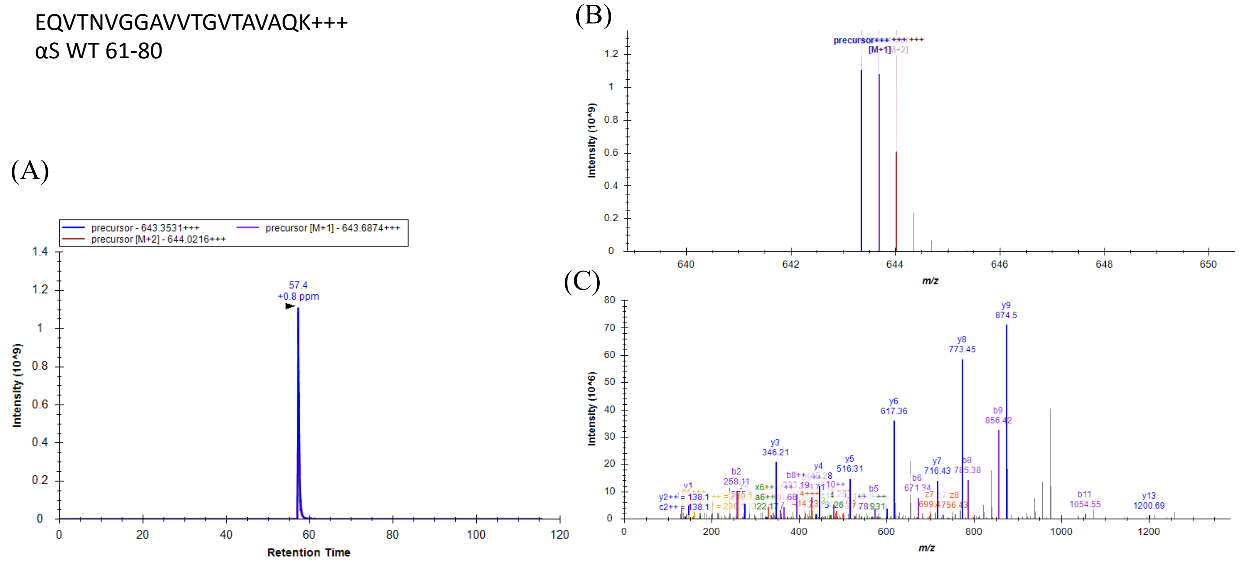

Figure S46. MS spectra of unmodified K_{80} peptide from αS WT standard. (A) Extracted ion chromatogram (EIC) (B) MS1 spectrum (C) MS2 spectrum with fragment annotation.

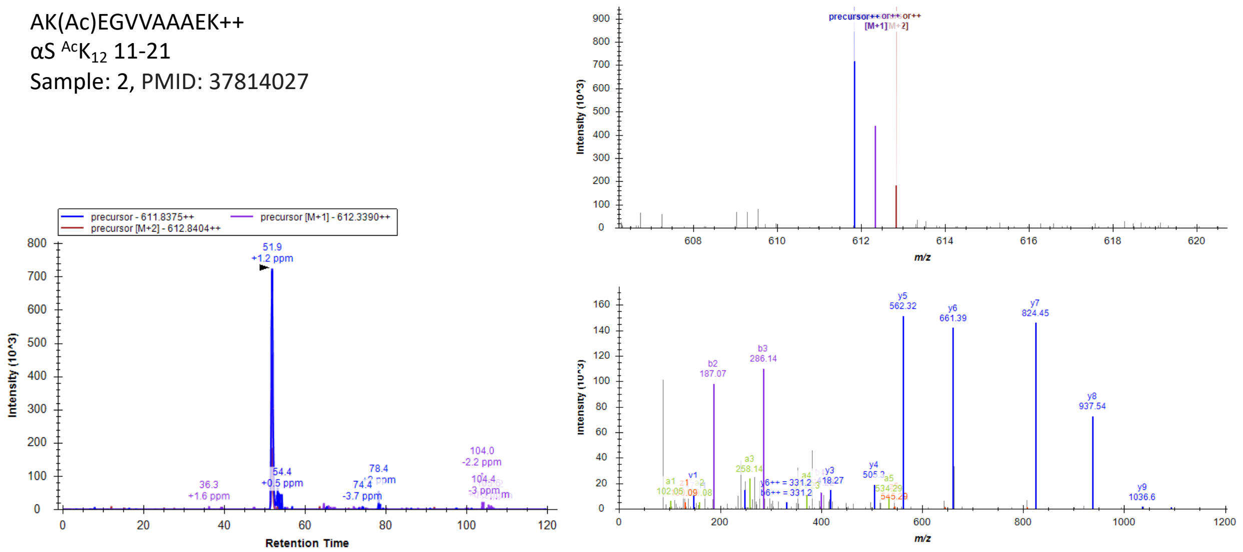

Figure S47. Representative MS spectra of $^{Ac}K_{12}$ peptide from αS in patient sample 2. (A) Extracted ion chromatogram (EIC) (B) MS1 spectrum (C) MS2 spectrum with fragment annotation

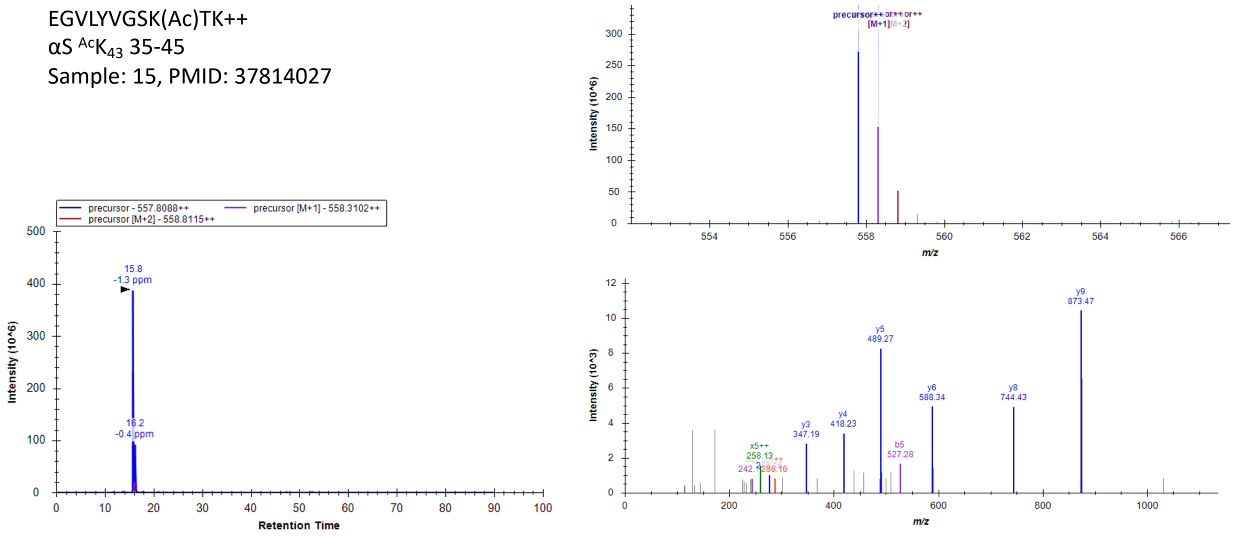

Figure S48. Representative MS spectra of $^{Ac}K_{43}$ peptide from αS in patient sample 15. (A) Extracted ion chromatogram (EIC) (B) MS1 spectrum (C) MS2 spectrum with fragment annotation

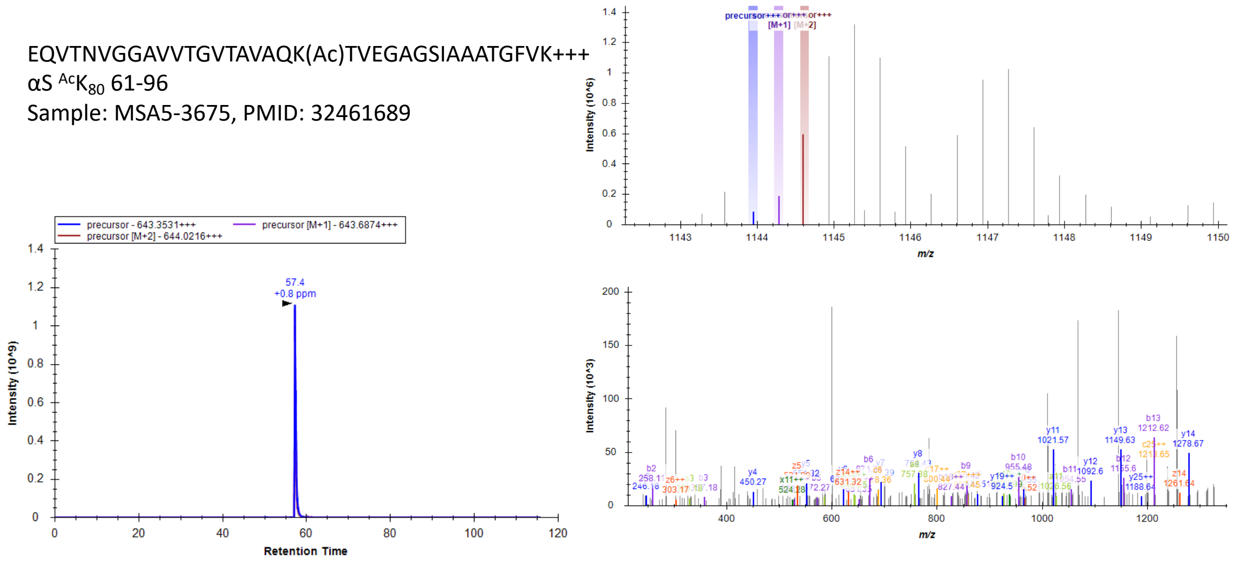

Figure S49. Representative MS spectra of $^{Ac}K_{80}$ peptide from αS in patient sample MSA5-3675. (A) Extracted ion chromatogram (EIC) (B) MS1 spectrum (C) MS2 spectrum with fragment annotation

Table S4. Quantification of acetylation %ratio by LC-MS/MS data analysis

αS $^{Ac}K_{12}$

Case no	Sample no	Pathological diagnosis	Clinical diagnosis	Soluble or insoluble	%ratio
5	2	PDD	PDD	Soluble	10.33%
5	7	PDD	PDD	Soluble	4.34%
8	11	AD	AD probable	Soluble	1.79%
9	16	AD	CBS	Soluble	0.09%
10	19	MSA	MSA-C	Soluble	1.93%
10	20	MSA	MSA-C	Soluble	1.91%
13	29	MSA	MSA-P	Soluble	0.01%
13	30	MSA	MSA-P	Soluble	3.85%
13	31	MSA	MSA-P	Soluble	7.63%
19	36	Pathological aging	Normal	Soluble	0.01%
21	37	Primary age-related tauopathy	Normal	Soluble	29.29%
21	38	Primary age-related tauopathy	Normal	Soluble	10.02%
23	40	Normal	Normal	Soluble	8.62%

αS $^{Ac}K_{43}$

Case no	Sample no	Pathological diagnosis	Clinical diagnosis	Soluble or insoluble	%ratio
9	15	AD	CBS	Soluble	38.81%
9	16	AD	CBS	Soluble	0.09%
9	17	AD	CBS	Soluble	10.44%
9	18	AD	CBS	Soluble	0.00%
13	28	MSA	MSA-P	Soluble	10.26%
13	29	MSA	MSA-P	Soluble	0.00%
14	31	MSA	MSA-C	Soluble	0.04%
19	35	Pathological aging	Normal	Soluble	13.10%
21	37	Primary age-related tauopathy	Normal	Soluble	0.02%

αS $^{Ac}K_{80}$

Case no	Sample no	Pathological diagnosis	Clinical diagnosis	Soluble or insoluble	%ratio
3	MSA3-9665	MSA	MSA-C	Insoluble	37.77%
5	MSA5-3675	MSA	MSA-C	Insoluble	41.77%

Table S5. Ionization Efficiency of Acetylated Tryptic Peptides

Peptide	Entity	Quantification	Ionization efficiency
EGVVAAAEK	αS WT $_{13\text{-}21}$	1.16E+11	
EGVLYVGSK	αS WT $_{35\text{-}43}$	1.39E+11	
EQVTNVGGAVVTGVTAVAQK	αS WT $_{61\text{-}80}$	4.96E+10	
AK[+42.011]EGVVAAAEK	αS $^{Ac}K_{12\ 11\text{-}21}$	3.90E+11	3.37
EGVLYVGSK[+42.011]TK	αS $^{Ac}K_{43\ 35\text{-}45}$	1.22E+11	0.88
EQVTNVGGAVVTGVTAVAQK[+42.011]TVEGAGSIAAATGFVK	αS $^{Ac}K_{80\ 61\text{-}96}$	2.18E+08	0.004387

References

Anderson, E.H. (1946). Growth Requirements of Virus-Resistant Mutants of Escherichia Coli Strain "B". Proc Natl Acad Sci U S A *32*, 120-128. 10.1073/pnas.32.5.120.

Batjargal, S., Walters, C.R., and Petersson, E.J. (2015). Inteins as Traceless Purification Tags for Unnatural Amino Acid Proteins. J. Am. Chem. Soc. *137*, 1734-1737. 10.1021/ja5103019.

Burt, A., Toader, B., Warshamanage, R., von Kügelgen, A., Pyle, E., Zivanov, J., Kimanius, D., Bharat, T.A.M., and Scheres, S.H.W. (2024). An image processing pipeline for electron cryo-tomography in RELION-5. FEBS Open Bio *14*, 1788-1804. https://doi.org/10.1002/2211-5463.13873.

Decroos, C., Bowman, C.M., Moser, J.A., Christianson, K.E., Deardorff, M.A., and Christianson, D.W. (2014). Compromised structure and function of HDAC8 mutants identified in Cornelia de Lange Syndrome spectrum disorders. ACS Chem Biol *9*, 2157-2164. 10.1021/cb5003762.

Delaglio, F., Grzesiek, S., Vuister, G.W., Zhu, G., Pfeifer, J., and Bax, A. (1995). NMRPipe: A multidimensional spectral processing system based on UNIX pipes. Journal of Biomolecular NMR *6*, 277-293. 10.1007/BF00197809.

Dikiy, I., and Eliezer, D. (2014). N-terminal acetylation stabilizes N-terminal helicity in lipid- and micelle-bound α-synuclein and increases its affinity for physiological membranes. J Biol Chem *289*, 3652-3665. 10.1074/jbc.M113.512459.

Dowling, D.P., Gantt, S.L., Gattis, S.G., Fierke, C.A., and Christianson, D.W. (2008). Structural studies of human histone deacetylase 8 and its site-specific variants complexed with substrate and inhibitors. Biochemistry *47*, 13554-13563. 10.1021/bi801610c.

Eliezer, D., Kutluay, E., Bussell, R., and Browne, G. (2001). Conformational properties of α-synuclein in its free and lipid-associated states11Edited by P. E. Wright. Journal of Molecular Biology *307*, 1061-1073. https://doi.org/10.1006/jmbi.2001.4538.

Frey, L., Ghosh, D., Qureshi, B.M., Rhyner, D., Guerrero-Ferreira, R., Pokharna, A., Kwiatkowski, W., Serdiuk, T., Picotti, P., Riek, R., and Greenwald, J. (2024). On the pH-dependence of α-synuclein amyloid polymorphism and the role of secondary nucleation in seed-based amyloid propagation. eLife *12*, RP93562. 10.7554/eLife.93562.

Guerrero-Ferreira, R., Taylor, N.M.I., Arteni, A.-A., Kumari, P., Mona, D., Ringler, P., Britschgi, M., Lauer, M.E., Makky, A., Verasdonck, J., et al. (2019). Two new polymorphic structures of human full-length alpha-synuclein fibrils solved by cryo-electron microscopy. eLife *8*, e48907. 10.7554/eLife.48907.

Haase, C., Rohde, H., and Seitz, O. (2008). Native chemical ligation at valine. Angew. Chem.-Int. Edit. *47*, 6807-6810. 10.1002/anie.200801590.

Huang, Y.C., Chen, C.C., Gao, S., Wang, Y.H., Xiao, H., Wang, F., Tian, C.L., and Li, Y.M. (2016). Synthesis of L- and D-Ubiquitin by One-Pot Ligation and Metal-Free Desulfurization. Chem.-Eur. J. *22*, 7623-7628. 10.1002/chem.201600101.

Kimanius, D., Dong, L., Sharov, G., Nakane, T., and Scheres, S.H.W. (2021). New tools for automated cryo-EM single-particle analysis in RELION-4.0. Biochemical Journal *478*, 4169-4185. 10.1042/BCJ20210708.

Lee, W., Tonelli, M., and Markley, J.L. (2015). NMRFAM-SPARKY: enhanced software for biomolecular NMR spectroscopy. Bioinformatics *31*, 1325-1327. 10.1093/bioinformatics/btu830.

Li, Y., Zhao, C., Luo, F., Liu, Z., Gui, X., Luo, Z., Zhang, X., Li, D., Liu, C., and Li, X. (2018). Amyloid fibril structure of α-synuclein determined by cryo-electron microscopy. Cell Research *28*, 897-903. 10.1038/s41422-018-0075-x.
Liebschner, D., Afonine, P.V., Baker, M.L., Bunkoczi, G., Chen, V.B., Croll, T.I., Hintze, B., Hung, L.-W., Jain, S., McCoy, A.J., et al. (2019). Macromolecular structure determination using X-rays, neutrons and electrons: recent developments in Phenix. Acta Crystallographica Section D *75*, 861-877. doi:10.1107/S2059798319011471.
Liu, J., Chan, K.K.J., and Chan, W. (2016). Identification of Protein Thiazolidination as a Novel Molecular Signature for Oxidative Stress and Formaldehyde Exposure. Chemical Research in Toxicology *29*, 1865-1871. 10.1021/acs.chemrestox.6b00271.
Lövestam, S., and Scheres, S.H.W. (2022). High-throughput cryo-EM structure determination of amyloids. Faraday Discussions *240*, 243-260. 10.1039/D2FD00034B.
Maciejewski, M.W., Schuyler, A.D., Gryk, M.R., Moraru, I.I., Romero, P.R., Ulrich, E.L., Eghbalnia, H.R., Livny, M., Delaglio, F., and Hoch, J.C. (2017). NMRbox: A Resource for Biomolecular NMR Computation. Biophys. J. *112*, 1529-1534. 10.1016/j.bpj.2017.03.011.
Muir, T.W., Sondhi, D., and Cole, P.A. (1998). Expressed protein ligation: A general method for protein engineering. Proc. Natl. Acad. Sci. U. S. A. *95*, 6705-6710. 10.1073/pnas.95.12.6705.
Osko, J.D., Porter, N.J., Decroos, C., Lee, M.S., Watson, P.R., Raible, S.E., Krantz, I.D., Deardorff, M.A., and Christianson, D.W. (2021). Structural analysis of histone deacetylase 8 mutants associated with Cornelia de Lange Syndrome spectrum disorders. J Struct Biol *213*, 107681. 10.1016/j.jsb.2020.107681.
Pan, B., Kamo, N., Shimogawa, M., Huang, Y., Kashina, A., Rhoades, E., and Petersson, E.J. (2020). Effects of Glutamate Arginylation on α-Synuclein: Studying an Unusual Post-Translational Modification through Semisynthesis. Journal of the American Chemical Society *142*, 21786-21798. 10.1021/jacs.0c10054.
Punjani, A., Rubinstein, J.L., Fleet, D.J., and Brubaker, M.A. (2017). cryoSPARC: algorithms for rapid unsupervised cryo-EM structure determination. Nature Methods *14*, 290-296. 10.1038/nmeth.4169.
Schweighauser, M., Shi, Y., Tarutani, A., Kametani, F., Murzin, A.G., Ghetti, B., Matsubara, T., Tomita, T., Ando, T., Hasegawa, K., et al. (2020). Structures of α-synuclein filaments from multiple system atrophy. Nature. 10.1038/s41586-020-2317-6.
Volpicelli-Daley, Laura A., Luk, Kelvin C., Patel, Tapan P., Tanik, Selcuk A., Riddle, Dawn M., Stieber, A., Meaney, David F., Trojanowski, John Q., and Lee, Virginia M.Y. (2011). Exogenous α-Synuclein Fibrils Induce Lewy Body Pathology Leading to Synaptic Dysfunction and Neuron Death. Neuron *72*, 57-71. 10.1016/j.neuron.2011.08.033.
Wagner, T., Merino, F., Stabrin, M., Moriya, T., Antoni, C., Apelbaum, A., Hagel, P., Sitsel, O., Raisch, T., Prumbaum, D., et al. (2019). SPHIRE-crYOLO is a fast and accurate fully automated particle picker for cryo-EM. Communications Biology *2*, 218. 10.1038/s42003-019-0437-z.
Waxman, E.A., and Giasson, B.I. (2008). Specificity and Regulation of Casein Kinase-Mediated Phosphorylation of α-Synuclein. Journal of Neuropathology & Experimental Neurology *67*, 402-416. 10.1097/NEN.0b013e3186fc995.
Xiao, Q., Zhang, F., Nacev, B.A., Liu, J.O., and Pei, D. (2010). Protein N-terminal processing: substrate specificity of Escherichia coli and human methionine aminopeptidases. Biochemistry *49*, 5588-5599. 10.1021/bi1005464.
Zhang, S., Zhu, R., Pan, B., Xu, H., Olufemi, M.F., Gathagan, R.J., Li, Y., Zhang, L., Zhang, J., Xiang, W., et al. (2023). Post-translational modifications of soluble α-synuclein regulate the

amplification of pathological α-synuclein. Nat Neurosci *26*, 213-225. 10.1038/s41593-022-01239-7.
